# ApoE isoform-dependent effects of xanthohumol on high fat diet-induced cognitive impairments and hippocampal metabolic pathways

**DOI:** 10.3389/fphar.2022.954980

**Published:** 2022-10-03

**Authors:** Payel Kundu, Sarah Holden, Ines L. Paraiso, Reetesh Sudhakar, Chloe McQuesten, Jaewoo Choi, Cristobal L. Miranda, Claudia S. Maier, Gerd Bobe, Jan F. Stevens, Jacob Raber

**Affiliations:** ^1^ Department of Behavioral Neuroscience, Oregon Health & Science University, Portland, OR, United States; ^2^ Linus Pauling Institute, Oregon State University, Corvallis, OR, United States; ^3^ Department of Pharmaceutical Sciences, Oregon State University, Corvallis, OR, United States; ^4^ Department of Chemistry, Oregon State University, Corvallis, OR, United States; ^5^ Department of Animal and Rangeland Sciences, Oregon State University, Corvallis, OR, United States; ^6^ Departments of Neurology and Radiation Medicine, Division of Neuroscience, Oregon National Primate Research Center, Oregon Health & Science University, Portland, OR, United States

**Keywords:** xanthohumol, high-fat diet, cognition, metabolic syndrome, APOE, metabolomics, glucose transporters, hippocampus

## Abstract

Consumption of a high fat diet (HFD) is linked to metabolic syndrome and cognitive impairments. This is exacerbated in age-related cognitive decline (ACD) and in individuals with a genetic risk for Alzheimer’s disease (AD). Apolipoprotein E (apoE) is involved in cholesterol metabolism. In humans, there are three major isoforms, E2, E3, and E4. Compared to E3, E4 increases ACD and AD risk and vulnerability to the deleterious cognitive effects of a HFD. The plant compound Xanthohumol (XN) had beneficial effects on cognition and metabolism in C57BL/6J wild-type (WT) male mice put on a HFD at 9 weeks of age for 13 weeks. As the effects of XN in the context of a HFD in older WT, E3, and E4 female and male mice are not known, in the current study male and female WT, E3, and E4 mice were fed a HFD alone or a HFD containing 0.07% XN for 10 or 19 weeks, starting at 6 months of age, prior to the beginning of behavioral and cognitive testing. XN showed sex- and ApoE isoform-dependent effects on cognitive performance. XN-treated E4 and WT, but not E3, mice had higher glucose transporter protein levels in the hippocampus and cortex than HFD-treated mice. E3 and E4 mice had higher glucose transporter protein levels in the hippocampus and lower glucose transporter protein levels in the cortex than WT mice. In the standard experiment, regardless of XN treatment, E4 mice had nearly double as high ceramide and sphingomyelin levels than E3 mice and male mice had higher level of glycosylated ceramide than female mice. When the differential effects of HFD in E3 and E4 males were assessed, the arginine and proline metabolism pathway was affected. In the extended exposure experiment, in E3 males XN treatment affected the arginine and proline metabolism and the glycine, serine, and threonine metabolism. Myristic acid levels were decreased in XN-treated E3 males but not E3 females. These data support the therapeutic potential for XN to ameliorate HFD-induced cognitive impairments and highlight the importance of considering sex and ApoE isoform in determining who might most benefit from this dietary supplement.

## 1 Introduction

Obesity has been steadily increasing globally since the 1980s, with devastating consequences for health given obesity’s comorbidities with heart disease, stroke, and cancer, among other diseases (Hales et al., 2007; CDC, 2021). Fat consumption, specifically a diet high in saturated fatty acids (SFAs), has been shown to contribute substantially to the obesity epidemic (Freeman et al., 2013). Diet-induced obesity is a major risk factor for the development of cognitive impairment and dementia (Smith et al., 2011; Freeman et al., 2013). In the 1990s, evidence began to accumulate that a high fat diet (HFD), especially one high in saturated fat, could lead to cognitive impairments. In one of the first studies on the topic, Winocur and Greenwood (Greenwood and Winocur, 1990) showed that young rats fed a diet containing 40% calories from saturated fat diet had impaired spatial and working memory compared to rats fed a low fat diet, and even rats fed 40% calories from polyunsaturated fat diet (Greenwood and Winocur, 1990). They later showed that a diet containing as low as 20% calories from SFAs was sufficient to impair cognition in young rats (Greenwood and Winocur, 1996). Since then, a large body of work, has replicated these findings in both rodents and humans (Winocur and Greenwood, 2005; Smith et al., 2011; Freeman et al., 2013).

The *APOE* gene encodes three major isoforms of apolipoprotein E (ApoE) in humans, E2, E3, and E4. Compared to E3, E4 increases the risk to develop ACD and AD, particularly in women (Farrer et al., 1997; Raber et al., 2004). ApoE is involved in lipid metabolism and distribution, specifically sterol transport (Bandaru et al., 2009). Dysfunctional sterol and sphingolipid metabolism is found in the brains of people with AD, along with elevated levels of cholesterol and ceramide (Bandaru et al., 2009; Dinkins et al., 2016). In a mouse model of AD, ceramides co-localize with plaques in the hippocampus and amygdala (Kaya et al., 2017). Additionally, sphingomyelinase-2 knockout 5XFAD mice show less ceramide generation, and this is associated with attenuated AD pathology as well as improved cognition in these mice, thus pointing to this sphingolipid pathway as a potential therapeutic target for AD (Dinkins et al., 2016).

ApoE isoform might modulate the detrimental effects of a HFD on cognitive performance in an isoform-dependent fashion. Whereas a HFD impairs peripheral metabolism and cognition in both E3 and E4 female mice, spatial memory is significantly more impaired in E4 than E3 female mice on a HFD (Johnson et al., 2017). Whether a HFD has apoE isoform-dependent effects on cognitive performance in male mice is not known.

Xanthohumol (XN) is a prenylated flavonoid found in the hop plant, *Humulus lupulus* (Stevens and Page, 2004). XN has broad biological activity including chemopreventive as well as antioxidant activities *in vitro* (Stevens and Page, 2004; Jiang et al., 2018). XN has also been used to improve metabolic outcomes including total body weight, fasting glucose, and plasma triglyceride levels in a male rodent model of obesity and metabolic syndrome (Nozawa, 2005; Legette et al., 2013; Miranda et al., 2018; Zhang et al., 2020). The beneficial effects of XN in response to a HFD were also seen when XN did not significantly decrease body weight gain (Miranda et al., 2018). XN significantly decreased plasma markers of reactive oxygen species, decreased peripheral markers of dysfunctional lipid oxidation, as well as increased uncoupled cellular respiration in obese male rats, all of which could be mechanisms involved in the beneficial effects of XN on metabolic outcomes (Kirkwood et al., 2013a). XN may also have beneficial effects on cholesterol regulation. XN is an agonist for the farnesoid X receptor (FXR), which is involved in gluconeogenesis, *de novo* lipogenesis, and in the metabolism of cholesterol and bile acids (Nozawa, 2005; Sonne, 2021). XN may also directly impact adipose cells by inhibiting differentiation and proliferation and increasing apoptosis *in vitro* (Mendes et al., 2008; Rayalam et al., 2009; Legette et al., 2013). XN also impacts neural health. In a rodent model of cerebral artery occlusion, XN treatment decreased infarct size compared to control rats (Yen et al., 2012). Dietary supplementation with XN improves spatial as well as reversal learning in a Morris water maze, in young male mice (Zamzow et al., 2014; Miranda et al., 2018). Whether XN has beneficial effects on HFD-induced cognitive impairments in female mice is not known.

Bile acid (BA) signaling in the brain, while not as well characterized as BA signaling in the gastrointestinal tract, is gaining traction as a factor affecting cognition and brain health. A growing number of studies support BA signaling in the brain as a potential therapeutic target (McMillin and DeMorrow, 2016). BAs have been implicated to have roles in neurotransmission, neuroendocrine regulation of the HPA axis, as well as neurogenesis (McNeilly et al., 2010; Theofilopoulos et al., 2013; McMillin and DeMorrow, 2016). XN alters bile acid composition in mice, possibly mediating beneficial effects of XN on obesity, metabolism, and cognition (Zhang et al., 2020).

Ceramides, an intermediate of sphingolipid metabolism, may serve as molecular target for prevention of saturated fat-induced insulin resistance (Holland et al., 2007). Ceramide and its derivates inhibit glucose uptake and storage and induce oxidative stress, which are thought to be involved in the effects of ceramides on the development of insulin resistance (Summers and Nelson, 2005). Obesity as well as the consumption of high levels of SFAs have been linked to increased peripheral ceramide levels (Merrill, 2002; Holland et al., 2007). Ceramides can cross the blood brain barrier and contribute to HFD-induced cognitive impairments (Lyn-Cook et al., 2009; De La Monte et al., 2010). Because elevated ceramide levels have been implicated in the cognitive symptoms of AD (Xing et al., 2016), the ceramide lowering effects of XN make it a potential compound of therapeutic interest in the treatment AD as well as HFD-induced cognitive impairments.

Glucose is the major fuel source in the CNS and is transported across cell membranes *via* facilitated transport, thus along the glucose concentration gradient not against it, using glucose transporters (Duelli and Kuschinsky, 2001). The main glucose transporters in the brain are GLUT1 and GLUT3, with GLUT3 having 5-fold higher abundance (Duelli and Kuschinsky, 2001; Jurcovicova, 2014). GLUT1 transports glucose across the blood-brain-barrier as well as into axons for later conversion into lactate (Jurcovicova, 2014). GLUT3 is present primarily in axons and dendrites and is responsible for most glucose transport into neurons (Jurcovicova, 2014; Simmons, 2017). Protein levels of GLUT3 correspond to cerebral glucose metabolism (Wu et al., 2018). GLUT4 is an insulin-dependent glucose transporter found throughout the brain and may serve to supply neurons with additional glucose in conditions of high demand (Alquier et al., 2006; Jurcovicova, 2014). The density of glucose transporters is tightly regulated in the brain and changes based on local energy demand as well as various other factors (Duelli and Kuschinsky, 2001; Koepsell, 2020). GLUT1, and more dramatically GLUT3, are downregulated in the cerebral cortex of AD patients compared to age-matched healthy controls, and correspond to the reduced brain glucose-uptake and metabolism in that group (Simpson et al., 1994). This decrease in metabolizable energy could be a driving factor in the pathogenesis of AD (Winkler et al., 2015; Koepsell, 2020). Early reductions in brain GLUT1 occur long before onset of cognitive symptoms in AD (Winkler et al., 2015). In rodents, human ApoE isoform alters GLUT expression, with E4 mice showing impaired profiles of GLUT expression in the brain compared to E2 mice (Keeney et al., 2015). HFD feeding in rodents transiently downregulates GLUT1, GLUT3, and GLUT4, as well as reduce cerebral glucose intake (Jais et al., 2016; Kothari et al., 2017; Xi et al., 2019). Thus, one of the mechanisms of HFD-induced cognitive impairments is thought to be the HFD-induced downregulation of glucose transporters, resulting in less available glucose for neurons, even while the periphery is flooded with glucose to the point of insulin resistance (Jais et al., 2016). Given our previous findings on the beneficial effects of XN on metabolism and cognition in the context of a HFD in male mice, we hypothesized that XN might favorably alter glucose transporter expression in the brain. We also hypothesized that E4 mice would be the most adversely affected by the HFD and would show the most impaired GLUT expression profile in the cortex and hippocampus.

Previous studies investigating the effects of XN involved young (2–3 month old) wild-type (WT) male mice. In the present study, we determined the effects of XN on HFD-induced behavioral alterations and cognitive performance and metabolic pathways in the hippocampus in 6-month-old wild-type, E3, and E4, female and male mice and whether these effects are associated with alterations in ceramide levels and bile acid signaling and hippocampal and cortical glucose transporter levels.

## 2 Materials and methods

### 2.1 Experimental animals and XN diet exposure

**FIGURE 1:**
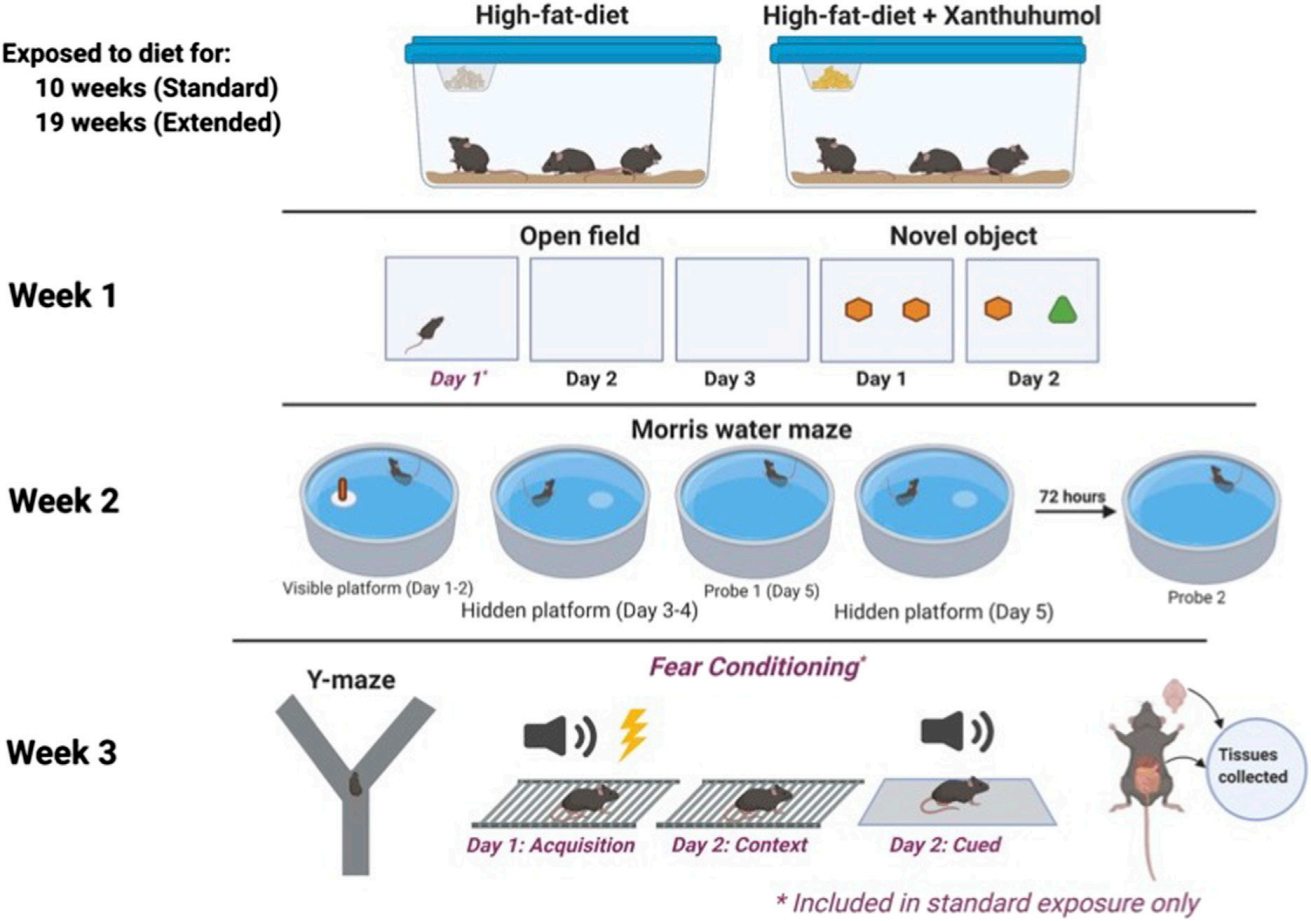
A timeline of the study.

Male and female C57BL/6J wild type mice (WT) as well as homozygous human E3 and E4 targeted replacement mice on a C57BL/6J background generated as described (Sullivan et al., 1997) were fed a HFD (60% kcal from fat, Dyets 103800, Bethlehem, PA) with 0 or 0.07% XN for 10 weeks (standard exposure) or 19 weeks (extended exposure) prior to the beginning of behavioral testing (Sullivan et al., 1997; Knouff et al., 1999). The percentage XN and preparation of the diet was the same as in our earlier studies. XN was first dissolved in OPT (oleic acid:propylene glycol:Tween80, 0.9:1:1 by weight) before it was incorporated into the HFD. Mice were started on the diets at approximately 6 months of age. Food and water were available *ad libitum.* Mice were weighed once weekly. Anticipated XN intake was approximately 60 mg/kg/day for XN treated animals (Miranda et al., 2016). Diets were continued through the 3 weeks of behavioral and cognitive testing. Mice were group housed until 5 days prior to the start of behavioral testing, at which point they were housed individually. Mice were on a 12:12 on–off light cycle (7:00 a.m.–7:00 p.m.). Testing of 100 mice was conducted in three cohorts of mice by an experimenter blind to both diet and genotype of the animals. A timeline of the study is shown in Figure 1. The order of the behavioral and cognitive tests was selected to start with the anticipated least stress-inducing test and end with the anticipated most stress-inducing test. All testing was performed by an experimenter blind to the genotype and treatment of the mice. Y-maze, open field, object recognition and water maze performance were tracked using video tracking software (Ethovision 7.0 XT, Noldus). The group sizes are represented in Table 1. All procedures complied with the NIH Guide for the Care and Use of Laboratory Animals and with IACUC approval at OHSU. At the end of the behavioral and cognitive testing, mice were euthanized, and the hippocampus and whole cortex collected.

**TABLE 1:** The number of mice in the standard and extended exposure experiments.

Genotype	HFD		HFD + XN	
	Females	Males	Females	Males
Standard exposure				
WT	10	9	10	9
E3	8	9	7	7
E4	8	8	8	7
Extended exposure				
E3	8	8	7	9

### 2.2 Behavioral and cognitive analyses

#### 2.2.1 Spontaneous alternation in the Y-maze

Hippocampus-dependent spontaneous alterations were assessed in a Y-shaped maze (O’ Hara & Co., Ltd, Tokyo, Japan) consisting of raised sides (3.8 cm bottom width, 12.55 cm top width, 12.55 cm height) with plastic, opaque grey arms (37.98 cm length) at a 120° angle from each other. Mice were placed into the center of the maze at the beginning of each 5-min trial. The mazes were cleaned with 0.5% acetic acid between trials. The mazes were surrounded with a white curtain in order to isolate the mice from the surrounding room as well as the experimenter. Spontaneous alternations and activity levels were analyzed in the Y-maze. Videos were analyzed to measure the number of arm entries and to calculate the percent spontaneous alternations. The criteria for an arm entry was when all four limbs were within the arm. The spontaneous alternation percentage was calculated by dividing the number of 3-arm alternations by the number of possible 3-arm alternations and multiplying the value by 100 (Prieur and Jadavji, 2019).

#### 2.2.2 Performance in the open field and object recognition

Open field and object recognition was conducted as previously described (Raber et al., 2019). Exploratory activity and measures of anxiety were assessed using the open field test. The arena consisted of a well-lit square (L 40.6 × W 40.6 × H 40.6 cm) with a central light intensity of 100 lux. Mice were allowed to explore the open field for 5 min. Open field testing was conducted on three consecutive days for the standard exposure experiment and for two consecutive days for the extended exposure experiment. The next day, mice were exposed to the open field containing two identical objects (orange wooden blocks in the shape of hexagonal prisms) for a 15-min trial. The objects were placed 10 cm apart and 15 cm from the adjacent walls of the arena. The following day, one object was replaced with a novel object (green wooden block in the shape of a triangular prism) and mice were allowed to explore for 15 min. During object recognition trials, objects were affixed to the floor of the arena using masking tape. The arenas and objects were cleaned with 0.5% acetic acid between trials. Physical interaction with the object in the form of sniffing within a 2 cm proximity was coded as object exploration. A discrimination index was calculated as previously described (Lueptow, 2017). The time spent exploring the familiar object was subtracted from the time exploring the novel object, and the resulting number was divided by the total time spent exploring both objects.

#### 2.2.3 Spatial learning and memory in the water maze

The water maze task was conducted in a circular pool (diameter 140 cm), filled with water (19°C) as previously described (Johnson et al., 2016). White chalk was added to the water to make it opaque. The maze was divided conceptually into four quadrants. Four extra-maze cues (50 × 50 cm) of various shapes and colors were placed around the maze at the borders of the four quadrants. A white curtain surrounded the maze to isolate the maze and extra-maze cues from the surrounding room, as well as the experimenter. Mice were tested for 2 sessions per day (separated by 3 h), with each session consisting of two trials (separated by 10 min). Testing was conducted over the course of 6 days (2 days of visible platform, followed by 3 days of hidden platform with a probe trial prior to the last day of hidden platform trials, followed by a probe trial 72 h later). Mice were first trained to locate a submerged circular Plexiglas® platform (diameter 12 cm) 2 cm below the surface of the water. During the visible trials, the platform was marked by the use of a cue (a multi-colored cylinder, diameter 5 cm, height 8 cm). Once mice found the platform and remained on it for 3 s, they were removed from the pool and returned to their home cage. Mice were placed into the maze from various starting locations at the beginning of each trial and the platform was moved during each session of visible trials to avoid procedural bias. Following the 4 sessions of visible platform training, the cue was removed and hidden platform trials began. During hidden platform trials, the platform remained in one location (southwest quadrant). Spatial memory retention was assessed in a probe trial before hidden platform trials on day 5, as well as 72 h after the conclusion of hidden platform training trials. During probe trials, the platform was removed from the pool and mice remained in the pool for the full 60-s trial.

#### 2.2.4 Fear learning and contextual and cued fear memory

Fear conditioning was assessed over the course of 2 consecutive days using a Med Associates mouse fear conditioning system (PMED-VFC-NIR-M, Med Associates, St. Albans, Vermont) and Med Associates VideoFreeze automated scoring system, as described (Weiss et al., 2017). Mice were placed inside the fear conditioning chamber, where chamber lights were turned on at the beginning of the trial. The first day of testing consisted of the acquisition phase, which lasted a total of 10 min per trial. Following a 120 s baseline habituation period, 4 30-s tones (80 dB) were presented separated by inter-stimulus-intervals (ISIs). Simultaneous with the last 2 s of each tone, a 0.35 mA foot shock was administered. Twenty-four hours later the hippocampus-dependent contextual fear memory was assessed. Mice were placed into the same chamber as the acquisition phase and remained there for a 5-min trial. The chamber lights were on, but no tones or shocks were present during this phase of testing. Two hours after the context phase of testing, hippocampus-independent cued fear memory was assessed. Mice were placed into the same chamber, which was modified with a novel floor texture, angled walls, as well as a novel vanilla scent. Each cued trial lasted a total of 6 min. A 180 s baseline period was followed by 180 s of a tone. No shocks were administered during this phase of testing. Between trials, the chambers were thoroughly cleaned with 0.5% acetic acid (acquisition and context phases) or 10% isopropanol (cued phase). Fear conditioning was conducted for the standard exposure experiment, but not the extended exposure experiment.

### 2.3 Quantification of glucose transporter protein levels in the hippocampus and cortex

Protein levels of glucose transporters 1, 3, and 4 (GLUT1, GLUT3, GLUT4, respectively) were quantified from lysates of tissue from the hippocampus and cortex (*n* = 6–10 per sex per group). Tissue was processed in ice cold buffer containing 1M Tris-Cl, 6M NaCl, 0.5M EDTA, and Triton-X-100. Protein concentrations were determined using a BCA protein assay (Thermo Fisher, Waltham, MA). Levels of GLUT1, GLUT3, and GLUT4 were analyzed with an ELISA-based quantification assay (MyBioSource, San Diego, CA) according to the manufacturer’s instructions. Briefly, samples were diluted so that glucose transporter protein levels fell within the range of the standard curve. Samples were then transferred to the 96-well ELISA plate. At the end of the assay, absorbances were read at 450 nm using an ID5reader (Molecular Devices, San Jose, CA). Glucose transporters were quantified for standard exposure mice only.

### 2.4 Hippocampal metabolomics analysis

Hippocampi from 45 E3 and E4 mice (E3 HFD females: *n* = 6; E3 HFD + XN females: *n* = 6; E3 HFD males: *n* = 6; E3 HFD + XN males: *n* = 6; E4 HFD females: *n* = 5; E4 HFD + XN females: *n* = 6; E4 HFD males: *n* = 5; E4 HFD + XN males: *n* = 6) part of the standard exposure experiment and from 25 E3 (E3 HFD females: *n* = 8; E3 HFD + XN females: *n* = 7; E3 HFD males: *n* = 5; E3 HFD + XN males: *n* = 5 mice part of the extended exposure experiment) were processed for metabolomics. The hippocampal tissues were homogenized in 300 μL (per 30 mg of tissues) of cold methanol: water (8:2, v/v). From 100 μl of hippocampal homogenates, metabolites were extracted. Untargeted metabolomics was performed as described (Kirkwood et al., 2013b). Liquid chromatography (LC) was performed using a Shimadzu Nexera system with an Insertil Phenyl-3 column (4.6 × 150 mm, 100 Å, 5 μm; GL Sciences, Rolling Hills Estates, CA, United States) coupled to a quadrupole time-of-flight mass spectrometer (Q-TOF) (AB SCIEX, Triple TOF 5600) operated in information-dependent MS/MS acquisition mode. Samples were ordered randomly and multiple quality control samples were included. QC samples were generated by pooling 10 µL aliquots from hippocampal extracts and analyzed along with the samples. Samples were run in the positive and negative ion mode. In case metabolites were present in both ion modes, the mode with the higher peak value was selected for further analysis. The column temperature was held at 50°C and the samples were kept at 10°C. For analysis, raw data were used and no log transformation or Pareto scaling was used. The metabolomics data were processed using Markerview (SCIEX, Framingham, MA) and Peakview (SCIEX, Framingham, MA) software for peak detection, alignment, identity assignment, data visualization, and statistical analysis. Identification of metabolites was based on mass error (<30 ppm) and MS/MS fragment ions. Metabolites were also confirmed using retention time, mass-to-charge (m/z) ratio, and comparison to authentic standards (±1 min) from an in-house library (IROA Technologies, Bolton, MA), allowing for the streamlined identification of metabolites. LipidMaps (Welcome Trus, United Kingdom), METLIN (Scripps, La Jolla, CA) and HMDB (University of Alberta, Edmonton, Canada) databases were used for MS and MS/MS matching. MetaboAnalyst pathway analysis (version 5.0, Montreal, Quebec, Canada) was performed as described by (Xia and Wishart, 2010; Kirkwood et al., 2013b). Raw metabolite peak values in the hippocampus were analyzed, without log transformation or Pareto scaling.

### 2.5 Statistical analysis

Data were analyzed using SPSS Statistics for Windows (Version 25, Armonk, NY: IBM Corp.) and SAS (Version 9.4, Cary, NC: SAS Institute Inc.). Functional behavioral/lipidomic/metabolomics/GLUT transporter data were analyzed using ANOVA or repeated measures ANOVA. The fixed effects in the standard-exposure experiment were dietary treatment (HFD or XN), genotype (WT, E3, or E4), sex (females or males), and their two-way interactions. In addition, we examined each genotype separately. Since the extended-exposure experiment was done using only E3 mice, we examined the effect of exposure length using only E3 mice in the standard- and extended-exposure experiment. The fixed effects in the model were treatment (HFD or XN), sex (females or males), treatment exposure length (standard- or extended exposure), and their two-way interactions. Given the complex experimental design, we also examined the treatment effect for each genotype x sex x treatment exposure separately for a total of 8 comparisons (6 comparisons for the standard exposure experiment; 2 comparisons for the extended exposure experiment). Main effects of treatment were determined using a sign test by calculating the number of positive and negative differences across the 8 genotype x sex x treatment exposure length combinations. Statistical significance was determined using an error probability level of *p* < 0.05.

Correlations between functional behavioral and lipidomic/metabolomics/GLUT transporter measures were calculated using the non-parametric Spearman rank test. To account for multiple comparisons and the false discovery rate, we report only on lipidomic/metabolomics/GLUT transporter measures that had at least 10% of functional behavioral measures significantly correlated at *p* < 0.05.

For metabolomic pathway analysis, four distinct comparative metabolomics analyses were performed as part of the standard-exposure length experiment: 1) effects of XN in E3 mice; 2) effects of XN in E4 mice; 3) differential effects of HFD in E3 and E4 mice; and 4) differential effects of HFD + XN in E3 and E4 mice. One comparative analysis was performed in the extended-exposure length experiment: effect of XN in E3 mice. Pathways were visualized using scatter plots (testing significant features) in MetaboAnalyst, with the “global test” and “relative-betweenness centrality” as parameters for enrichment method and topological analysis, respectively. MetaboAnalyst software (version 5.0) was used to generate impact plots. Statistical significances were adjusted for multiple comparisons.

Data are expressed as mean ± SEM. Graphs were generated using GraphPad software v.8.2.0 (La Jolla, CA, United States).

## 3 Results

### 3.1 No effects of XN on body weights

Over the course of the study, mice gained weight on the HFD in both the standard and extended exposure experiments (Repeated measures ANOVA, effect of week, *p <* 0.001). In both the standard and extended exposures, there was an effect of sex on body weight in all genotypes (standard exposure: *p <* 0.001 for all genotypes; extended exposure: *F*[1,21] = 9.026, *p* = 0.007), with lower body weights in females than males (Supplementary Figures S1A–C). In the extended exposure experiment, while body weights of females were overall significantly lower than those of males, in the final few weeks, body weights of female and male mice were similar (Supplementary Figure S1D). In both experiments, there were no effects of treatment on body weights.

### 3.2 Genotype-dependent effects of XN on spontaneous alterations and activity in the Y-maze

**FIGURE 2:**
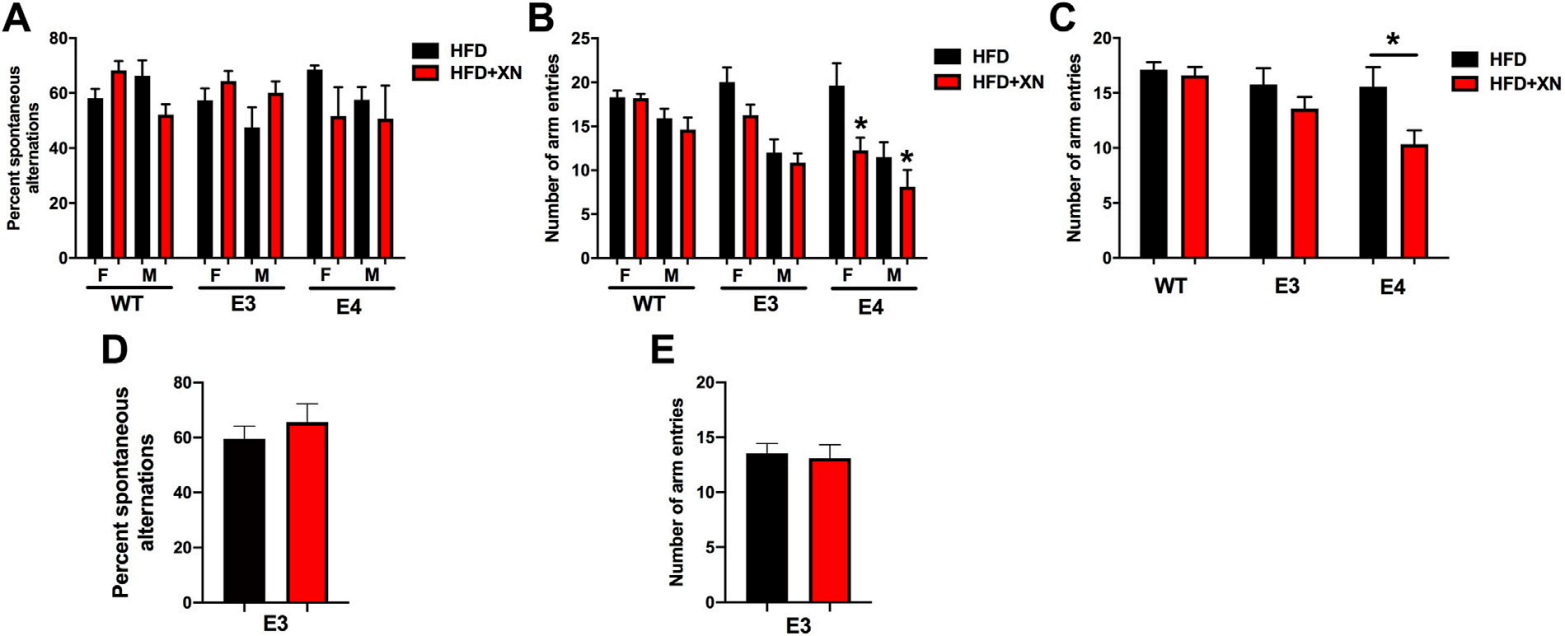
Genotype-dependent effects of XN on spontaneous alterations and activity in the Y-maze. **(A)**. Hippocampus-dependent spontaneous alternation in the standard exposure experiment. **(B)**. The number of arm entries in the Y-maze in the standard exposure experiment. In E4 mice, there was an effect of XN on activity levels. XN-treated E4 mice entered less arms than sex-matched E4 mice only on a HFD. Overall effect of XN: *p = 0.0004. **(C)**. The number of arm entries in the Y-maze with sexes combined. XN-treated E4 mice entered less arms than sex-matched E4 mice only on a HFD. *p = 0.0004. **(D)**. The percent of spontaneous alternations of E3 mice in the Y-maze with sexes combined in the extended exposure experiment. There was no effect of XN on spontaneous alternation of E3 mice in the extended exposure experiment. **(E)**. The number of arm entries of E3 mice in the extended exposure experiment in the Y-maze with sexes combined. There was no effect of XN on arm entries of E3 mice. All graphs show the mean ± SEM.

The results for the Y-maze performance in the standard and extended exposure experiment are shown in Figure 2. To evaluate hippocampus-dependent spatial function, the percent spontaneous alternation in the Y-maze was analyzed. To assess activity levels in the Y-maze, the number of arm entries was analyzed. There was a treatment by genotype interaction for percent spontaneous alternation (*p* = 0.048) and for number of arm entries (*p* = 0.03). In E4 mice, XN treatment decreased the number of arm entries (*p* = 0.0004) and the percent spontaneous alternation to the point of chance (*p* = 0.05). In E3 mice, XN treatment increased the spontaneous alternation in the standard and extended exposure experiment above chance in all 4 treatment x sex x exposure length comparisons, whereas the number of arm entries were not altered by XN treatment. In WT mice, XN treatment did not overall affect the percent spontaneous alternation or the number of arm entries (both *p* = 0.99). XN increased spontaneous alternation in females but decreased it in males.

There was also an effect of sex. Females alternated more often than males (7 of 8 comparisons) and were more active than males; in both experiments, females entered more arms (8 of 8 treatment x sex x genotype x exposure length comparisons; *p* < 0.0001).

### 3.3 Genotype-dependent effects of XN on exploratory behavior in the open field

Behavioral performance in the open field test in the standard (A, B) and extended (D, E) exposure experiment are shown in Supplementary Figure S2. To assess activity levels in the open field, the distance moved was analyzed. Mice habituated to the open field; distance moved decreased on subsequent days (*p* < 0.001). To evaluate anxiety levels, the time in the center was analyzed. In WT and E4 mice, XN-treated mice show lower anxiety levels than vehicle-treated mice, as XN decreased the time spent in the center (12 of 16 combinations; *p* interaction = 0.003), whereas the opposite pattern was seen in E3 mice in the standard exposure experiment. There were also sex x experiment interactions: female mice were more active in the standard than the extended exposure experiment or male mice in either experiment (*p* < 0.0001). In addition, male mice spent more time in the center in the extended than standard exposure experiment or female mice in either experiment (*p* = 0.0002).

### 3.4 XN-treated mice spent more time with novel object

The results for the novel object recognition test in the standard (C) and extended (F) exposure experiment are also shown in Supplementary Figure S2. The discrimination index was analyzed to assess object recognition. XN-treated mice preferred a novel over a familiar object (in all 6 standard exposure comparisons; *p* = 0.03), whereas HFD only-treated mice spent a similar amount of time exploring both objects.

### 3.5 Genotype-dependent effects of XN on learning during the visible platform water maze trials

**FIGURE 3:**
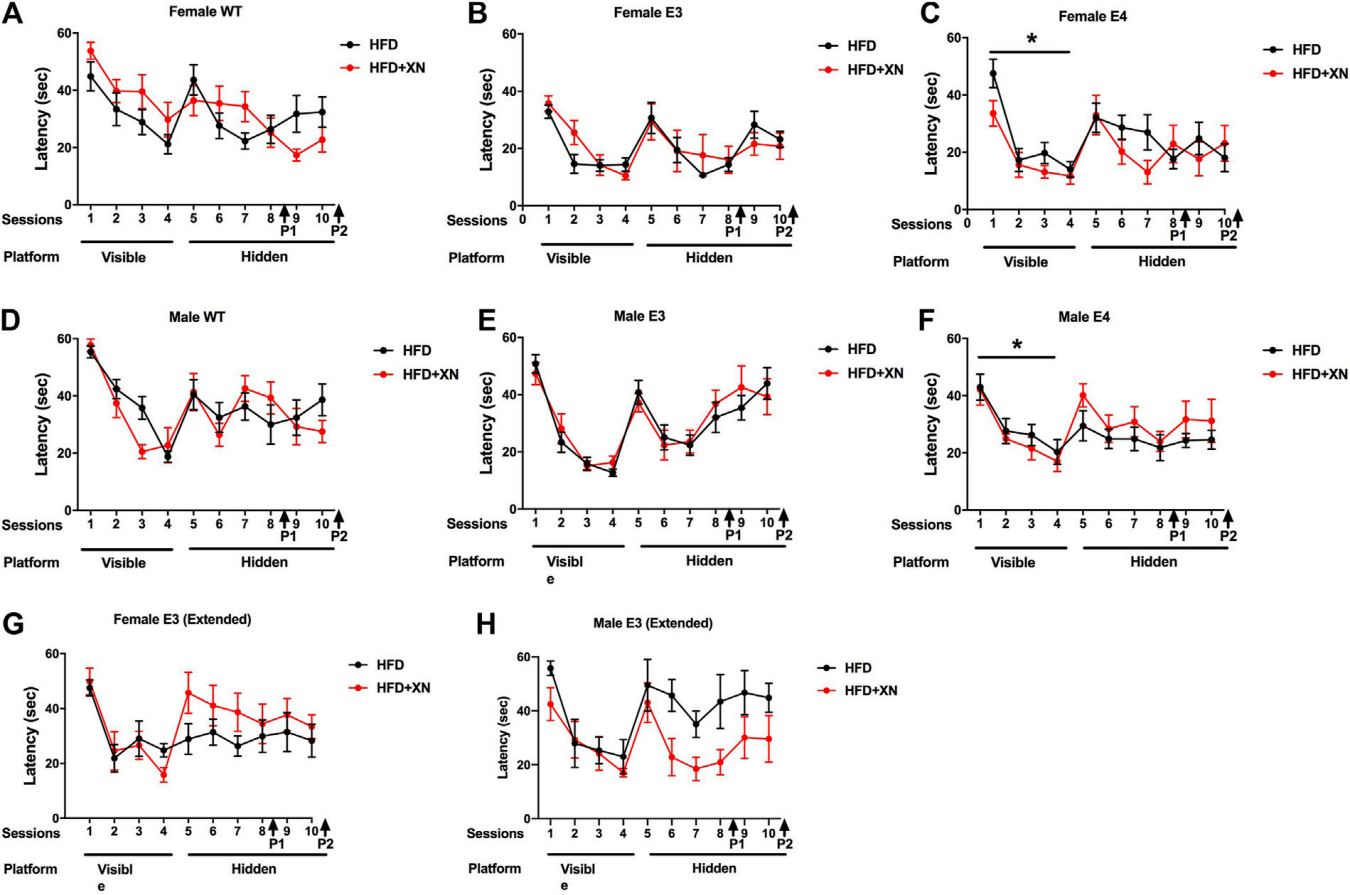
Spatial learning and memory of female WT **(A)**, E3 **(B)**, E4 **(C)** and male WT **(D)**, E3 **(E)**, and E4 **(F)** in the water maze in the standard exposure experiment. The latency to locate the platform is shown. Mice were first trained in 4 sessions in which the platform location was visible, and subsequently they were trained to locate a hidden platform using extra-maze cues. In E4 mice, XN treatment improved performance during the visible platform training. *p < 0.05. (**G,H)** Spatial learning and memory of female **(G)** and male **(H)** E3 mice during the extended exposure experiment. There was a treatment by sex interaction during hidden platform training. XN improved performance of E3 males only **(H)**. *p = 0.029. There was no significant effect of treatment on performance of females during the hidden platform training **(G)**.

The results for the visible platform water maze trials in the standard (A-F) and extended (G, H) exposure experiment are shown in Figure 3. As swim speed can affect performance in the water maze, we first assessed swim speeds during training of the mice to locate the visible platform (Supplementary Figure S3). Swim speed was similar in the standard and extended exposure experiment and did not change over subsequent trials. WT and E3 mice swam faster than E4 mice (*p* < 0.004), except for day 3, when WT and E3 mice were swimming unusually slow. Females swam faster than males in the standard exposure experiment (*p* < 0.0001).

Mice learned quickly to navigate to the visual platform of the water maze, as they reached the platform quicker in subsequent trials, with the largest improvement occurring from day 1 to day 2, as 72% and 93% of mice reached the visible platform on day 1 and 2, respectively (*p* < 0.001; Figure 3). Despite their slower swim speeds, E4 mice reached the platform quicker than WT mice. In the last visible platform trial, WT mice required more time to reach the visible platform than other two genotypes. Despite the slower swim speeds, males caught up with females in performance starting on day 3 of the visible platform water maze trials and reached the platform as quick as females with a similar cumulative distance to the target.

There also was a genotype × treatment interaction: XN-treatment improved performance in E4 mice across all four visible platform trials, whereas the opposite pattern was seen in WT mice. In E3 mice, the XN treatment promoted learning, as XN-treated mice had a superior performance over HFD only-treated mice in the last two visible platform trials. Overall, performance deteriorated with age, HFD, and/or XN treatment, as time to reach the visible platform was lower in the standard than the extended exposure experiment.

### 3.6 XN improved learning in genotype-sex groups that did not perform well during the hidden platform water maze trials

The results for the hidden platform water maze trials in the standard and extended exposure experiment are shown in Figure 3. Similarly to the visible platform water maze trials, mice learned how to reach the hidden platform faster with training, with the largest improvements occurring from day 1 to day 2 with 78% and 90% of mice reaching the hidden platform on day 1 and 2 (*p* < 0.001; Figure 3). E3 and E4 mice learned faster than WT mice, as the cumulative distance to the platform and time to reach the platform were greater for WT mice than E3 or E4 mice on the first 4 days of the hidden platform trials; in E4 mice, this pattern persisted to the last 2 days. Females learned faster than males, as the cumulative distance to the platform and time to reach the platform were greater in males than females on the last 4 days of the hidden platform trials.

Beneficial effects of XN were observed in genotype and sex groups that did not do well on only the HFD. XN improved performance of XN-treated vs. HFD only-treated male mice in the extended exposure experiment. In WT mice, XN decreased both the cumulative distance to the hidden platform and latency in the last two training days, because XN-treated mice continued to improve whereas the performance of HFD only-treated WT mice plateaued. Overall, performance deteriorated with age, HFD, and/or XN treatment, as cumulative distance to the platform and time to reach the hidden platform were smaller in the standard than the extended exposure experiment.

### 3.7 XN improved locating the platform during the water maze probe trials

**FIGURE 4:**
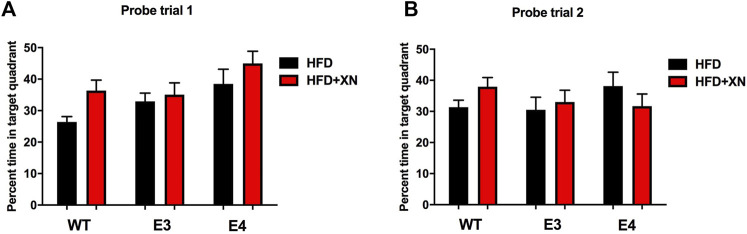
Spatial memory retention in the first **(A)** and second **(B)** probe trials. Between sessions 8 and 9 of the water maze, and after session 10, the platform was removed from the pool during probe trials. Mice spending more time in the part of the pool where the platform was located previously (target quadrant) reflects better performance. XN-treated WT mice spent more time in the target quadrant than WT mice only on a HFD. *p < 0.05. All graphs show the mean ± SEM.

The results for spatial memory retention in the water maze probe trials in the standard exposure experiment are shown in Figure 4. XN-treated mice spent more time in the target quadrant reached and in 5 of 6 genotype x sex comparisons had a lower cumulative distance to the platform location than HFD only-treated mice. In all comparisons, female mice spent more time in the target quadrant and had a lower cumulative distance to the platform location than male mice. In the first, but not in the second probe trial, E4 mice spent more time in the target quadrant and showed a smaller cumulative distance to the platform location than WT mice. In the extended exposure experiment, there was a sex × treatment interaction. XN treatment improved the performance of the E3 males but not of E3 females (Supplementary Figure S4).

### 3.8 Genotype-dependent effect of XN on baseline activity levels in the fear conditioning test

The fear conditioning test was only done in the standard exposure experiment; the results are shown in Supplementary Figure S5. During the baseline period in the fear conditioning test (prior to the first tone and shock), WT mice moved more than E3 or E4 mice. Except for WT female mice, HFD only-treated mice moved more than XN-treated mice and female mice moved more than male mice (Supplementary Figure S5A). The fear response increased with each shock, as the mice froze more and 36%, 77%, 86%, and 90% of mice froze during the first, second, third, and fourth tones, respectively (Supplementary Figure S5C). WT male mice were least likely to freeze, as 44% did not freeze during the fourth tone, whereas all E3 or E4 mice froze during the fourth tone. Female mice froze longer than male mice. Except for E3 mice, freezing during the tone increased more in XN-treated than HFD only-treated mice.

Freezing between the tone-shock intervals increased with each interval (Supplementary Figure S5D). The greatest increases were observed in E4 mice, intermediate increases in E3 mice and least increases in WT mice. Contextual fear memory was greater in XN-treated mice than HFD only-treated mice and weaker in WT than non-WT mice (Supplementary Figure S5E). Cued fear memory was greater in E4 mice than in E3 mice and in E3 mice than in WT mice, with E4 mice showing more freezing during the pre-tone period (Supplementary Figure S5F). Males showed higher freezing levels during the pre-tone period but cued fear memory did not differ among sexes. XN-treated mice showed more freezing during the pre-tone period than HFD only-treated mice but there was no difference in cued fear memory. E4 had the strongest cued fear time, E3 mice had intermediate cued fear memory, and WT had the weakest cued fear memory.

### 3.9 Effects of XN on glucose transporter protein levels in the hippocampus and cortex

**FIGURE 5:**
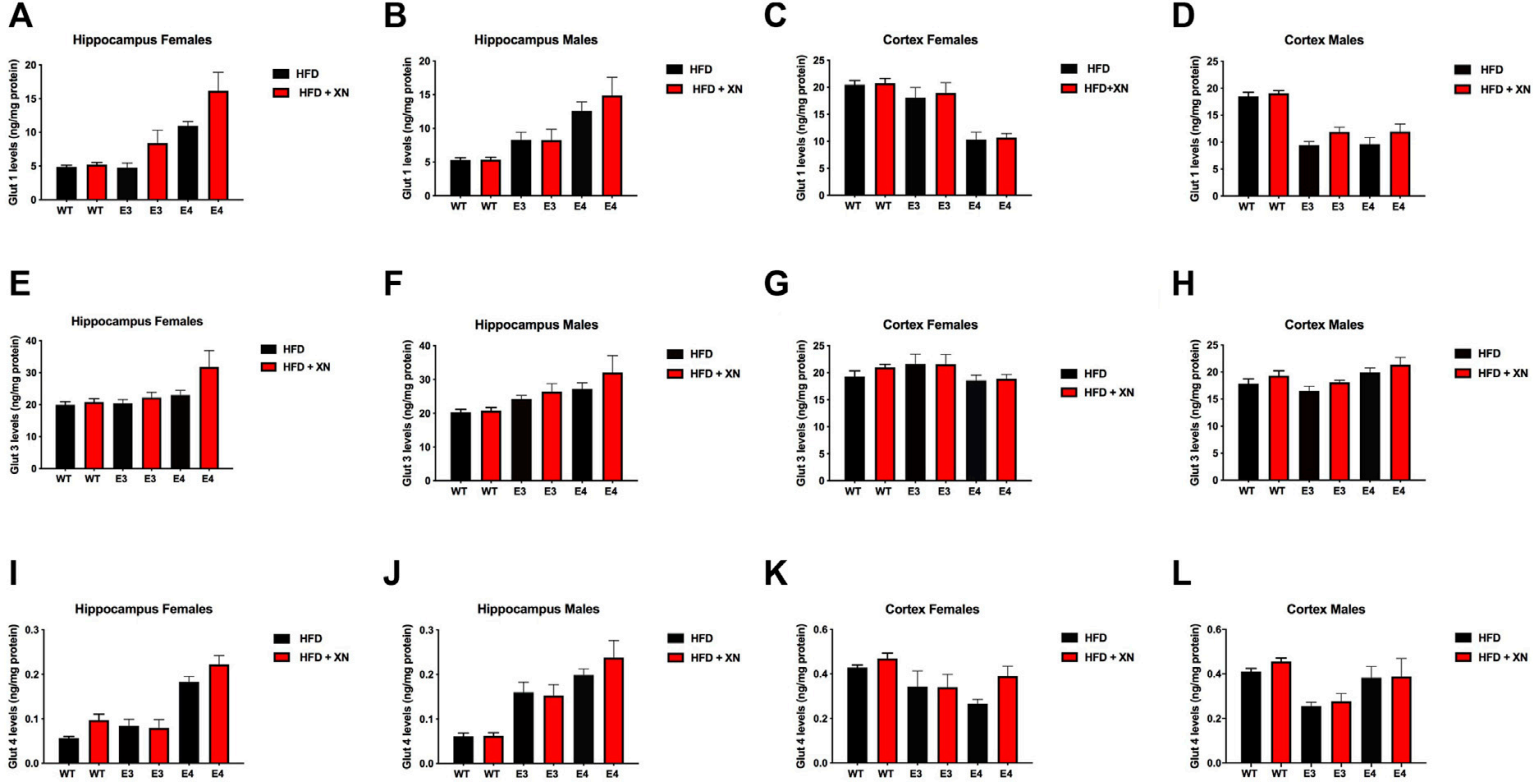
Protein levels of GLUT1 **(A–D)**, GLUT3 **(E–H)**, and GLUT4 (**I–L**) glucose transporters in the hippocampus **(A,B,E,F,I,J)** and cortex (**C,D,G,H,K,L**). All graphs show the mean ± SEM. XN-treated mice had higher glucose transporter protein levels in the hippocampus and cortex than HFD only-treated mice, with the exception of E3 mice. Among the 12 sex-genotype-site combinations for each of the 3 glucose transporters, XN-treated mice had for 31 of 36 comparisons higher levels than HFD only-treated mice (sign test: *p* < 0.0001). In all WT and E4 mice comparisons, XN-treated mice had higher levels, whereas in 5 of 12 E3 mice comparisons, HFD only-treated mice had higher levels. The XN-effect was significant for Glut1 (*p* = 0.01) and Glut3 (*p* = 0.02) in the hippocampus and tended to be significant for Glut 4 (*p* = 0.075) in the hippocampus and for Glut 3 (*p* = 0.095) and Glut 4 (*p* = 0.08) in the cortex. E4 mice had higher glucose transporter protein levels in the hippocampus and lower glucose transporter protein levels cortex than WT mice. In all 4 sex-treatment comparisons for each of the 3 glucose transporters, E4 mice had higher glucose transporter protein levels in the hippocampus (sign test: *p* = 0.0005), and in 10 of 12 comparisons, E4 mice had lower glucose transporter protein levels in the cortex (*p* = 0.04) than WT mice. E3 mice also had higher glucose transporter protein levels in the hippocampus and lower glucose transporter protein levels cortex than WT mice. In 10 of 12 comparisons, E4 mice had higher glucose transporter protein levels in the hippocampus (sign test: *p* = 0.04) and lower glucose transporter protein levels in the cortex (sign test: *p* = 0.04) than WT mice. Female and male E4 mice had higher glucose transporter protein levels in the hippocampus and female E4 mice had lower glucose transporter protein levels in the cortex than female E3 mice in all 18 comparisons (sign test: *p* < 0.0001). The exception were cortical levels in male E4 mice, which were higher in 5 of 6 E4 vs. E3 comparisons (Fisher’s exact test: *p* = 0.0007). Male mice had higher glucose transporter protein levels in the hippocampus than female mice in 14 of 18 comparisons (*p* = 0.03) and lower glucose transporter protein levels in the cortex than female mice in all 12 WT and E3 comparisons (sign test: *p* = 0.0005). The exception were glucose transporter protein levels in the cortex of male E4 mice, which were higher in 5 of 6 E4 comparisons with female E4 mice (Fisher’s exact test: *p* = 0.0007).

XN-treated mice had higher glucose transporter protein levels in the hippocampus and cortex than HFD only-treated mice, with the exception of E3 mice (Figure 5; Table 2). Among the 12 sex-genotype-site combinations for each of the 3 glucose transporters, XN-treated mice had for 31 of 36 comparisons higher levels than HFD only-treated mice (sign test: *p* < 0.0001). In all WT and E4 mice comparisons, XN-treated mice had higher levels, whereas in 5 of 12 E3 mice comparisons, HFD only-treated mice had higher levels. Using ANOVA, the XN-effect was significant for Glut1 (*p* = 0.01) and Glut3 (*p* = 0.02) in the hippocampus and tended to be significant for Glut 4 (*p* = 0.075) in the hippocampus and for Glut 3 (*p* = 0.095) and Glut 4 (*p* = 0.08) in the cortex.

**TABLE 2:** Glucose transporter protein levels (in ng/mg protein) affected by XN in the hippocampus and cortex in the standard exposure experiment.

	Female					Male				
	HFD		XN		P-value	HFD		XN		P-value
Hippocampus	Wildtype									
Glut 1	4.87	0.25	5.23	0.30	0.83	5.30	0.35	5.36	0.35	0.97
Glut 3	19.89	0.99	20.76	1.09	0.77	20.31	0.82	20.74	0.90	0.89
Glut 4	0.056	0.004	0.097	0.014	0.07	0.061	0.008	0.062	0.007	0.95
	E3									
Glut 1	4.74	0.72	8.39	1.93	0.07	8.29	1.14	8.26	1.59	0.99
Glut 3	20.41	1.18	22.20	1.65	0.60	24.17	1.08	26.34	2.41	0.52
Glut 4	0.084	0.015	0.080	0.019	0.86	0.159	0.023	0.153	0.024	0.79
	E4									
Glut 1	**10.94**	**0.67**	**16.18**	**2.73**	**0.007**	12.57	1.35	14.89	2.69	0.27
Glut 3	**22.98**	**1.49**	**31.78**	**5.13**	**0.008**	27.18	1.80	32.02	5.02	0.18
Glut 4	0.183	0.012	0.222	0.020	0.11	0.199	0.014	0.238	0.038	0.16
Cortex	Wildtype									
Glut 1	20.50	0.77	20.79	0.85	0.84	18.47	0.76	19.04	0.52	0.70
Glut 3	19.28	1.04	20.96	0.57	0.22	17.85	0.90	19.31	0.90	0.31
Glut 4	0.43	0.01	0.47	0.02	0.41	0.41	0.01	0.46	0.01	0.38
	E3									
Glut 1	18.10	1.86	18.96	1.90	0.60	9.43	0.70	11.90	0.87	0.12
Glut 3	21.61	1.83	21.57	1.80	0.98	16.52	0.86	18.08	0.41	0.31
Glut 4	0.34	0.07	0.34	0.06	0.96	0.26	0.02	0.28	0.04	0.70
	E4									
Glut 1	10.37	1.40	10.45	0.79	0.96	10.43	1.31	11.21	1.28	0.65
Glut 3	18.55	1.01	18.71	0.86	0.92	19.90	0.85	21.34	1.36	0.40
Glut 4	**0.27**	**0.02**	**0.39**	**0.04**	**0.03**	0.38	0.05	0.39	0.08	0.93

Significant effects are indicated in bold.

E4 mice had higher glucose transporter protein levels in the hippocampus and lower glucose transporter protein levels cortex than WT mice. In all 4 sex-treatment comparisons for each of the 3 glucose transporters, E4 mice had higher glucose transporter protein levels in the hippocampus (sign test: *p* = 0.0005), and in 10 of 12 comparisons, E4 mice had lower glucose transporter protein levels in the cortex (*p* = 0.04) than WT mice. Similarly, E3 mice had higher glucose transporter protein levels in the hippocampus and lower glucose transporter protein levels cortex than WT mice. In 10 of 12 comparisons, E4 mice had higher glucose transporter protein levels in the hippocampus (sign test: *p* = 0.04) and lower glucose transporter protein levels in the cortex (sign test: *p* = 0.04) than WT mice. Female and male E4 mice had higher glucose transporter protein levels in the hippocampus and female E4 mice had lower glucose transporter protein levels in the cortex than female E3 mice in all 18 comparisons (sign test: *p* < 0.0001). The exception were cortical levels in male E4 mice, which were higher in 5 of 6 E4 vs. E3 comparisons (Fisher’s exact test: *p* = 0.0007).

Male mice had higher glucose transporter protein levels in the hippocampus than female mice in 14 of 18 comparisons (*p* = 0.03) and lower glucose transporter protein levels in the cortex than female mice in all 12 WT and E3 comparisons (sign test: *p* = 0.0005). The exception were glucose transporter protein levels in the cortex of male E4 mice, which were higher in 5 of 6 E4 comparisons with female E4 mice (Fisher’s exact test: *p* = 0.0007).

### 3.10 Correlations between behavioral functional measures and glucose transporter protein levels in the hippocampus and cortex

Several measures of behavioral performance were linked to glucose transporter protein levels (Supplementary Table 3). Stronger fear memory responses were linked to higher glucose transporter levels in the hippocampus and lower glucose transporter levels in the cortex. Freezing during the third and fourth tones of fear conditioning training and freezing between the tone-shock intervals and cued fear memory were positively correlated with glucose transporter levels in the hippocampus and negatively correlated with glucose transporter 1 levels in the cortex (Supplementary Table 3A).

In contrast to the fear conditioning test, activity levels in the open field were linked to lower glucose transporter levels in the hippocampus and higher glucose transporter levels in the cortex (Supplementary Table 3B). Using Spearman correlation coefficients, we observed in all three open field trials positive correlations between the distance moved in the open field test and glucose transporter protein levels in the cortex and negative correlations between the distance moved and glucose transporter protein levels in the hippocampus (sign test: *p* = 0.0005). The correlations were strongest for Glut 1 (*r* = +0.50 to +0.61 in cortex and *r* = −0.25 to −0.50 in hippocampus). Similarly, GLUT1 levels were correlated with arm entries in the Y maze (*r* = +0.50 in cortex and *r* = −0.42 in hippocampus) and all three correlations in the cortex were positive and the three correlations in the hippocampus were negative as were observed for the percent alterations in the Y maze.

In the visible platform water maze trials, higher hippocampal glucose transporter levels were linked to lower swim speeds, cumulative distance to the platform, and latency values as well as lower cumulative distance to the target location and more time spent in the target quadrant in the first probe trial (Supplementary Table 3C). The opposite pattern was seen for higher cortical GLUT1 levels.

### 3.11 Hippocampal sphingolipid analysis and its correlation with functional behavioral measures

In the standard exposure experiment, hippocampal sphingolipid analysis revealed that E4 mice had nearly double as high ceramide and sphingomyelin levels than E3 mice and that male mice had higher level of glycosylated ceramide than female mice. In the extended exposure experiment, mice had 8-times higher levels of sphingomyelins and 2.5-higher levels of glycosylated ceramides than in the short-term experiment. No consistent treatment effects were observed in either experiment (Supplementary Table 4A–C).

Stronger fear memory responses were linked to higher ceramide levels in the hippocampus (Table 3). Freezing during the third and fourth tones and freezing between the tone-shock intervals during fear conditioning training and cued fear memory were positively correlated with ceramide levels.

**TABLE 3:** Association (Spearman correlations) between fear conditioning (1–4 refers to trial number) and sphingolipid group levels in the standard exposure experiment^a^.

	Sphinganine	Ceramides	Sphingomyelin	Glycos. Ceramides	Total
Acquired Fear Response^b^:					
Baseline freeze	−0.113	−0.138	−0.073	−0.073	−0.104
Baseline motion	−0.085	+0.151	+0.023	+0.106	+0.005
Tone freeze 1	−0.047	+0.069	+0.035	+0.228	−0.042
Tone freeze 2	−0.312	+0.147	+0.165	−0.028	−0.165
Tone freeze 3	−0.058	+0.274	+0.320*	−0.035	+0.122
Tone freeze 4	−0.087	+0.112	+0.064	−0.144	−0.097
Shock motion 1	−0.037	+0.031	+0.185	−0.199	−0.090
Shock motion 2	+0.004	−0.030	+0.072	−0.310+	−0.057
Shock motion 3	+0.197	+0.050	+0.123	−0.199	+0.106
Shock motion 4	+0.132	−0.014	+0.098	−0.123	+0.049
Interval freeze 12	−0.198	+0.408**	+0.269	+0.074	−0.003
Interval freeze 23	−0.352*	+0.259	+0.134	−0.033	−0.230
Interval freeze 34	−0.375*	+0.226	+0.147	−0.125	−0.226
Contextual Fear Response					
Freeze (% time)	+0.015	+0.088	+0.017	−0.041	−0.091
Cued Fear Response					
Baseline Freeze	+0.160	+0.304*	+0.322*	+0.071	+0.170
Tone Freeze	+0.031	+0.256	+0.334*	−0.029	+0.158
Baseline Freeze (%)	+0.011	+0.446**	+0.369*	+0.318*	+0.214
Tone Freeze (%)	+0.091	+0.065	+0.237	−0.202	+0.153

a Significant effects are indicated in bold.

b Freeze is expressed in seconds per minute, freeze (%) refers to % of time frozen, motion is expressed in cm. 0.05 > *P* > 0.01, *; 0.01 > *P* > 0.001, **; *P* < 0.001, ***

### 3.12 Hippocampal metabolomics analysis

We identified consistently 115 metabolites. In E3 mice, XN altered 20 metabolites (10 higher and 10 lower in XN-treated mice); the effect of XN differed between exposure lengths (11 treatment x exposure length interactions at *p* < 0.05), sexes (23 treatment × sex interactions), and by exposure length and sex (15 treatment x exposure length × sex interactions). In the standard exposure experiment, XN altered 15 metabolites (10 higher and 5 lower in XN-treated mice) at *p* < 0.05. However, the effect of XN differed among genotypes (21 treatment × genotype interactions at *p* < 0.05), between sexes (5 treatment × sex interactions), and by genotype and sex (3 treatment x genotype × sex interactions).

**FIGURE 6:**
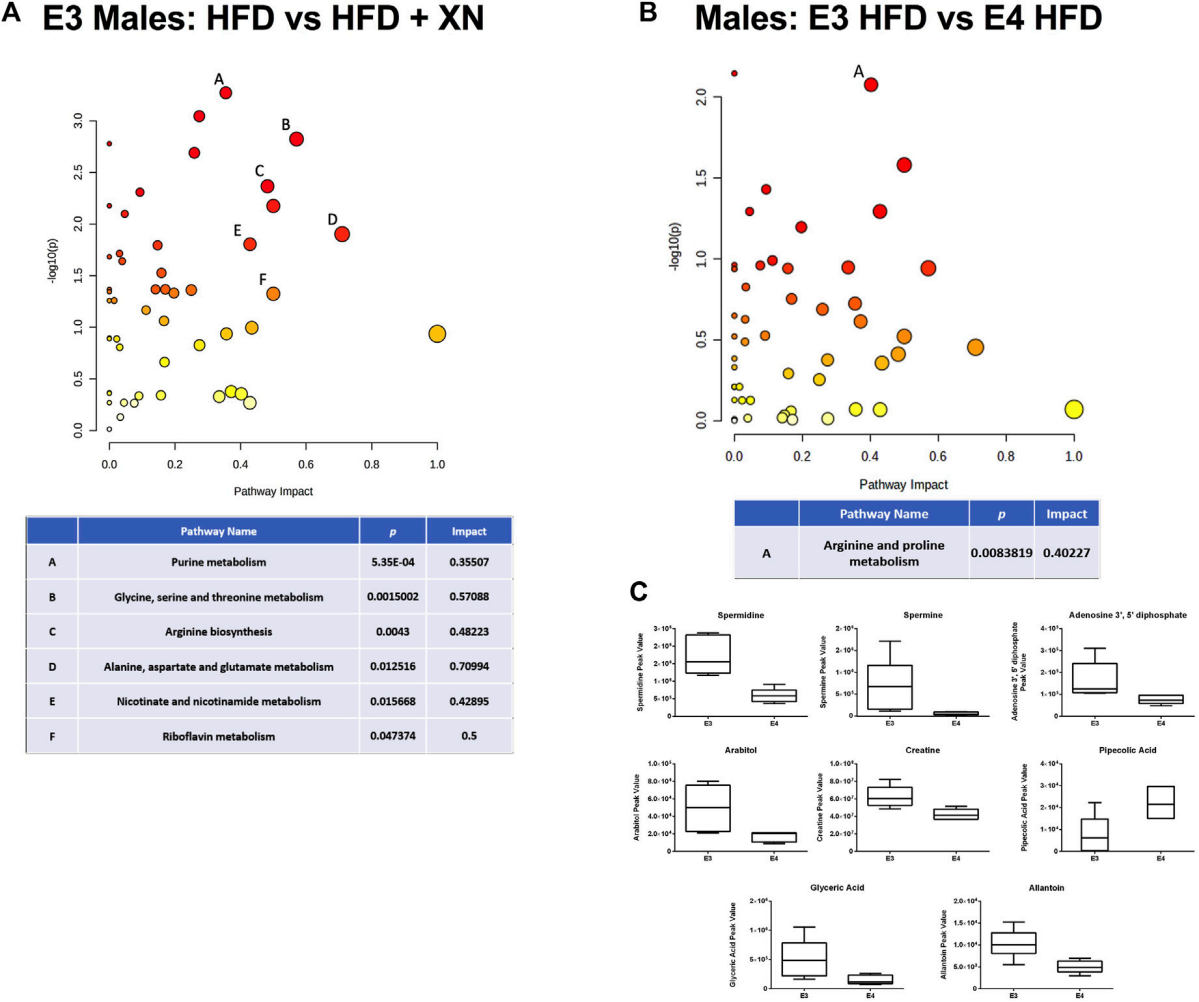
**(A)** Metabolic pathways affected by XN in the hippocampus of E3 males in the standard exposure experiment. **(B)** Differential effects of HFD in E3 and E4 males. Significantly altered pathways (*p* < 0.05) and with an Impact factor >0.3 are shown in **(A,B)**. **(C)** The levels of spermidine, spermine, adenosine 3′,5′ diphosphate, arabitol, creatine, glyceric acid, and allantoin were higher in E3 than E4 HFD males. Significant metabolites were based on a volcano plot analysis. The volcano plot is a combination of fold change analysis and t-tests.

Among genotype and sex groups, the strongest impact of XN-treatment were observed in E3 males in the standard exposure experiment (13 metabolites higher and 28 lower in XN-treated mice) (Figure 6A). Among neurometabolites, arginine, glutamine and GABA levels were higher whereas asparagine (*p* = 0.03), n-acetyl asparagine (*p* = 0.09), and n-acetyl aspartic acid (*p* = 0.07) levels were lower in XN-treated mice (Supplementary Table 6A). Among nucleic acid metabolites, there was a shift from nucleotides to nucleic acid metabolites with XN treatment (Supplementary Table 7A). Five of 10 nucleotides, n6-(delta2-isopentenyl)-adenine, and cytosine were significantly lower in XN-treated mice, and nucleic acid metabolites (inosine, hypoxanthine, xanthine, uridine and at *p* = 0.07 uracil) were higher in XN-treated mice. Except for neurometabolites and serine, XN-treatment resulted in lower levels of peptides, amino acids, and their metabolites (Supplementary Table 8A). Specifically, levels of lysine and its metabolites, methionine and its metabolites, histidine and its metabolites, antioxidants and their metabolites, were lower in XN-treated mice. Treatment with XN upregulated the citric acid cycle, as fumaric acid, malic acid, and citric acid at *p* = 0.08 were higher and methylmalonate or succinate were lower in XN-treated mice (Supplementary Table 9A). XN Treatment was linked to lower levels of urea cycle metabolites (*i.e.*, citrulline and ornithine; Supplementary Table 9A) and lower levels of medium-to long-chain fatty acid levels and higher levels of glyceric acid (Supplementary Table 10A). Among 11 the vitamins and their metabolites, XN-treated mice had, except for nicotinamide, lower levels than HFD-treated mice (Supplementary Table 11A). Differential effects of a HFD on metabolic pathways in E3 and E4 mice are shown in Figure 6B. The levels of spermidine, spermine, adenosine 3′,5’ diphosphate, arabitol, creatine, glyceric acid, and allantoin were higher in E3 than E4 HFD males (Figure 6C).

**FIGURE 7:**
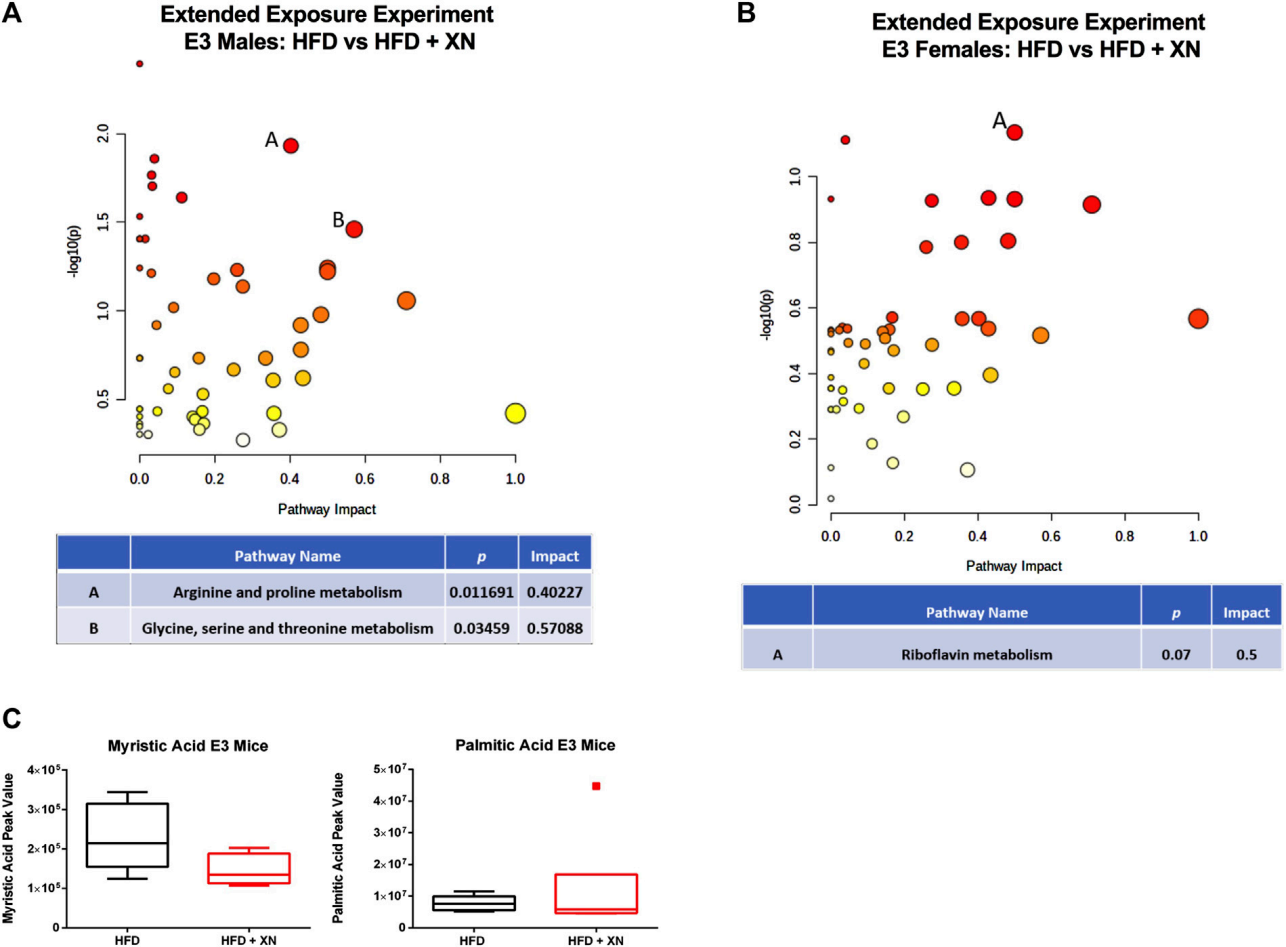
**(A)** Metabolic pathways affected by XN in the hippocampus of E3 males in the extended exposure experiment. Significantly altered pathways (*p* < 0.05) and with an Impact factor >0.3 are shown. **(B)** In E3 females, there was a trend towards the riboflavin metabolism being affected. **(C)** Myristic acid levels were reduced in XN-treated E3 males.

In the extended exposure experiment, treatment with XN increased metabolite levels in E3 males (18 metabolites higher and 4 lower in XN-treated mice) (Figure 7A). Among the 18 significantly upregulated metabolites, 6 were neurometabolites (aspartic acid, n-acetyl-aspartic acid, glutamine, n-acetyl glutamic acid, GABA, and taurine; Supplementary Table 6C), 4 were nucleotides (another 4 of 10 identified nucleotides were higher were higher at 0.10 > *p* > 0.05 in XN-treated mice; Supplementary Table 7C), and 3 were vitamins (FAD, NAD, and nicotinamide; Supplementary Table 11C). In addition, hypoxanthine (Supplementary Table 7C), alanine, creatine, and aminoadipic acid or methyl-L-glutarate (Supplementary Table 8C) were higher in XN-treated E3 mice. In contrast, XN-treatment resulted in lower fatty acid levels, as the phospholipid precursor, phosphocholine, was higher and medium-to long-chain fatty acids as well as sphingolipids and desmosterol were lower in XN-treated E3 males (Supplementary Table 10C).

In E3 females, treatment with XN lowered metabolite levels (11 metabolites higher and no lower in XN-treated mice) in the extended exposure experiment, specifically metabolites linked to nucleotide metabolism (Figures 7B,C). Seven of 10 nucleotides were lower at *p* < 0.05 and 1 more nucleotide, CMP, were lower at 0.10 > *p* > 0.05 in XN-treated mice (Supplementary Table 7C). In addition, the nucleotide precursor glutamine (Supplementary Table 6C), inosine (Supplementary Table 7C), and the vitamins pantothenic acid and FAD (Supplementary Table 11C) were lower in XN-treated E3 females. No metabolite was significantly higher in XN-treated E3 females.

XN-treatment had a low impact in E4 mice (Supplementary Tables 6–11B), as 0 and only 4 metabolites (mandelic acid and 3,4-dihydroxphenyl-acetate higher and ethanolamine phosphate and sphinganine lower in XN-treated mice) were altered at *p* < 0.05 by XN in E4 female and male E4 mice, respectively. Treatment with XN had also a low impact in E3 females in the standard exposure experiment (Supplementary Tables 6–11A), as only 2 metabolites (arginine and citric acid higher and 0 lower in XN-treated mice) were altered by XN.

**FIGURE 8:**
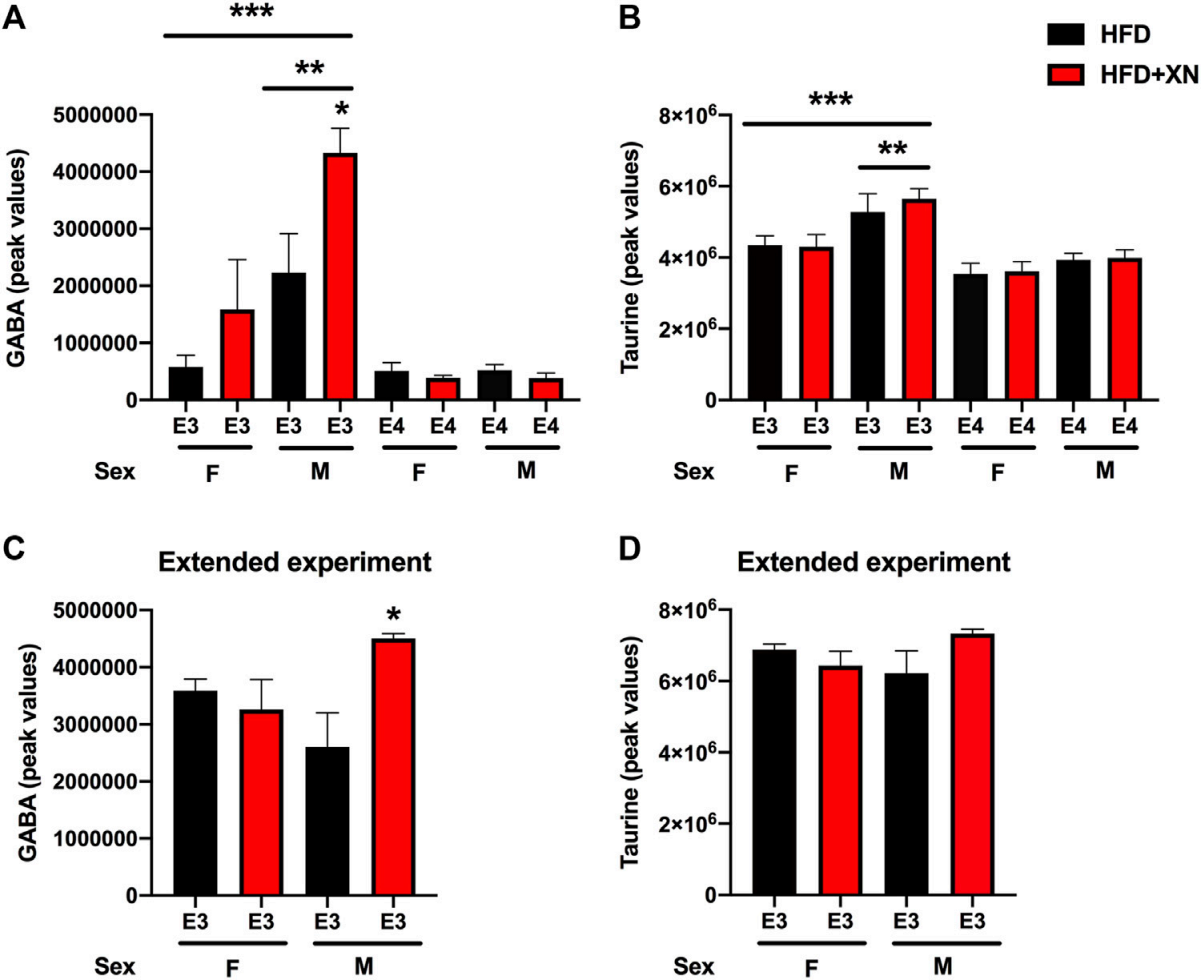
Hippocampal GABA and taurine levels. **(A)** In the standard exposure experiment, XN increased GABA levels in E3 males. *p = 0.0265. GABA levels were higher in E3 males than E3 females. **p = 0.0036. GABA levels were higher in E3 than E4 mice. ***p = 0.0002. **(B)** In the standard exposure experiment, taurine levels were higher in E3 males than females. **p = 0.0039. Taurine levels were also higher in E3 than E4 mice. ***p < 0.0001. **(C)** In the extended experiment, XN also increased GABA levels in E3 males. *p = 0.0265. **(D)** No effect of XN on taurine levels were seen in the extended experiment.

In the standard exposure experiment, XN increased GABA levels in E3 males (Figure 8A). GABA levels were higher in E3 males than E3 females and higher in E3 than E4 mice. In the standard exposure experiment, taurine levels were higher in E3 males than females and higher in E3 than E4 mice (Figure 8B). In the extended experiment, XN also increased GABA (Figure 8C), but not taurine (Figure 8C), levels in E3 males.

### 3.13 Correlations between behavioral and cognitive measures and hippocampal metabolites

Behavioral and cognitive measures were organized in 6 groups (novel object recognition and performance in the Y maze); water maze-visible platform training; water maze-hidden platform training; spatial memory retention in the water maze probe trial; performance in the fear conditioning test (various performance measures during fear learning and cued fear memory). Most correlations were observed with performance measures in the open field trials (39% of trial/metabolite combinations were significant at *p* < 0.05), followed by performance during the hidden platform water maze training (32%), and performance during visible platform water maze learning (26%). Less correlations were observed with measures in the fear conditioning test (9%), and performance in the water maze-probe trial (8%) and few significant correlations were observed with performance in the novel object and Y-maze tests (1%).

Among neurometabolites, explorative activity in the open field test was correlated with higher mandelic acid (adrenaline and noradrenaline metabolite), kynurenic acid (NDMA receptor antagonist), and asparagine levels and lower levels of glutamine, taurine, GABA, arginine, N-acetyl-L-aspartic acid, and aspartic acid (Supplementary Table 12A). Learning to locate the visible platform in the water maze was linked to higher levels of mandelic acid, kynurenic acid, asparagine, and glycine and lower levels of glutamine, GABA, and taurine (Supplementary Table 12B). Learning to locate the hidden platform water maze was linked to higher levels of mandelic acid, kynurenic acid, asparagine and glycine and lower levels of glutamine, GABA, arginine and taurine (Supplementary Table 12C). Cued fear memory was linked to lower levels of arginine and GABA (Supplementary Table 12D).

Among nucleotides and their metabolites, explorative activity in the open field test was correlated with higher levels of most nucleotide metabolites and lower levels of nucleotides, inosine and hypoxanthine (Supplementary Table 13A). Learning to locate the visible platform in the water maze was linked to higher levels of N6-(delta2-Isopentenyl)-adenine, deoxycytidine, cyclic AMP, thymine, cytosine, and 2′-deoxyguanosine 5′-MP and lower levels of most nucelotides, inosine, hypoxanthine, and methylthioadenosine (Supplementary Table 13B). Learning to locate the hidden platform in the water maze was linked to higher levels of N6-(delta2-Isopentenyl)-adenine, cyclic AMP, thymine, and orotic acid and lower levels of uridine, inosine, IMP, ADP, and methyladenosine (Supplementary Table 13C). Cued fear memory was linked to higher levels of orotic acid and UDP-Nacetylglucosamine (Supplementary Table 13D).

Among amino acids, peptides, and amino acid metabolites, explorative activity in the open field test was correlated with higher levels of most amino acids, peptides, and amino acid metabolites and lower levels of creatine metabolites, carnosine, and glutathione (Supplementary Table 14A). Learning to locate the visible platform in the water maze was linked to higher levels of most amino acids, peptides, and amino acid metabolites and lower levels of creatine metabolites, carnosine, and glutathione (Supplementary Table 14B). Learning to locate the hidden platform in the water maze was linked to higher levels of most amino acids, peptides, and amino acid metabolites and lower levels of creatine metabolites, carnosine, and glutathione (Supplementary Table 14C). Cued fear memory was linked to higher levels of 3,4-dihydroxyphenyl-acetate and pipecolic acid and lower levels of 5-oxo-proline and creatine metabolites (Supplementary Table 14D).

Explorative activity in the open field test was correlated with higher levels of phospholipids, TCA cycle intermediates, FAD, NAD, and nicotinamide and lower levels of medium- and long-chain fatty acids, urea cycle intermediates, p-aminobenzoic acid, 4-pyridoxic acid, FA, nicotinic acid, riboflavin, and lumichrome (Supplementary Table 15A). Learning to locate the visible platform water maze was linked to higher levels of medium- and long-chain fatty acids, methylmalonic acid/succinic acid, urea cycle metabolites, p-aminobenzoic acid, 4-pyridoxic acid, FA, nicotinic acid, riboflavin, and lumichrome and lower levels of phospholipids and their metabolites, TCA cycle metabolites, and nicotinic acid (Supplementary Table 15B). Learning to locate the hidden platform water maze was linked to higher levels of medium- and long-chain fatty acids, methylmalonic acid/succinic acid, p-aminobenzoic acid, 4-pyridoxic acid, nicotinic acid, riboflavin, and lumichrome and lower levels of phospholipids and their metabolites (Supplementary Table 15C). Fear memory was linked to higher levels of medium- and long-chain fatty acids (Supplementary Table 15D).

## 4 Discussion

A HFD (60% kcal from fat) was used to induce obesity in order to mimic the nutritional conditions that cause metabolic syndrome in humans. Diet-induced obesity is a well characterized model of metabolic syndrome in humans, and leads to dysfunctional lipid and glucose metabolism as well as changes in body weight gain compared to mice fed a standard laboratory diet (Gallou-Kabani et al., 2007; Hoffler et al., 2009). The dose of XN in the diet was chosen to reflect a dose which was shown to have the most pronounced benefits on parameters of metabolic syndrome in HFD-induced obese mice in our previous work (Miranda et al., 2016). The results of this study show that the ability of XN to mitigate cognitive impairments in the Y maze associated with consumption of a HFD starting at 6 months of age are apoE isoform- and sex-dependent. In the standard exposure experiment, XN treatment increased the spontaneous alternation in E3, but not in E4 or WT, mice. XN treatment also increased spontaneous alternation in E3 mice in the extended exposure experiment. The effect of XN in the Y maze was also sex-dependent; XN increased spontaneous alternation in females but decreased it in males. XN improved cognitive performance in the object recognition test and spatial memory retention in the water maze probe trial. In the water maze, beneficial effects of XN were observed in genotype and sex groups that did not do well on only the HFD. XN improved performance of XN-treated vs. HFD only-treated E3 male mice in the extended exposure experiment. In the extended exposure experiment, there was a sex × treatment interaction. XN treatment improved the performance of the E3 males but not of E3 females. XN enhanced contextual fear memory.

Specific hippocampal metabolic pathways might mediate the effects of XN in E3 males. In E3 males, different metabolic pathways in the hippocampus were affected by XN in the standard and extended exposure experiments. In the standard exposure experiment, purine metabolism, glycine, serine, and threonine metabolism, arginine biosynthesis, alanine, aspartate, and glutamate metabolism, nicotinate and nicotinamide; and riboflavin metabolism were affected by XN. In the extended exposure experiment, the arginine and proline metabolism and the glycine, serine, and threonine metabolism were affected by XN in E3 males. Myristic acid levels were decreased in XN-treated E3 males. These effects were sex-dependent and not seen in E3 females. E3 males were also particularly sensitive to effects of XN on hippocampal GABA and taurine levels. In the standard and extended exposure experiments, XN increased GABA levels in E3 males. The apoE isoform-dependent effects of XN on behavioral and cognitive performance and molecular measures are striking considering the role of apoE in Alzheimer’s pathology in mutants mice (Bales et al., 1997) and the enhanced ability of human apoE3 or apoE4 to delay AD pathology compared to mouse apoE (Fagan et al., 2002).

Glucose transporters might mediate some of the effects of XN on behavioral and cognitive performance. With the exception of E3 mice, XN-treated mice had higher glucose transporter protein levels in the hippocampus and cortex than HFD only-treated mice. In addition, stronger fear memory responses were linked to higher glucose transporter levels in the hippocampus and lower glucose transporter levels in the cortex. Freezing between the tone-shock intervals, a measure of fear learning, and cued fear memory were positively correlated with glucose transporter levels in the hippocampus and negatively correlated with glucose transporter levels in the cortex. The direction of these relationships was test and measure specific. In contrast to the fear conditioning test, activity levels in the open field, arm entries in the Y maze, swim speeds during visible platform training in the water maze, and spatial memory retention in the first water maze probe trial were linked to lower glucose transporters. The central effects of XN described above might be secondary to cardiovascular effects and/or effects of XN in the liver and/or intestine. Future efforts are warranted to explore this.

There were also molecular measures not affected by XN that were related to cognitive measures. Stronger fear memory responses were linked to higher ceramide levels in the hippocampus. Fear learning and cued fear memory were positively correlated with ceramide levels.

In the current study, female and male WT mice were put on a HFD, in the absence or presence of XN, at 6 months of age. In our previous study, male WT mice were put on a HFD, in the absence or presence of XN, at 3 months age. In the earlier study, XN improved spatial learning, i.e., improved performance to locate the hidden platform, as well as spatial memory retention in the water maze probe trial. XN-treated WT male mice also crossed the previous location of the platform in the probe trial earlier than WT male mice fed a HFD only (Miranda et al., 2018). In WT male mice, those differential effects of XN might not only be due to the age of the mice and/or responsiveness to XN but also to the water maze paradigm. In the younger WT male mice, the mice received fewer training trials, including fewer visible platform training trials prior to the hidden platform training trials. Likely as a result, the initial performance to locate the hidden platform was substantially better in the current study than in the previous study. Therefore, with the lower performance there might have been an enhanced ability to detect beneficial effects of XN on performance in the water maze.

Previously, when E3 and E4 female mice were put on a HFD starting at 9 months of age for 6 months, E4 female mice showed greater glucose intolerance and worsened HFD-induced deficits in spatial learning and memory in the water maze compared to E3 female mice on a HFD (Johnson et al., 2017). Comparing the visible and hidden platform learning curves of E3 and E4 female mice on only a HFD, the pattern indicated that E4 female mice on a HFD alone performed worse than E3 female mice on a HFD alone. However, the opposite pattern was seen when spatial memory retention was assessed in the probe trial, with better cognitive performance of E4 than E3 mice on only a HFD. In addition, in the Y maze, the pattern of spontaneous alternation showed better performance in E4 than E3 mice on only a HFD. The age of the mice and length of the HFD might both have contributed to these divergent findings.

In the present study, XN did not affect weight gain in HFD WT males. Consistent with this result, previously XN did not affect weight in younger HFD WT males, while still mitigating insulin resistance and ameliorating HFD-induced deficits in spatial learning (Miranda et al., 2018). In contrast to these results, XN-treated WT male mice and Zucker male rats gained less weight than HFD only animals (Legette et al., 2013; Miranda et al., 2016). Thus, the effects of XN on weight gain in the context of a HFD don’t seem to be required for the beneficial effects of XN on brain function.

We hypothesized that given the impaired cerebral metabolism in E4 carriers, E4 and E3 mice might show differential expression of glucose transporters in the hippocampus and cortex. Indeed, E4 mice had higher glucose transporter protein levels in the hippocampus and lower glucose transporter protein levels cortex than WT mice. Similarly, E3 mice had higher glucose transporter protein levels in the hippocampus and lower glucose transporter protein levels cortex than WT mice. Female and male E4 mice had higher glucose transporter protein levels in the hippocampus and female E4 mice had lower glucose transporter protein levels in the cortex than female E3 mice. The exception were cortical levels in male E4 mice, which were higher in E4 vs. E3 comparisons.

Male mice had higher glucose transporter protein levels in the hippocampus than female mice in 14 of 18 comparisons and lower glucose transporter protein levels in the cortex than female mice in all 12 WT and E3 comparisons. The exception were glucose transporter protein levels in the cortex of male E4 mice, which were higher in 5 of 6 E4 comparisons with female E4 mice. GLUT1 is responsible for transporting glucose across the blood brain barrier (BBB). In AD, glucose transport into the brain is reduced and associated with lower GLUT1 expression (Duran-Aniotz and Hetz, 2016; Kyrtata et al., 2021). Brain levels of glucose are correlated with peripheral levels of glucose, leaving the brain vulnerable to any metabolic glucose disruption seen in the periphery (Duelli and Kuschinsky, 2001). Chronic hyperglycemia is associated with reduced GLUT1 levels in rodents (Duelli and Kuschinsky, 2001). Given that a HFD can also induce hyperglycemia (Ikemoto et al., 1996), differential GLUT1 levels might be important in mediating the detrimental effects of a HFD on the brain. However, it should be noted that we measured total GLUT protein levels, and not glucose uptake directly. Due to the presence of inactive cytosolic glucose transporters, protein levels of GLUT may not fully reflect differences in glucose uptake. Thus, future studies are warranted to measure glucose uptake more directly to confirm these results.

The pattern of hippocampal GLUT3 levels were similar to those of hippocampal GLUT1 levels, with higher levels in E4 than E3 mice. In the cortex of females, like for GLUT1 levels, GLUT3 levels were lower in E4 than E3 mice. In contrast, in the cortex of males, GLUT3 levels were higher in E4 than E3 mice. The pattern of hippocampal GLUT4 levels were similar to those of hippocampal GLU1 and GLUT3 levels, with higher levels in E4 than E3 mice. In contrast to the hippocampus and in contrast to GLUT1 and GLUT3 levels in the cortex, in females cortical levels of GLUT4 were lower in E4 than E3 mice and increased following XN treatment. In summary, the pattern of genotype- and sex-dependent changes in GLUT1, GLUT3, and GLUT4 hippocampal and cortical levels and their response to XN seems complex and they do not seem to easily relate to cognitive performance, behavioral alterations, and hippocampal metabolic pathways in the mice.

The levels of spermidine, a polyamine compound in ribosomes and living tissues and associated with memory, in the hippocampus of HFD males were dramatically lower in E4 than in E3 animals. Reduced spermidine levels might contribute to AD risk of E4 carriers. Spermidine/Spermine is expected to be inversely associated with brain Aβ load/AD risk. Spermidine levels are lower in Dutch-type hereditary cerebral amyloid angiopathy (CAA) mutation carriers that are at increased AD risk (Chatterjee et al., 2021). Oral supplementation with spermidine has been reported to positively influence cognitive performance in older adults at risk of AD, possibly through interaction with NMDA receptors (Eisenberg et al., 2009; Madeo et al., 2018). This mechanism may induce autophagy in glial or neuronal cells, and thus suppresses inflammation.

Our results indicate that more hippocampal metabolic pathways might mediate the effects of XN in E3 males in the standard than extended treatment experiments. In the standard exposure experiment, six metabolic pathways were affected by XN (purine metabolism, glycine, serine, and threonine metabolism, arginine biosynthesis, alanine, aspartate, and glutamate metabolism, nicotinate and nicotinamide; and riboflavin metabolism), while in the extended exposure experiment, two metabolic pathways were affected by XN (the arginine and proline metabolism and the glycine, serine, and threonine metabolism). Myristic acid levels were decreased in XN-treated E3 males. E3 males were also particularly sensitive to effects of XN on hippocampal GABA and taurine levels. In the standard and extended exposure experiments, XN increased GABA levels in E3 males. These data suggest that increased hippocampal GABA levels might mediate some of the beneficial effects of XN in the context of HFD-induced cognitive impairments.

Among hippocampal neurometabolites, learning to locate the visible platform in the water maze, learning to locate the hidden platform water maze, and cued fear memory were linked to lower levels of GABA. These data are consistent with the disruption of fear memories following pre- and post-training administration of drugs that facilitate GABA neurotransmission (Makkar et al., 2010) and the increase ratio in glutamate to GABA in hippocampus and visual neocortex when human study participants recalled visual cues in a virtual environment in which specific sounds predicted the appearance of particular visual patterns and some patterns led to a monetary award (Koolschijn et al., 2021). As cued fear memory is not hippocampus-dependent (Phillips and Ledoux, 1992), these data suggest that these relationships are not necessarily restricted to the hippocampus. It is remarkable that while this relationship was seen with hippocampus-dependent spatial learning in the water maze, it was not seen with performance on other hippocampus-dependent tests, including contextual fear memory and spontaneous alternation in the Y maze. These data suggest that the differential involvement of multiple brain regions in specific cognitive tests and anatomical specificity to levels of neurometabolites might contribute to these test-dependent correlative relationships.

Among nucleotides and their metabolites, learning to locate the hidden platform in the water maze and cued fear memory was linked to higher levels of orotic acid. This result is consistent with the beneficial effects of oratic acid on spatial memory in rats following fimbria fornix lesions (Gonzalez et al., 2011).

Among amino acids, peptides, and amino acid metabolites, learning to locate the hidden platform in the water maze and cued fear memory were linked to lower levels of creatine metabolites. The direction of this relationship is surprising based on the enhancement of spatial learning and memory in the water maze following dietary administration of creatine (3% in the diet) for 8 weeks prior to cognitive testing (Snow et al., 2018) and the potential for creatine supplementation to improve cognitive performance (Roschel et al., 2021). However, while typically creatine and creatine metabolites are positively related to each other, the relationship might be more complex (Weiss and Kaddurah-Daouk, 2000).

Learning to locate the hidden platform water maze and cued fear memory was linked to higher levels of medium- and long-chain fatty acids. Consistent with this result, in brain, the highest levels of polyunsaturated free fatty acids are seen in brain areas involved in fear learning and memory like the amygdala and auditory fear conditioning increases free fatty acids, particularly saturated ones, in the amygdala and prefrontal cortex (Wallis et al., 2021).

In a previous study conducted by us, we examined the hepatic expression of a panel of nuclear receptors including the constitutive androstane receptor (CAR), pregnane X receptor (PXR), and glucocorticoid receptor (GR) in liver-specific farnesoid X receptor (FXR)-null mice and WT mice that were fed a high-fat diet and treated orally with XN XN treatment induced the expression of CAR and PXR in all sex and genotype groups, whereas an increase in GR transcript levels was observed in hepatic FXR-null mice only. These findings led us to conclude that XN ameliorates diet-induced liver dysfunction *via* FXR-dependent and independent pathways and that deficiency in one nuclear receptor (FXR) is compensated for by increased expression of other nuclear receptors (CAR, PXR, GR) with partially overlapping functions such as phase I and II metabolism. FXR agonism by XN would stimulate the production of FGF15/19, which displays insulin-like actions. Increased GR expression would stimulate GRα signaling and gluconeogenesis. As GRβ signaling closely recapitulates insulin (Sepúlveda-Quiñenao et al., 2022), our findings with XN may in part explain the effect of XN on glucose uptake in the hippocampus *via* GR induction.

In summary, the results of this study show that the ability of XN to mitigate cognitive impairments associated with consumption of a HFD starting at 6 months of age are apoE isoform- and sex-dependent. As the rates of diet-induced obesity continue to rise, with recent estimates indicating that 71.6% of American adults are overweight or obese (Paraiso et al., 2021), increased efforts are warranted to assess the effects of XN on HFD-induced cognitive impairments in middle-aged and aged mice and whether these effects are apoE isoform- and sex-dependent and associated with alterations in specific hippocampal pathways and/or primarily driven by peripheral effects of XN using similar or different pathways as those affected by XN in brain.

## Data Availability

The original contributions presented in the study are included in the article/Supplementary Material, further inquiries can be directed to the corresponding author.
