# Supplementary figures and images for: ApoE isoform-dependent effects of xanthohumol on high fat diet-induced cognitive impairments and hippocampal metabolic pathways
Source: Front Pharmacol. 2022 Oct 3;13:954980. doi: 10.3389/fphar.2022.954980 (PMC9583926; doi:10.3389/fphar.2022.954980)

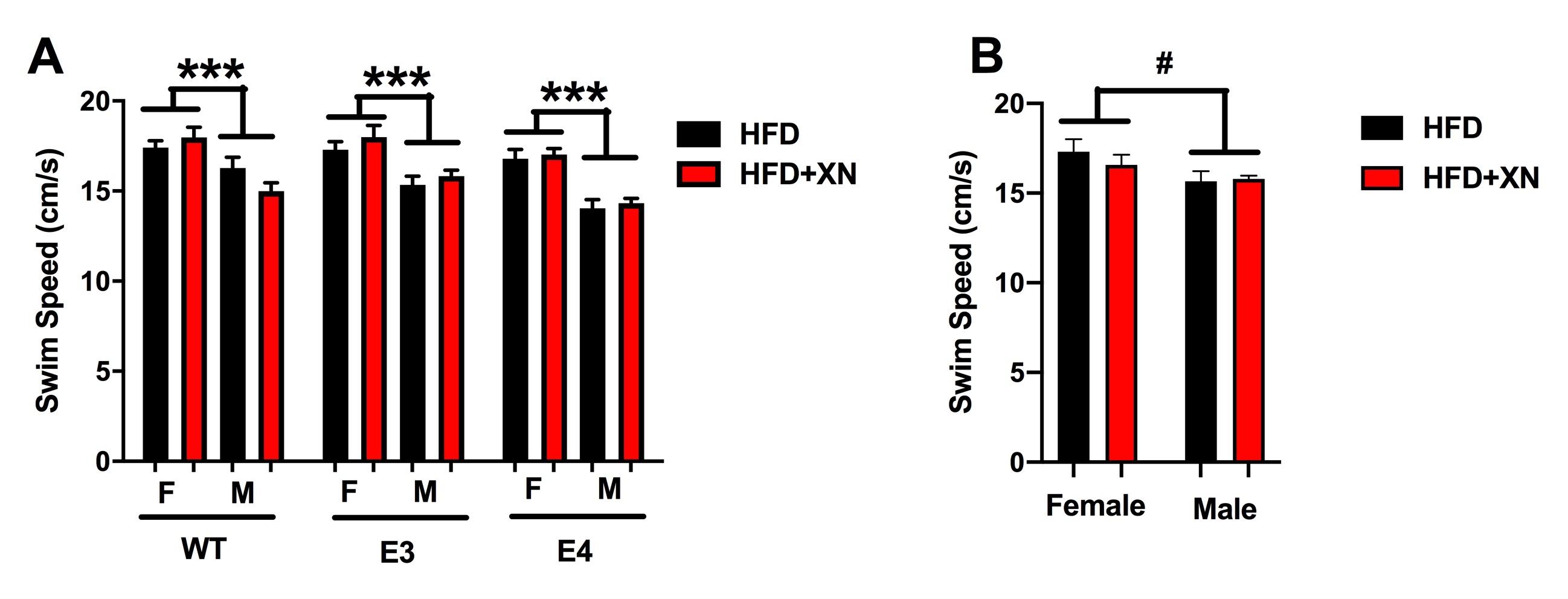

Supplement: Supplementary file 1 [file Image3.JPEG]

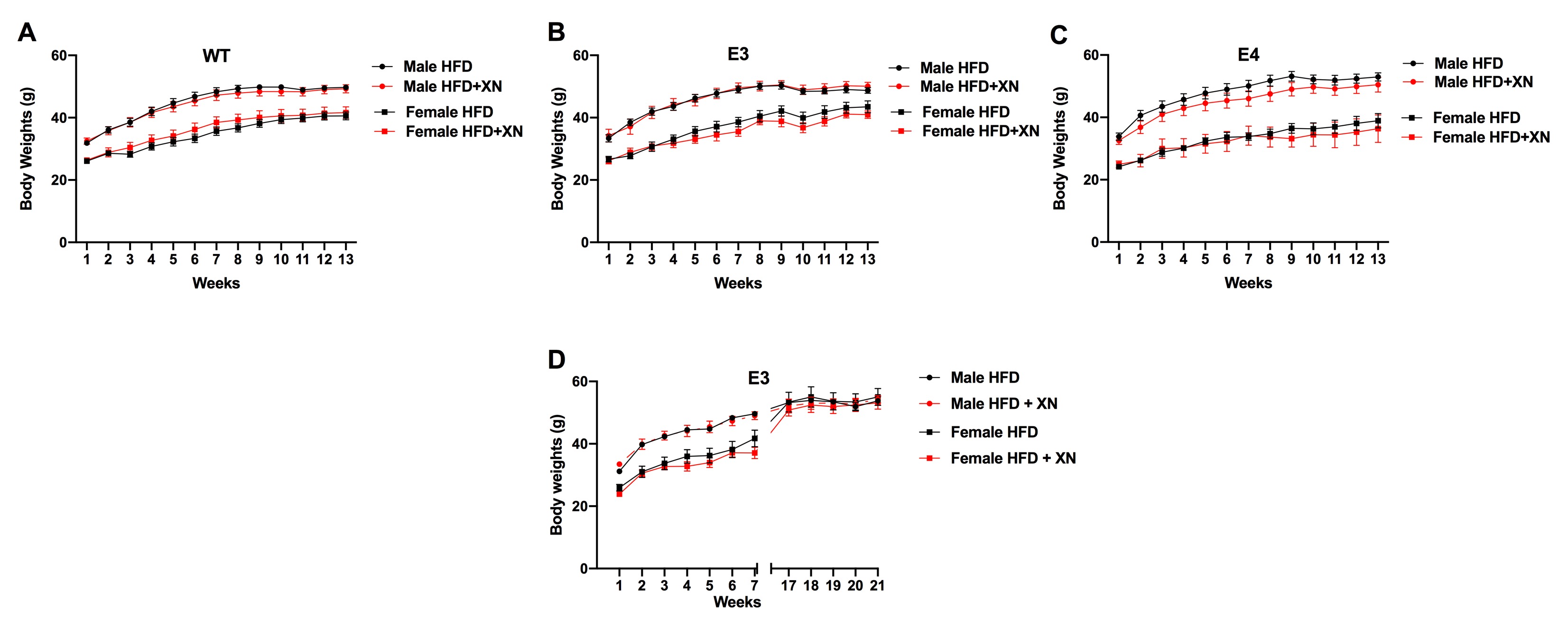

Supplement: Supplementary file 2 [file Image1.JPEG]

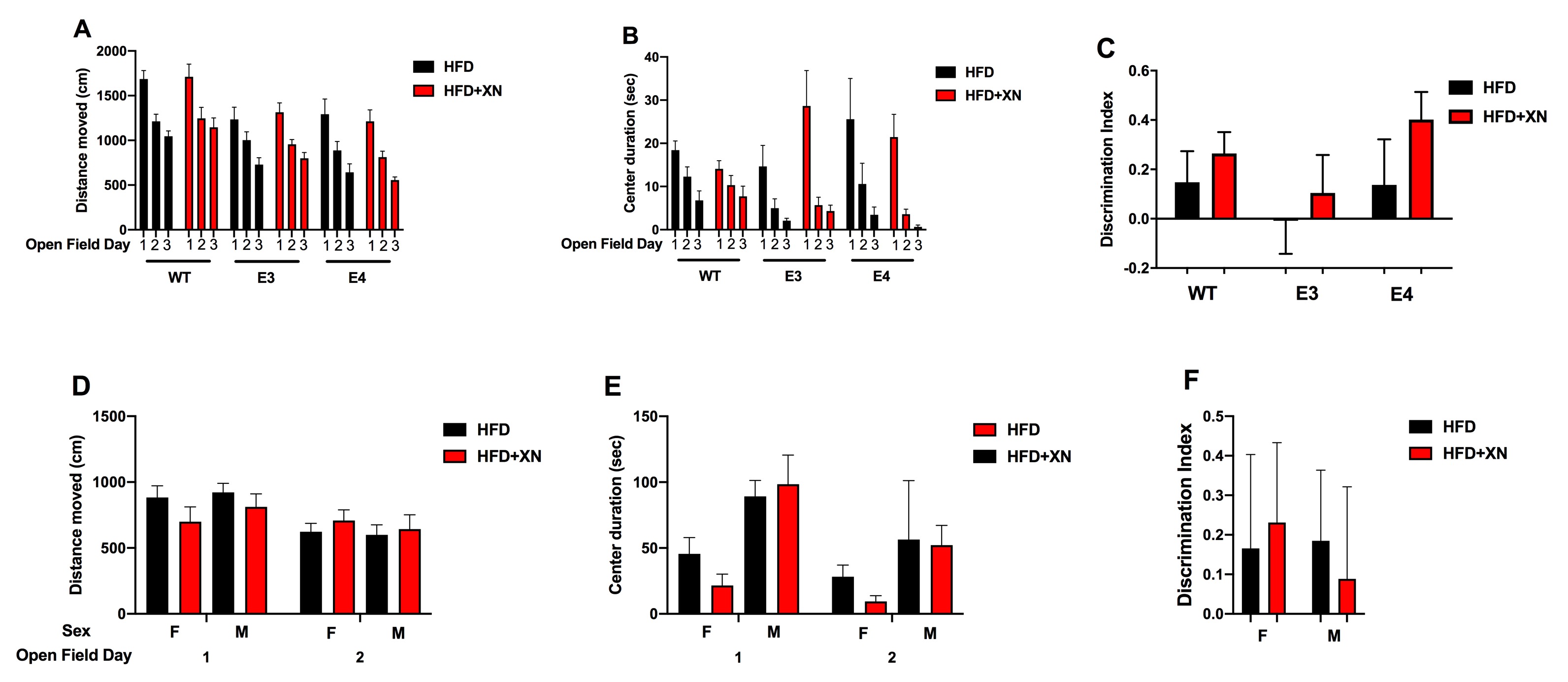

Supplement: Supplementary file 4 [file Image2.JPEG]

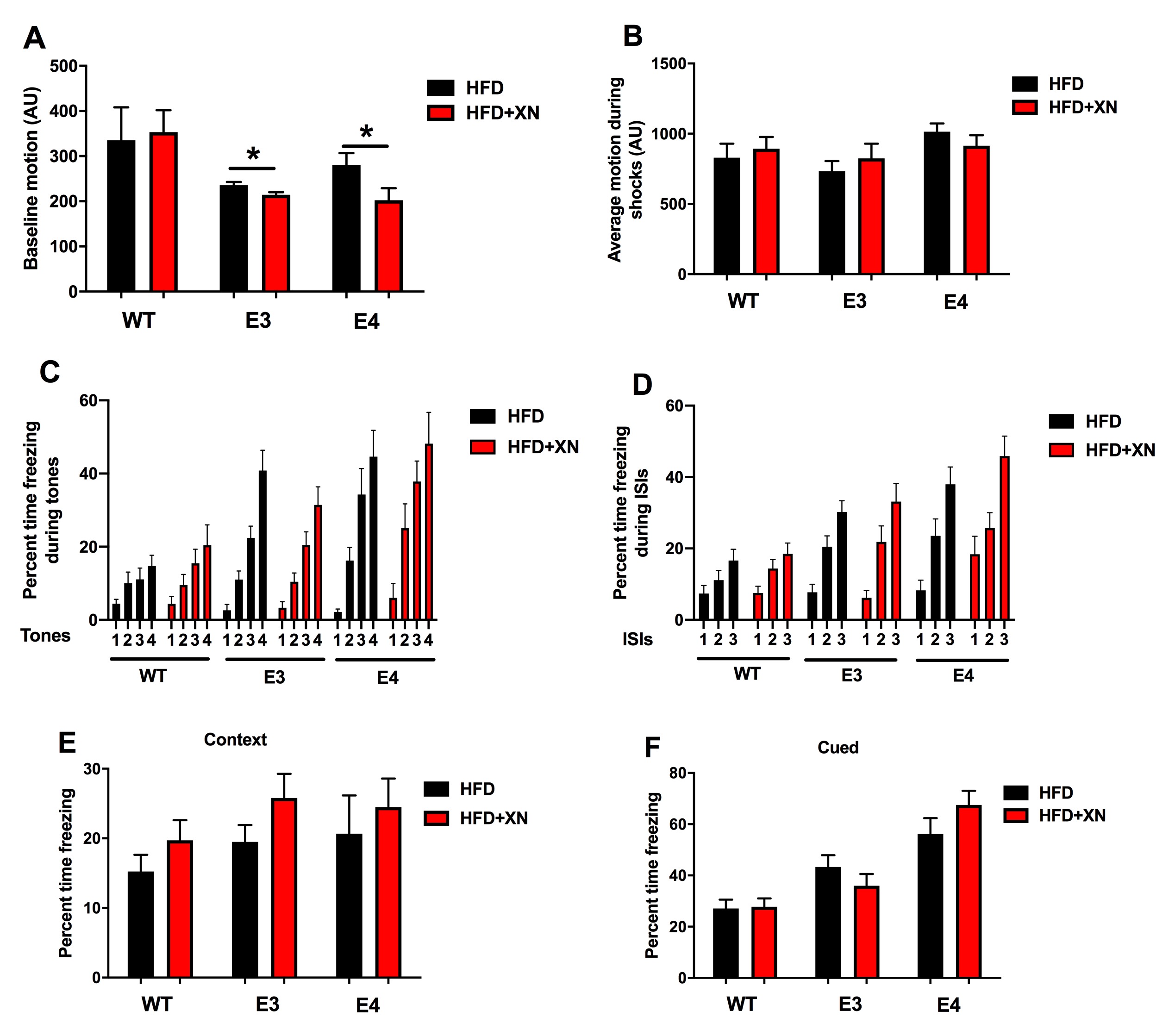

Supplement: Supplementary file 5 [file Image5.JPEG]
