# Supplementary material for: ApoE isoform-dependent effects of xanthohumol on high fat diet-induced cognitive impairments and hippocampal metabolic pathways
Source: Front Pharmacol. 2022 Oct 3;13:954980. doi: 10.3389/fphar.2022.954980 (PMC9583926; doi:10.3389/fphar.2022.954980)
Supplement: Supplementary file 7 [file DataSheet2.docx]

**Supplementary Table 1A.** Association (Spearman correlations) between fear conditioning (1 – 4 refers to trial number) and glucose transporter protein levels (in ng/mg protein) in the hippocampus and cortex in the standard exposure experiment ^1^.

|  | Hippocampus | | | |  |  | Cortex |  |  |
| --- | --- | --- | --- | --- | --- | --- | --- | --- | --- |
|  | Glut 1 | | | | Glut 3 | Glut 4 | Glut 1 | Glut 3 | Glut 4 |
| Acquired Fear Response^2^: | | |  | |  |  |  |  |  |
| Baseline freeze | -0.044 | | | | -0.128 | -0.035 | +0.003 | -0.062 | +0.040 |
| Baseline motion | -0.121 | | | | -0.078 | -0.134 | +0.176 | -0.029 | +0.085 |
| Tone freeze 1 | -0.036 | | | | -0.138 | -0.076 | -0.091 | -0.058 | +0.030 |
| Tone freeze 2 | +0.068 | | | | -0.069 | +0.108 | -0.122 | +0.023 | +0.026 |
| Tone freeze 3 | +0.266** | | | | +0.152 | +0.236* | -0.232* | +0.104 | +0.043 |
| Tone freeze 4 | +0.165 | | | | +0.079 | +0.225* | -0.251* | +0.092 | -0.063 |
| Shock motion 1 | +0.085 | | | | +0.017 | -0.068 | +0.065 | +0.022 | +0.138 |
| Shock motion 2 | +0.136 | | | | -0.015 | -0.058 | +0.091 | +0.188 | +0.079 |
| Shock motion 3 | +0.192 | | | | +0.004 | -0.044 | -0.027 | +0.117 | +0.088 |
| Shock motion 4 | +0.172 | | | | -0.024 | -0.028 | +0.035 | +0.089 | +0.118 |
| Interval freeze 12 | +0.142 | | | | -0.022 | +0.088 | -0.091 | +0.127 | +0.246* |
| Interval freeze 23 | +0.180 | | | | +0.027 | +0.140 | -0.189 | +0.042 | +0.026 |
| Interval freeze 34 | +0.376*** | | | | +0.209* | +0.332*** | -0.184 | +0.198* | -0.053 |
| Contextual Fear Response | | | |  |  |  |  |  |  |
| Freeze (% time) | +0.103 | | | | +0.072 | +0.026 | +0.003 | +0.203* | +0.161 |
| Cued Fear Response | |  | | |  |  |  |  |  |
| Baseline Freeze | +0.403*** | | | | +0.336*** | +0.334*** | -0.197* | +0.173 | +0.215* |
| Tone Freeze | +0.386*** | | | | +0.231* | +0.420*** | -0.366*** | +0.079 | -0.077 |
| Baseline Freeze (%) | +0.366*** | | | | +0.271** | +0.283** | -0.241* | +0.077 | +0.233* |
| Tone Freeze (%) | +0.341*** | | | | +0.213* | +0.281** | -0.212* | +0.234* | -0.021 |

^1^Significant effects are indicated in bold.

^2^Freeze is expressed in seconds per minute, freeze (%) refers to % of time frozen, motion is expressed in cm. 0.05 > *P* > 0.01: *; 0.01 > *P* > 0.001: **; *P* < 0.001: ***

**Supplementary Table 1B**. Association (Spearman correlations) between activity level in y-maze and open field test (1 to 3 refers to day of test) and glucose transporter protein levels (in ng/mg protein) in the hippocampus and cortex in the standard exposure experiment^1^.

|  | Hippocampus |  |  | Cortex |  |  |
| --- | --- | --- | --- | --- | --- | --- |
| Y-Maze | Glut 1 | Glut 3 | Glut 4 | Glut 1 | Glut 3 | Glut 4 |
| Arm entries, n | -0.417*** | -0.267** | -0.341*** | +0.496** | +0.049 | +0.115 |
| Open Field^2^ |  |  |  |  |  |  |
| Center duration 1 | +0.062 | -0.025 | +0.015 | +0.152 | +0.032 | +0.048 |
| Center duration 2 | -0.290** | -0.259* | -0.245* | +0.438*** | +0.091 | +0.209* |
| Center duration 3 | -0.278** | -0.166 | -0.258* | +0.450*** | +0.211* | +0.142 |
| Distance moved 1 | -0.249* | -0.248* | -0.234* | +0.504*** | +0.185 | +0.213* |
| Distance moved 2 | -0.395*** | -0.286** | -0.272** | +0.508*** | +0.132 | +0.133 |
| Distance moved 3 | -0.500*** | -0.332*** | -0.402*** | +0.605*** | +0.145 | +0.213* |

^1^Significant effects are indicated in bold.

^2^Center duration is expressed in seconds, and distance moved is expressed in cm.

0.05 > *P* > 0.01: *; 0.01 > *P* > 0.001: **; *P* < 0.001: ***

**Supplementary Table 1C.** Association (Spearman correlations) between visible platform water maze activity level (1 – 4 refers to trial number) and glucose transporter protein levels (in ng/mg protein) in the hippocampus and cortex in the standard exposure experiment^1,2^.

|  | Hippocampus |  |  | Cortex |  |  |
| --- | --- | --- | --- | --- | --- | --- |
|  | Glut 1 | Glut 3 | Glut 4 | Glut 1 | Glut 3 | Glut 4 |
| Latency 1 (sec) | -0.238* | -0.222* | -0.264** | +0.145 | +0.018 | +0.314** |
| Latency 2 (sec) | -0.312** | -0.245* | -0.396*** | +0.253* | -0.108 | +0.216* |
| Latency 3 (sec) | -0.298** | -0.201* | -0.183 | +0.221* | +0.073 | +0.306** |
| Latency 4 (sec) | -0.173 | -0.114 | -0.157 | +0.184 | -0.012 | +0.175 |
| Distance moved 1 | -0.101 | -0.142 | -0.083 | -0.057 | -0.042 | +0.141 |
| Distance moved 2 | -0.237* | -0.206* | -0.320** | +0.215+ | -0.070 | +0.180 |
| Distance moved 3 | -0.150 | -0.084 | -0.033 | +0.118 | +0.163 | +0.329*** |
| Distance moved 4 | -0.258* | -0.198* | -0.267** | +0.255* | -0.015 | +0.212* |
| Velocity 1 | -0.227* | -0.108 | -0.176 | +0.279** | +0.092 | +0.069 |
| Velocity 2 | -0.126 | -0.103 | -0.151 | +0.204* | +0.012 | -0.082 |
| Velocity 3 | +0.074 | +0.090 | +0.145 | +0.086 | +0.207* | -0.055 |
| Velocity 4 | -0.302** | -0.273** | -0.340*** | +0.419*** | +0.147 | +0.186 |

^1^Significant effects are indicated in bold.

^2^Latency is expressed in seconds, distance moved is expressed in cm, and velocity is in cm/sec.

0.05 > *P* > 0.01: *; 0.01 > *P* > 0.001: **; *P* < 0.001: ***

**Supplementary Table 2A.** Sphingolipid signal intensities affected by XN in the hippocampus of E3 mice in the standard exposure experiment^1^.

| Standard Exposure | Female |  |  |  |  | Male |  |  |  |  |
| --- | --- | --- | --- | --- | --- | --- | --- | --- | --- | --- |
| Genotype E3 | HFD |  | XN |  |  | HFD |  | XN |  |  |
| Sphingolipid | Median | P 25, 75 | Median | P 25, P75 | P-value | Median | P 25, 75 | Median | P 25, P75 | P-value |
| Sphinganine C16:0 | 3954 | 3661, 4119 | 4057 | 3790, 4985 | 0.21 | 4061 | 3833 | 3989 | 3782, 6260 | 0.40 |
| Sphinganine C18:0 | 453 | 397, 494 | 422 | 282, 481 | 0.86 | 340 | 306 | 445 | 375, 591 | 0.25 |
| Cer C34:1 | 3 | 2, 9 | 4 | 2, 6 | 0.97 | 3 | 2 | 4 | 2, 19 | 0.93 |
| Cer C36:1 | 61 | 24, 132 | 51 | 36, 66 | 0.85 | 50 | 42 | 80 | 65, 226 | 0.84 |
| Cer C38:1 | 0 | 0, 3 | 1 | 0, 2 | 0.96 | 0 | 0 | 0 | 0, 1 | 0.67 |
| Cer C34:2 | 4 | 2, 27 | 7 | 6, 10 | 0.90 | 7 | 2 | 4 | 2, 7 | 0.53 |
| Cer C36:2 | 161 | 40, 310 | 243 | 180, 389 | 0.62 | 240 | 59 | 166 | 78, 299 | 0.64 |
| Cer C38:2 | 1 | 0, 4 | 3 | 0, 6 | 0.84 | 0 | 0 | 0 | 0, 2 | 0.73 |
| Cer C42:2 | 3 | 0, 8 | 2 | 0, 7 | 0.82 | 3 | 0 | 4 | 1, 11 | 0.45 |
| SM C34:1 | 16 | 8, 100 | 65 | 15, 183 | 0.50 | 40 | 25 | 34 | 28, 39 | 0.95 |
| SM C36:1 | 60 | 33, 179 | 59 | 48, 62 | 0.81 | 68 | 36 | 97 | 70, 267 | 0.71 |
| SM C42:1 | 4 | 3, 4 | 4 | 3, 5 | 0.89 | 4 | 3 | 2 | 1, 4 | 0.26 |
| SM C34:2 | 6 | 4, 20 | 16 | 5, 23 | 0.71 | 14 | 10 | 13 | 4, 21 | 0.60 |
| SM C36:2 | 48 | 14, 216 | 83 | 47, 117 | 0.99 | 93 | 24 | 45 | 38, 123 | 0.80 |
| SM C38:2 | 2 | 2, 2 | 4 | 2, 5 | 0.73 | 3 | 2 | 4 | 4, 5 | 0.99 |
| SM C42:2 | 7 | 6, 9 | 6 | 5, 9 | 0.79 | 6 | 4 | 7 | 6, 9 | 0.62 |
| Hex2Cer C34:1 | 3 | 1, 6 | 9 | 5, 16 | 0.59 | 21 | 14 | 24 | 12, 33 | 0.53 |
| Hex1Cer C42:2 | 4 | 0, 7 | 2 | 2, 3 | 0.81 | 3 | 0 | 1 | 0, 6 | 0.63 |
| Sphinganine | 4499 | 4093, 4517 | 4585 | 4203, 5360 | 0.18 | 4484 | 4260 | 4619 | 4298, 6713 | 0.27 |
| Ceramides | 253 | 110, 389 | 297 | 216, 653 | 0.78 | 290 | 115 | 324 | 149, 646 | 0.78 |
| Sphingomyelin | 193 | 86, 531 | 317 | 114, 411 | 0.98 | 240 | 109 | 246 | 162, 369 | 0.92 |
| Glyc. Ceramides | 6 | 5, 12 | 11 | 7, 21 | 0.70 | 27 | 19 | 32 | 22, 39 | 0.36 |
| Total | 5016 | 4628, 5232 | 5672 | 4717, 5814 | 0.18 | 5259 | 4669 | 5778 | 5101, 7065 | 0.35 |

^1^Signal intensities were divided by 1000. C: carbon, Cer: ceramide, SM: sphingomyelin, Hex2Cer: dihexose-glucose, Hex1Cer: monohexose glucose, Glyc.: glycosylated ceramides.

**Supplementary Table 2B.** Sphingolipid signal intensities affected by XN in the hippocampus of E4 mice in the standard exposure experiment^1^.

| Standard Exposure | Female |  |  |  |  | Male |  |  |  |  |
| --- | --- | --- | --- | --- | --- | --- | --- | --- | --- | --- |
| Genotype E4 | HFD |  | XN |  |  | HFD |  | XN |  |  |
| Sphingolipid | Median | P 25, 75 | Median | P 25, P75 | P-value | Median | P 25, 75 | Median | P 25, P75 | P-value |
| Sphinganine C16:0 | 3734 | 3570, 4129 | 4324 | 3789, 4459 | 0.47 | 3908 | 3829, 4489 | 4070 | 3792, 4206 | 0.60 |
| Sphinganine C18:0 | 610 | 327, 613 | 400 | 358, 569 | 0.43 | **618** | **436, 746** | **387** | **312, 424** | **0.04** |
| Cer C34:1 | 7 | 7, 19 | 9 | 7, 21 | 0.90 | 12 | 8, 13 | 7 | 4, 9 | 0.74 |
| Cer C36:1 | 159 | 112, 270 | 148 | 88, 216 | 0.85 | 120 | 114, 172 | 94 | 62, 167 | 0.72 |
| Cer C38:1 | 3 | 3, 3 | 4 | 3, 6 | 0.35 | 4 | 3, 5 | 3 | 2, 4 | 0.71 |
| Cer C34:2 | **6** | **4, 9** | **21** | **12, 33** | **0.03** | 17 | 9, 29 | 18 | 15, 20 | 0.92 |
| Cer C36:2 | 257 | 240, 309 | 513 | 354, 892 | 0.06 | 402 | 233, 561 | 405 | 264, 494 | 0.47 |
| Cer C38:2 | **3** | **0, 7** | **6** | **4, 15** | **0.05** | 8 | 4, 8 | 7 | 4, 9 | 0.80 |
| Cer C42:2 | 10 | 8, 23 | 17 | 17, 20 | 0.91 | 17 | 7, 19 | 8 | 6, 11 | 0.95 |
| SM C34:1 | 58 | 46, 131 | 103 | 80, 124 | 0.57 | 95 | 58, 130 | 151 | 67, 272 | 0.07 |
| SM C36:1 | 206 | 165, 256 | 253 | 122, 397 | 0.82 | 168 | 134, 194 | 194 | 108, 254 | 0.18 |
| SM C42:1 | 6 | 3, 5 | 4 | 3, 5 | 0.22 | 3 | 3, 4 | 4 | 3, 4 | 0.18 |
| SM C34:2 | 6 | 3, 24 | 43 | 19, 375 | 0.17 | 35 | 22, 35 | 22 | 16, 32 | 0.75 |
| SM C36:2 | 99 | 78, 112 | 289 | 179, 1 | 0.27 | 182 | 124, 207 | 197 | 139, 236 | 0.28 |
| SM C38:2 | 3 | 2, 4 | 17 | 4, 31 | 0.09 | 12 | 4, 17 | 6 | 3, 7 | 0.22 |
| SM C42:2 | 14 | 12, 15 | 10 | 8, 11 | 0.12 | **6** | **6, 7** | **11** | **7, 14** | **0.02** |
| Hex2Cer C34:1 | 4 | 2, 6 | 13 | 3, 17 | 0.24 | 20 | 9, 22 | 8 | 6, 13 | 0.26 |
| Hex1Cer C42:2 | 10 | 6, 13 | 11 | 6, 14 | 0.89 | 9 | 6, 12 | 7 | 3, 9 | 0.80 |
| Sphinganine | 4264 | 4183, 4783 | 4700 | 4395, 4810 | 0.55 | 4704 | 4447, 4820 | 4413 | 4198, 4651 | 0.35 |
| Ceramides | 398 | 380, 685 | 701 | 444, 1351 | 0.15 | 633 | 395, 738 | 537 | 337, 723 | 0.68 |
| Sphingomyelin | 402 | 379, 453 | 735 | 493, 1123 | 0.42 | 528 | 352, 577 | 604 | 532, 739 | 0.40 |
| Glyc. Ceramides | 14 | 12, 25 | 27 | 13, 31 | 0.28 | 27 | 22, 27 | 17 | 9, 26 | 0.35 |
| Total | 5532 | 5353, 5594 | 6308 | 5975, 6820 | 0.15 | 6161 | 5439, 7172 | 5573 | 5554, 5664 | 0.48 |

^1^Significant effects are indicated in bold.

**Supplementary Table 2C.** Sphingolipid signal intensities affected by XN in the hippocampus of E3 mice in the extended exposure experiment^1^.

| Extended Exposure | Female |  |  |  |  | Male |  |  |  |  |
| --- | --- | --- | --- | --- | --- | --- | --- | --- | --- | --- |
| Genotype E3 | HFD |  | XN |  |  | HFD |  | XN |  |  |
| Sphingolipid | Median | P 25, 75 | Median | P 25, P75 | P-value | Median | P 25, 75 | Median | P 25, P75 | P-value |
| Sphinganine C16:0 | 5262 | 3910, 6561 | 4074 | 3931, 6828 | 0.44 | 3939 | 3926, 4024 | 4403 | 4150, 5054 | 0.56 |
| Sphinganine C18:0 | 503 | 435, 616 | 456 | 364, 539 | 0.70 | **612** | **561, 646** | **373** | **362, 456** | **0.03** |
| Cer C34:1 | 3 | 1, 6 | 5 | 3, 6 | 0.86 | 9 | 5, 11 | 3 | 1, 9 | 0.70 |
| Cer C36:1 | 121 | 73, 184 | 158 | 67, 192 | 0.94 | 130 | 88, 263 | 99 | 97, 298 | 0.92 |
| Cer C38:1 | 2 | 0, 8 | 4 | 2, 6 | 0.97 | 3 | 2, 7 | 5 | 3, 12 | 0.90 |
| Cer C34:2 | 0 | 0, 2 | 3 | 0, 9 | 0.41 | 3 | 3, 4 | 0 | 0, 0 | 0.54 |
| Cer C36:2 | 91 | 56, 180 | 163 | 29, 200 | 0.45 | 254 | 252, 370 | 71 | 33, 102 | 0.09 |
| Cer C38:2 | 0 | 0, 2 | 2 | 0, 4 | 0.43 | **4** | **3, 7** | **0** | **0, 0** | **0.02** |
| Cer C42:2 | 2 | 0, 6 | 4 | 2, 5 | 0.93 | 2 | 1, 2 | 3 | 2, 5 | 0.71 |
| SM C34:1 | 124 | 54, 262 | 163 | 26, 261 | 0.84 | **316** | **195, 341** | **72** | **59, 72** | **0.005** |
| SM C36:1 | 1502 | 1190, 1700 | 1661 | 145, 1956 | 0.66 | **1279** | **1032, 3806** | **1184** | **1086, 1489** | **0.05** |
| SM C42:1 | 6 | 2, 9 | 5 | 4, 8 | 0.71 | 4 | 2, 6 | 7 | 7, 8 | 0.09 |
| SM C34:2 | 39 | 7, 107 | 36 | 23, 77 | 0.88 | **99** | **62, 155** | **20** | **14, 31** | **0.03** |
| SM C36:2 | 491 | 96, 1114 | 433 | 113, 1006 | 0.92 | **1015** | **770, 1192** | **177** | **168, 221** | **0.02** |
| SM C38:2 | 7 | 4, 11 | 8 | 3, 20 | 0.53 | 8 | 7, 9 | 7 | 5, 12 | 0.61 |
| SM C42:2 | 19 | 14, 30 | 15 | 10, 25 | 0.36 | 11 | 10, 28 | 24 | 18, 29 | 0.39 |
| Hex2Cer C34:1 | 28 | 7, 63 | 22 | 9, 35 | 0.93 | 39 | 29, 54 | 24 | 14, 24 | 0.13 |
| Hex1Cer C42:2 | 11 | 7, 27 | 13 | 6, 29 | 0.68 | 5 | 5, 18 | 12 | 9, 26 | 0.92 |
| Sphinganine | 5697 | 4403, 7121 | 4530 | 4247, 7346 | 0.41 | 4537 | 4365, 4670 | 4722 | 4606, 5428 | 0.77 |
| Ceramides | 261 | 168, 416 | 330 | 176, 414 | 0.64 | 540 | 355, 542 | 248 | 140, 397 | 0.24 |
| Sphingomyelin | 2195 | 1435, 3293 | 2339 | 391, 3417 | 0.76 | **3320** | **2418, 5492** | **1762** | **1489, 2110** | **0.01** |
| Glyc. Ceramides | 59 | 35, 67 | 46 | 22, 57 | 0.71 | 59 | 56, 93 | 40 | 36, 53 | 0.12 |
| Total | 7610 | 6628, 10474 | 7800 | 5365, 10061 | 0.44 | 9439 | 8133,10761 | 7182 | 6817, 7528 | 0.08 |

^1^Significant effects are indicated in bold.

**Supplementary Table 3A.** Neurometabolite signal intensity levels affected by XN in the hippocampus of E3 mice in the standard exposure experiment^1^.

| Standard Exposure | Female |  |  |  |  | Male |  |  |  |  |
| --- | --- | --- | --- | --- | --- | --- | --- | --- | --- | --- |
| Genotype E3 | HFD |  | XN |  |  | HFD |  | XN |  |  |
| Metabolite | Median | P 25, 75 | Median | P 25, P75 | P-value | Median | P 25, 75 | Median | P 25, P75 | P-value |
| L-Arginine | **623** | **317, 1243** | **1416** | **529, 5785** | **0.04** | **714** | **339, 2684** | **7698** | **7312, 7912** | **<0.0001** |
| L-Aspartic acid | 1272 | 445, 2025 | 1969 | 1616, 2123 | 0.26 | 1846 | 1220, 2093 | 2029 | 1778, 2393 | 0.25 |
| L-Asparagine | 76 | 13, 98 | 59 | 1, 93 | 0.60 | **55** | **2, 88** | **4** | **0, 17** | **0.03** |
| N-Acetyl-L-aspartic acid | 6057 | 176, 11760 | 3566 | 333, 7081 | 0.57 | 2822 | 979, 5981 | 471 | 381, 676 | 0.07 |
| N-Acetyl-L-asparagine | 119 | 56, 358 | 81 | 11, 138 | 0.88 | 96 | 18, 145 | 12 | 11, 16 | 0.09 |
| L-Glutamic acid | 11375 | 2946, 12640 | 9379 | 3540, 11900 | 0.74 | 12450 | 8205, 13680 | 9081 | 7754, 12300 | 0.53 |
| L-Glutamine | 252 | 195, 317 | 281 | 214, 2044 | 0.13 | **315** | **249, 627** | **5670** | **3820, 5796** | **<0.0001** |
| N-Acetyl glutamic acid | 2 | 1, 4 | 1 | 1, 2 | 0.96 | 2 | 1, 2 | 1 | 1, 1 | 0.38 |
| Glycine | 109 | 14, 143 | 119 | 108, 141 | 0.54 | 132 | 63, 135 | 59 | 56, 63 | 0.14 |
| GABA | 503 | 93, 912 | 962 | 504, 1132 | 0.12 | **1981** | **690, 3502** | **4009** | **3522, 4995** | **0.002** |
| Taurine | 4086 | 4037, 4158 | 4131 | 3842, 4420 | 0.93 | 5121 | 4188, 6330 | 5790 | 5069, 6202 | 0.40 |
| 5-Hydroxyindoleacetic acid | 20 | 16, 35 | 33 | 16, 47 | 0.42 | 21 | 20, 33 | 17 | 16, 28 | 0.63 |
| Kynurenic acid | 169 | 107, 297 | 192 | 15, 311 | 0.98 | 49 | 7, 208 | 1 | 0, 2 | 0.69 |
| Mandelic acid | 55 | 8, 144 | 29 | 8, 52 | 0.84 | 15 | 8, 74 | 2 | 2, 3 | 0.67 |
| 3,(4-hydroxyphenyl)lactated | 229 | 150, 292 | 222 | 196, 248 | 0.72 | 265 | 166, 347 | 157 | 87, 203 | 0.16 |

^1^Significant effects are indicated in bold.

**Supplementary Table 3B.** Neurometabolite signal intensity levels affected by XN in the hippocampus of E4 mice in the standard exposure experiment^1^.

| Standard Exposure | Female |  |  |  |  | Male |  |  |  |  |
| --- | --- | --- | --- | --- | --- | --- | --- | --- | --- | --- |
| Genotype E4 | HFD |  | XN |  |  | HFD |  | XN |  |  |
| Metabolite | Median | P 25, 75 | Median | P 25, P75 | P-value | Median | P 25, 75 | Median | P 25, P75 | P-value |
| L-Arginine | 387 | 330, 467 | 308 | 187, 397 | 0.94 | 283 | 277, 430 | 366 | 313, 524 | 0.93 |
| L-Aspartic acid | 1368 | 1346, 2074 | 1847 | 1743, 1981 | 0.18 | 1578 | 1223, 2028 | 1623 | 1484, 1879 | 0.91 |
| L-Asparagine | 65 | 64, 72 | 67 | 60, 82 | 0.62 | 72 | 69, 78 | 80 | 66, 89 | 0.69 |
| N-Acetyl-L-aspartic acid | 3366 | 1984, 4085 | 3311 | 258, 5311 | 0.67 | 2798 | 260, 60842 | 3137 | 2628, 3742 | 0.62 |
| N-Acetyl-L-asparagine | 70 | 59, 84 | 87 | 70, 104 | 0.92 | 78 | 64, 167 | 88 | 77, 100 | 0.84 |
| L-Glutamic acid | 8554 | 8375,12620 | 9788 | 9055,10800 | 0.55 | 7833 | 6515,10220 | 9575 | 8531,10490 | 0.94 |
| L-Glutamine | 228 | 186, 292 | 181 | 141, 214 | 0.94 | 207 | 190, 213 | 231 | 208, 283 | 0.90 |
| N-Acetyl glutamic acid | 1 | 0, 1 | 1 | 0, 1 | 0.90 | 1 | 1, 1 | 1 | 1, 2 | 0.88 |
| Glycine | 92 | 81, 99 | 122 | 102, 134 | 0.26 | 119 | 107, 162 | 92 | 83, 114 | 0.41 |
| GABA | 378 | 340, 530 | 359 | 340, 556 | 0.94 | 445 | 444, 722 | 441 | 253, 501 | 0.84 |
| Taurine | 3472 | 3070, 3855 | 3322 | 3155, 4086 | 0.86 | 4051 | 3847, 4180 | 3962 | 3610, 3980 | 0.90 |
| 5-Hydroxyindoleacetic acid | 20 | 19, 24 | 22 | 14, 27 | 0.95 | 17 | 14, 24 | 17 | 15, 19 | 0.94 |
| Kynurenic acid | 295 | 126, 631 | 257 | 173, 376 | 0.13 | 224 | 224, 261 | 348 | 118, 441 | 0.27 |
| Mandelic acid | 12 | 10, 19 | 12 | 11, 14 | 0.68 | **43** | **33, 69** | **129** | **56, 252** | **0.04** |
| 3,(4-hydroxyphenyl)lactated | 92 | 83, 190 | 106 | 89, 143 | 0.37 | 133 | 131, 144 | 229 | 118, 308 | 0.11 |

^1^Significant effects are indicated in bold.

**Supplementary Table 3C.** Neurometabolite signal intensity levels affected by XN in the hippocampus of E3 mice in the extended exposure experiment^1^.

| Extended Exposure | Female |  |  |  |  | Male |  |  |  |  |
| --- | --- | --- | --- | --- | --- | --- | --- | --- | --- | --- |
| Genotype E3 | HFD |  | XN |  |  | HFD |  | XN |  |  |
| Metabolite | Median | P 25, 75 | Median | P 25, P75 | P-value | Median | P 25, 75 | Median | P 25, P75 | P-value |
| L-Arginine | 2376 | 2251, 2502 | 2735 | 2173, 3172 | 0.99 | 1835 | 1753, 2071 | 2627 | 2602, 2707 | 0.47 |
| L-Aspartic acid | 2600 | 2105, 2810 | 2389 | 2123, 2864 | 0.77 | **1959** | **1874, 2175** | **2696** | **2539, 3321** | **0.03** |
| L-Asparagine | 12 | 7, 15 | 10 | 3, 16 | 0.60 | 11 | 7, 11 | 9 | 6, 11 | 0.42 |
| N-Acetyl-L-aspartic acid | 33230 | 31745, 36520 | 30410 | 21870, 34400 | 0.06 | **27890** | **26970, 33190** | **36310** | **34290, 37450** | **0.02** |
| N-Acetyl-L-asparagine | 73 | 54, 104 | 77 | 49, 89 | 0.98 | 100 | 87, 104 | 73 | 69, 95 | 0.82 |
| L-Glutamic acid | 12445 | 10092, 13965 | 9716 | 9318, 13650 | 0.52 | 11550 | 10090, 11640 | 12560 | 11250, 12970 | 0.56 |
| L-Glutamine | **14970** | **12625, 15605** | **13610** | **9634, 14700** | **0.03** | **11360** | **11230, 13110** | **15100** | **14550, 15720** | **0.008** |
| N-Acetylglutamic acid | 670 | 654, 740 | 590 | 0, 770 | 0.07 | **592** | **508, 674** | **845** | **758, 872** | **0.02** |
| Glycine | 78 | 72, 81 | 88 | 68, 102 | 0.51 | 69 | 64, 73 | 97 | 95, 99 | 0.35 |
| GABA | 3695 | 3203, 3895 | 3634 | 2801, 4236 | 0.62 | **2918** | **2819, 3180** | **4534** | **4353, 4582** | **0.02** |
| Taurine | 6940 | 6483, 7230 | 6912 | 6086, 7178 | 0.33 | **6484** | **5905, 7245** | **7386** | **7153, 7422** | **0.05** |
| 5-Hydroxyindoleacetic acid | 76 | 62, 81 | 64 | 40, 72 | 0.09 | 71 | 50, 102 | 81 | 76, 101 | 0.15 |
| Kynurenic acid | 1 | 0, 1 | 1 | 0, 1 | 0.74 | 1 | 0, 1 | 1 | 1, 1 | 0.66 |
| Mandelic acid | 1 | 1, 1 | 1 | 0, 1 | 0.79 | 0 | 0, 1 | 1 | 1, 1 | 0.60 |
| 3,(4-hydroxyphenyl)lactate | 233 | 173, 305 | 208 | 142, 241 | 0.09 | 170 | 158, 200 | 200 | 200, 233 | 0.78 |

^1^Significant effects are indicated in bold.

**Supplementary Table 4A.** Nucleotide metabolite signal intensity levels affected by XN in the hippocampus of E3 mice in the standard exposure experiment^1^.

| Standard Exposure | Female |  |  |  |  | Male |  |  |  |  |
| --- | --- | --- | --- | --- | --- | --- | --- | --- | --- | --- |
| Genotype E3 | HFD |  | XN |  |  | HFD |  | XN |  |  |
| Nucleotide | Median | P 25, 75 | Median | P 25, P75 | P-value | Median | P 25, 75 | Median | P 25, P75 | P-value |
| ADP | 472 | 440, 494 | 394 | 331, 458 | 0.59 | 469 | 433, 770 | 491 | 321, 655 | 0.75 |
| Adenosine 3',5'-DP | 112 | 79, 132 | 90 | 75, 109 | 0.66 | 125 | 109, 218 | 125 | 83, 147 | 0.19 |
| AMP | 674 | 333, 942 | 365 | 296, 386 | 0.11 | **580** | **579, 667** | **134** | **79, 181** | **0.05** |
| 2',3'-cyclic AMP | 39 | 15, 127 | 20 | 0, 118 | 0.27 | 5 | 0, 17 | 0 | 0, 1 | 0.36 |
| 5'-Methylthioadenosine | 3 | 1, 6 | 5 | 2, 21 | 0.27 | 11 | 2, 24 | 18 | 10, 27 | 0.26 |
| N6-(delta2-Isopentenyl)-adenine | 151 | 128, 184 | 117 | 35, 164 | 0.14 | **120** | **54, 145** | **3** | **0, 16** | **0.001** |
| GMP | 266 | 131, 377 | 138 | 49, 179 | 0.08 | **199** | **75, 269** | **36** | **29, 45** | **0.02** |
| 2'-Deoxyguanosine 5'-MP | 659 | 215, 969 | 372 | 64, 731 | 0.29 | **431** | **240, 740** | **66** | **57, 92** | **0.01** |
| Guanine | 22 | 19, 64 | 24 | 12, 54 | 0.79 | 44 | 40, 62 | 49 | 2, 88 | 0.74 |
| IMP | 15 | 5, 24 | 13 | 6, 21 | 0.94 | 30 | 11, 56 | 35 | 25, 52 | 0.72 |
| Inosine | 2 | 1, 5 | 2 | 1, 4 | 0.28 | **4** | **3, 18** | **75** | **33, 117** | **0.001** |
| Hypoxanthine | 626 | 343, 1841 | 973 | 368, 1583 | 0.26 | **1597** | **1145, 1980** | **2988** | **2512, 4489** | **0.006** |
| Xanthine | 2509 | 2186, 2663 | 3057 | 2329, 3158 | 0.48 | **2367** | **1994, 2905** | **3651** | **3035, 4464** | **0.03** |
| Uric acid | 5491 | 3284, 6212 | 3439 | 2232, 5138 | 0.44 | 4102 | 2201, 5912 | 2433 | 1669, 2881 | 0.16 |
| Allantoin | 11 | 6, 17 | 11 | 9, 13 | 0.83 | 10 | 9, 12 | 11 | 8, 12 | 0.71 |
| CMP | 144 | 117, 171 | 100 | 31, 144 | 0.11 | **118** | **100, 153** | **49** | **38, 59** | **0.005** |
| Deoxycytidine | 140 | 23, 196 | 35 | 12, 68 | 0.54 | 23 | 5, 104 | 9 | 6, 12 | 0.21 |
| Cytosine | 1274 | 420, 1439 | 783 | 316, 1307 | 0.25 | **1244** | **514, 1362** | **472** | **318, 901** | **0.05** |
| Thymine | 60 | 31, 173 | 62 | 23, 142 | 0.46 | 27 | 20, 44 | 15 | 7, 38 | 0.67 |
| Uridine 5'-MP | 139 | 85, 200 | 86 | 55, 114 | 0.06 | **148** | **137, 162** | **19** | **4, 79** | **0.0009** |
| Uridine | 93 | 63, 124 | 61 | 35, 206 | 0.43 | **101** | **75, 161** | **1002** | **627, 1163** | **0.0001** |
| UDP-N-acetylglucosamine | 7 | 3, 8 | 6 | 5, 7 | 0.92 | 6 | 4, 9 | 4 | 3, 5 | 0.64 |
| UDP-galactose | 4 | 2, 4 | 2 | 1, 3 | 0.21 | 3 | 3, 4 | 3 | 1, 5 | 0.84 |
| Uracil | 1903 | 709, 2489 | 1807 | 350, 2474 | 0.54 | 1423 | 596, 2261 | 2179 | 1916, 2417 | 0.07 |
| Orotic acid | 587 | 412, 664 | 307 | 21, 365 | 0.84 | 170 | 9, 378 | 8 | 5, 11 | 0.50 |

^1^Significant effects are indicated in bold.

**Supplementary Table 4B.** Nucleotide metabolite signal intensity levels affected by XN in the hippocampus of E4 mice in the standard exposure experiment.

| Standard Exposure | Female |  |  |  |  | Male |  |  |  |  |
| --- | --- | --- | --- | --- | --- | --- | --- | --- | --- | --- |
| Genotype E4 | HFD |  | XN |  |  | HFD |  | XN |  |  |
| Nucleotide | Median | P 25, 75 | Median | P 25, P75 | P-value | Median | P 25, 75 | Median | P 25, P75 | P-value |
| ADP | 304 | 295, 400 | 204 | 183, 411 | 0.60 | 276 | 261, 342 | 336 | 282, 399 | 0.32 |
| Adenosine 3',5'-DP | 97 | 63, 113 | 61 | 50, 88 | 0.36 | 73 | 69, 95 | 54 | 35, 94 | 0.99 |
| AMP | 362 | 313, 413 | 224 | 162, 308 | 0.32 | 393 | 280, 736 | 568 | 461, 1404 | 0.07 |
| 2',3'-cyclic AMP | 76 | 28, 108 | 74 | 63, 126 | 0.67 | 29 | 14, 93 | 31 | 17, 60 | 0.78 |
| 5'-Methylthioadenosine | 2 | 2, 2 | 1 | 1, 2 | 0.97 | 1 | 1, 3 | 2 | 2, 4 | 0.94 |
| N6-(delta2-Isopentenyl)-adenine | 99 | 92, 131 | 113 | 86, 164 | 0.84 | 89 | 84, 103 | 109 | 87, 157 | 0.43 |
| GMP | 124 | 123, 242 | 126 | 113, 155 | 0.61 | 192 | 127, 195 | 226 | 176, 365 | 0.17 |
| 2'-Deoxyguanosine 5'-MP | 620 | 313, 667 | 321 | 304, 472 | 0.60 | 646 | 531, 705 | 291 | 263, 324 | 0.47 |
| Guanine | 42 | 36, 45 | 30 | 26, 40 | 0.58 | 39 | 26, 41 | 32 | 25, 35 | 0.71 |
| IMP | 10 | 9, 14 | 3 | 2, 3 | 0.38 | 8 | 8, 16 | 11 | 7, 15 | 0.90 |
| Inosine | 3 | 2, 3 | 2 | 1, 5 | 0.99 | 1 | 1, 2 | 2 | 1, 2 | 0.99 |
| Hypoxanthine | 857 | 607, 969 | 531 | 347, 732 | 0.72 | 739 | 405, 801 | 498 | 414, 563 | 0.85 |
| Xanthine | 2570 | 1831, 3118 | 1718 | 1365, 2724 | 0.69 | 1597 | 1330, 1663 | 1669 | 1403, 1768 | 0.74 |
| Uric acid | 4265 | 2934, 4454 | 2607 | 2375, 4584 | 0.67 | 3305 | 3209, 4032 | 2828 | 2108, 3449 | 0.94 |
| Allantoin | 7 | 6, 8 | 6 | 5, 9 | 0.73 | 5 | 5, 6 | 6 | 5, 6 | 0.68 |
| CMP | 118 | 95, 150 | 97 | 87, 161 | 0.97 | 113 | 89, 128 | 131 | 110, 165 | 0.36 |
| Deoxycytidine | 304 | 290, 382 | 324 | 270, 398 | 0.51 | 197 | 183, 256 | 285 | 221, 342 | 0.33 |
| Cytosine | 516 | 488, 595 | 583 | 353, 1083 | 0.86 | 939 | 424, 1341 | 876 | 743, 945 | 0.66 |
| Thymine | 28 | 27, 57 | 35 | 23, 47 | 0.93 | 31 | 23, 34 | 23 | 15, 31 | 0.82 |
| Uridine 5'-MP | 104 | 104, 134 | 91 | 70, 129 | 0.63 | 107 | 85, 150 | 150 | 97, 189 | 0.40 |
| Uridine | 77 | 71, 84 | 558 | 46, 66 | 0.83 | 97 | 61, 99 | 63 | 51, 77 | 0.94 |
| UDP-N-acetylglucosamine | 7 | 5, 13 | 7 | 6, 10 | 0.86 | 8 | 7, 11 | 10 | 8, 15 | 0.38 |
| UDP-galactose | 2 | 1, 3 | 1 | 1, 2 | 0.84 | 1 | 1, 2 | 2 | 1, 2 | 0.42 |
| Uracil | 1496 | 1146, 1730 | 1483 | 1283, 2139 | 0.54 | 1511 | 1290, 1748 | 1563 | 1373, 1747 | 0.73 |
| Orotic acid | 213 | 184, 760 | 406 | 177, 834 | 0.77 | 800 | 445, 1094 | 687 | 123, 1869 | 0.66 |

**Supplementary Table 4C.** Nucleotide metabolite signal intensity levels affected by XN in the hippocampus of E3 mice in the extended exposure experiment^1^.

| Extended Exposure | Female |  |  |  |  | Male |  |  |  |  |
| --- | --- | --- | --- | --- | --- | --- | --- | --- | --- | --- |
| Genotype E3 | HFD |  | XN |  |  | HFD |  | XN |  |  |
| Nucleotide | Median | P 25, 75 | Median | P 25, P75 | P-value | Median | P 25, 75 | Median | P 25, P75 | P-value |
| ADP | **5067** | **4578, 5457** | **3706** | **1849, 4459** | **0.001** | **4405** | **4262, 4574** | **5010** | **4837, 5712** | **0.006** |
| Adenosine 3',5'-DP | **1041** | **741, 1182** | **655** | **383, 869** | **0.02** | **803** | **721, 817** | **923** | **883, 1291** | **0.002** |
| AMP | **4386** | **3633, 5476** | **3167** | **2447, 3386** | **0.002** | **3567** | **3058, 5023** | **4791** | **4787, 5020** | **0.04** |
| 2',3'-cyclic AMP | 5 | 4, 6 | 3 | 0, 7 | 0.85 | 7 | 3, 9 | 4 | 4, 5 | 0.88 |
| 5'-Methylthioadenosine | **464** | **398, 477** | **315** | **256, 391** | **0.006** | 396 | 323, 488 | 467 | 422, 467 | 0.14 |
| N6-(delta2-Isopentenyl)-adenine | 0 | 0, 1 | 1 | 0, 1 | 0.43 | 1 | 0, 1 | 1 | 1, 1 | 0.34 |
| GMP | **1857** | **1428, 2246** | **1072** | **374, 1526** | **0.0004** | 1558 | 1240, 1954 | 1582 | 1299, 1927 | 0.87 |
| 2'-Deoxyguanosine 5'-MP | 131 | 103, 138 | 105 | 76, 173 | 0.87 | 117 | 71, 132 | 138 | 128, 153 | 0.97 |
| Guanine | 432 | 1, 880 | 33 | 1, 841 | 0.65 | 872 | 664, 963 | 2 | 0, 1043 | 0.25 |
| IMP | **4707** | **3995, 5518** | **2244** | **1128, 2858** | **0.0001** | 3404 | 3116, 5500 | 5138 | 3628, 5610 | 0.15 |
| Inosine | 30865 | 25330, 34500 | 25720 | 4157, 33540 | 0.06 | 29870 | 24160, 33080 | 32740 | 30710, 36700 | 0.39 |
| Hypoxanthine | 4603 | 3557, 6210 | 3302 | 2246, 6078 | 0.64 | **2881** | **1380, 3272** | **7226** | **6358, 7391** | **0.002** |
| Xanthine | 2911 | 1114, 4750 | 1865 | 1143, 5867 | 0.88 | 1842 | 1045, 3529 | 5876 | 4692, 5978 | 0.08 |
| Uric acid | 138 | 131, 155 | 164 | 128, 499 | 0.65 | 135 | 115, 152 | 160 | 14, 1638 | 0.55 |
| Allantoin | 14 | 12, 14 | 14 | 11, 15 | 0.70 | 13 | 11, 14 | 13 | 13, 14 | 0.71 |
| CMP | 391 | 379, 433 | 379 | 280, 415 | 0.07 | **340** | **330, 365** | **414** | **395, 426** | **0.04** |
| Deoxycytidine | 9 | 7, 11 | 12 | 8, 13 | 0.40 | 8 | 8, 11 | 13 | 13, 14 | 0.62 |
| Cytosine | 128 | 111, 168 | 168 | 126, 211 | 0.61 | 113 | 113, 145 | 176 | 144, 180 | 0.73 |
| Thymine | 1 | 0, 2 | 1 | 0, 2 | 0.80 | 1 | 1, 1 | 1 | 1, 1 | 0.78 |
| Uridine 5'-MP | **671** | **553, 760** | **443** | **306, 630** | **0.006** | 522 | 509, 567 | 643 | 597, 668 | 0.09 |
| Uridine | 5237 | 4825, 5755 | 5288 | 3847, 5672 | 0.18 | 5356 | 4220, 5800 | 5518 | 5145, 5917 | 0.10 |
| UDP-N-acetylglucosamine | **845** | **798, 943** | **748** | **673, 853** | **0.04** | 773 | 721, 773 | 895 | 711, 902 | 0.07 |
| UDP-galactose | 316 | 262, 333 | 287 | 227, 377 | 0.37 | 304 | 250, 327 | 269 | 257, 352 | 0.35 |
| Uracil | 370 | 319, 495 | 502 | 317, 566 | 0.65 | 276 | 254, 451 | 546 | 541, 552 | 0.74 |
| Orotic acid | 13 | 10, 17 | 10 | 7, 16 | 0.90 | 10 | 7, 16 | 12 | 12, 13 | 0.90 |

^1^Significant effects are indicated in bold.

**Supplementary Table 5A.** Peptide and amino acid metabolite signal intensities affected by XN in the hippocampus of E3 mice in the standard exposure experiment^1,2^.

| Standard Exposure | Female |  |  |  |  | Male |  |  |  |  |
| --- | --- | --- | --- | --- | --- | --- | --- | --- | --- | --- |
| Genotype E3 | HFD |  | XN |  |  | HFD |  | XN |  |  |
| Amino Acid/ Peptide | Median | P 25, 75 | Median | P 25, P75 | P-value | Median | P 25, 75 | Median | P 25, P75 | P-value |
| L-Alanine | 1214 | 1115,1319 | 1089 | 1026, 1150 | 0.88 | 1425 | 1185, 1616 | 1449 | 1170, 1793 | 0.96 |
| Histidine | 1950 | 215, 2445 | 1797 | 341, 2370 | 0.88 | 2215 | 1208, 2516 | 1178 | 1033, 1493 | 0.17 |
| 3-Methylhistidine | 1580 | 1356, 2368 | 1588 | 340, 1980 | 0.28 | 1099 | 627, 1522 | 535 | 377, 645 | 0.07 |
| Urocanic acid | 6449 | 555, 11400 | 1639 | 294, 9443 | 0.45 | 2277 | 499, 8159 | 12 | 9, 16 | 0.11 |
| Leucine | 39520 | 6168,49100 | 34815 | 29420,46170 | 0.77 | 36285 | 22480,47110 | 19605 | 18090,22500 | 0.14 |
| N-Acetyl leucine | 435 | 244, 587 | 509 | 356, 679 | 0.47 | 385 | 192, 557 | 313 | 162, 357 | 0.42 |
| Lysine | 16495 | 1490,20170 | 11107 | 1016,18690 | 0.53 | **14540** | **6267,18790** | **5500** | **5024, 7019** | **0.05** |
| N6,N6,N6-Trimethyl-lysine | 3410 | 2983, 3748 | 2898 | 1050,3559 | 0.19 | **2736** | **1585, 4054** | **1144** | **971, 1254** | **0.005** |
| 5-Aminopentanoic acid | 7734 | 1437, 10050 | 7690 | 4146, 8844 | 0.96 | 7188 | 3893,10140 | 3218 | 2980, 3953 | 0.07 |
| Aminoadipic acid/ methyl-L-glutarate | 505 | 388, 652 | 488 | 378, 570 | 0.73 | 556 | 509, 572 | 422 | 349, 545 | 0.63 |
| Pipecolic acid | 20 | 12, 31 | 12 | 1, 29 | 0.30 | 6 | 0, 12 | 1 | 1, 1 | 0.28 |
| Methionine | 21850 | 6298,31850 | 18685 | 6502,28900 | 0.59 | **21245** | **6439,30140** | **4457** | **4322, 5848** | **0.007** |
| N-Acetyl-methionine | 1277 | 345, 2404 | 693 | 156, 1480 | 0.32 | **505** | **156, 2087** | **130** | **61, 145** | **0.04** |
| L-Aminocyclo-propane-carboxylate | 1547 | 519, 1921 | 1213 | 749, 1674 | 0.55 | **1326** | **940, 1821** | **749** | **660, 877** | **0.05** |
| Phenylalanine | 42040 | 6953,100900 | 46865 | 41740,69100 | 0.93 | 43890 | 2898,82500 | 26370 | 23090,34080 | 0.14 |
| N-Acetyl-phenylalanine | 425 | 269, 548 | 402 | 382, 587 | 0.87 | **280** | **223, 607** | **142** | **102, 186** | **0.03** |
| Proline | 12335 | 839, 14840 | 10791 | 1976, 13700 | 0.97 | 8735 | 5752,14680 | 7187 | 5284, 7606 | 0.50 |
| 5-oxo-proline | 635 | 76, 760 | 596 | 302, 937 | 0.49 | 316 | 214, 585 | 421 | 273, 541 | 0.52 |
| 4-guanidino-butonoate | 165 | 60, 305 | 118 | 55, 313 | 0.14 | 92 | 67, 133 | 67 | 47, 112 | 0.96 |
| Serine | 28 | 14, 43 | 37 | 37, 44 | 0.37 | **37** | **16, 51** | **127** | **59, 150** | **0.0001** |
| N-Acetylserine | 475 | 352, 524 | 454 | 87, 492 | 0.41 | **327** | **123, 490** | **59** | **43, 77** | **0.006** |
| Threonine | 3922 | 1737, 5303 | 4016 | 1310, 4486 | 0.54 | **3281** | **975, 4972** | **982** | **973, 1002** | **0.02** |
| Tryptophan | 9165 | 2475,29900 | 13545 | 3930, 18800 | 0.60 | 11539 | 6547,18890 | 3560 | 3352, 4883 | 0.11 |
| Tyrosine | 35835 | 8466,46450 | 31960 | 19300,44140 | 0.91 | **33295** | **14380,49490** | **12060** | **10120,14250** | **0.03** |
| 3,4-dihydroxyphenyl-acetate | 33 | 4, 398 | 10 | 0, 46 | 0.75 | 9 | 2, 19 | 1 | 0, 1 | 0.83 |
| Valine | 1806 | 157, 2232 | 1502 | 401, 1953 | 0.78 | 1330 | 552, 1972 | 437 | 411, 603 | 0.06 |
| Creatine | 53725 | 45270,60450 | 49295 | 47370,51200 | 0.92 | 60305 | 54040,70230 | 63260 | 49670,72740 | 0.96 |
| Creatinine | 820 | 450, 942 | 779 | 638, 818 | 0.32 | 1804 | 675, 2707 | 2105 | 1642, 2638 | 0.17 |
| Betaine | 50625 | 49080,51580 | 44750 | 27150,51320 | 0.22 | **55625** | **37760,63240** | **1365** | **568, 2318** | **0.0001** |
| Carnosine | 79 | 25, 91 | 55 | 34, 60 | 0.11 | 98 | 56, 120 | 67 | 53, 83 | 0.66 |
| Glutathione | 65 | 35, 87 | 40 | 5, 74 | 0.65 | 83 | 60, 127 | 29 | 27, 41 | 0.06 |

^1^Significant effects are indicated in bold.

^2^Signal intensities were divided by 1000. C: carbon, Cer: ceramide, SM: sphingomyelin, Hex2Cer: dihexose-glucose, Hex1Cer: monohexose glucose, Glyc.: glycosylated ceramides.

**Supplementary Table 5B.** Peptide and amino acid metabolite signal intensities affected by XN in the hippocampus of E4 mice in the standard exposure experiment^1^.

| Standard Exposure | Female |  |  |  |  | Male |  |  |  |  |
| --- | --- | --- | --- | --- | --- | --- | --- | --- | --- | --- |
| Genotype E4 | HFD |  | XN |  |  | HFD |  | XN |  |  |
| Amino Acid/Protein | Median | P 25, 75 | Median | P 25, P75 | P-value | Median | P 25, 75 | Median | P 25, P75 | P-value |
| Alanine | 927 | 843, 1149 | 737 | 694, 1071 | 0.20 | 860 | 784, 934 | 1066 | 890, 1192 | 0.36 |
| Histidine | 1589 | 1510, 2675 | 1644 | 1451, 1777 | 0.55 | 1626 | 991, 2133 | 1448 | 1373, 1629 | 0.65 |
| 3-Methylhistidine | 1377 | 1214, 1933 | 1265 | 1231, 1771 | 0.21 | 1212 | 1089, 1578 | 1598 | 1357, 2056 | 0.39 |
| Urocanic acid | 8353 | 7462, 9248 | 7399 | 5546, 10720 | 0.78 | 6031 | 4646, 6374 | 7320 | 6591, 8622 | 0.48 |
| Leucine | 33700 | 28460,39280 | 34265 | 29650,39460 | 0.85 | 29930 | 23870,44710 | 33075 | 32510,34960 | 0.45 |
| N-Acetylleucine | 271 | 184, 395 | 217 | 176, 264 | 0.53 | 196 | 146, 275 | 235 | 175, 350 | 0.74 |
| Lysine | 12940 | 10990,14230 | 13880 | 12340,15670 | 0.41 | 13380 | 9503,19270 | 10900 | 9278,11560 | 0.48 |
| N6,N6,N6-Trimethyl-lysine | 3344 | 3088, 3390 | 2759 | 2633, 3859 | 0.20 | 2393 | 2282, 3139 | 3316 | 3024, 4063 | 0.58 |
| 5-Aminopentanoate | 7408 | 6097, 7899 | 6749 | 6458, 9035 | 0.78 | 7035 | 5905, 8662 | 7277 | 6863, 7707 | 0.45 |
| Aminoadipic acid/ methyl-L-glutarate | 434 | 389, 507 | 314 | 294, 499 | 0.20 | 395 | 358, 398 | 449 | 353, 646 | 0.67 |
| Pipecolic acid | 43 | 16, 43 | 30 | 29, 41 | 0.56 | 17 | 15, 26 | 22 | 14, 32 | 0.50 |
| Methionine | 20000 | 15920,20980 | 18730 | 16300,27730 | 0.70 | 17200 | 16220,25760 | 20770 | 19520,24120 | 0.48 |
| N-Acetyl-methionine | 341 | 330, 416 | 454 | 390, 575 | 0.91 | 492 | 268, 787 | 631 | 474, 767 | 0.74 |
| Phenylalanine | 1321 | 1167, 1412 | 1330 | 1230, 1748 | 0.88 | 57780 | 56730,77120 | 54480 | 50440,67320 | 0.85 |
| N-Acetyl-phenylalanine | 69530 | 58220,71330 | 63785 | 51130,82510 | 0.78 | 232 | 220, 309 | 251 | 241, 308 | 0.43 |
| Proline | 7478 | 6603, 11740 | 7083 | 6828 | 0.86 | 6597 | 3462, 12560 | 4939 | 4420, 6223 | 0.44 |
| 5-oxo-proline | 209 | 174, 283 | 291 | 64, 9747 | 0.98 | 196 | 185, 366 | 122 | 109, 260 | 0.59 |
| 4-guanidino-butonoate | 53 | 27, 165 | 31 | 22, 349 | 0.59 | 97 | 57, 148 | 133 | 45, 386 | 0.58 |
| Serine | 23 | 20, 32 | 27 | 22, 42 | 0.85 | 25 | 21, 34 | 27 | 23, 29 | 0.93 |
| N-Acetylserine | 420 | 259, 438 | 299 | 256, 36 | 0.67 | 301 | 230, 353 | 322 | 296, 466 | 0.38 |
| Threonine | 3212 | 3076, .813 | 3440 | 3180, 430 | 0.58 | 3868 | 3570, 4420 | 3920 | 3072, 4912 | 0.75 |
| Tryptophan | 14320 | 9848, 15350 | 10911 | 8279, 4779 | 0.68 | 16210 | 12580,16490 | 11420 | 4060, 15860 | 0.39 |
| Tyrosine | 30800 | 24270,31120 | 29630 | 26710,25610 | 0.65 | 28340 | 26490,41570 | 33600 | 30210,41530 | 0.74 |
| 3,4-dihydroxyphenyl-acetate | 150 | 58, 362 | 259 | 167, 306 | 0.81 | **211** | **78, 269** | **545** | **73, 1169** | **0.009** |
| Valine | 1289 | 1138, 1663 | 1431 | 1294, 1679 | 0.54 | 1170 | 1114, 1659 | 1276 | 1184, 1350 | 0.89 |
| Creatine | 40850 | 39850,52100 | 37575 | 34190,51480 | 0.34 | 41420 | 37080,45240 | 52035 | 44840,55880 | 0.15 |
| Creatinine | 467 | 281, 503 | 345 | 251, 519 | 0.92 | 482 | 455, 644 | 558 | 453, 634 | 0.91 |
| Betaine | 36840 | 36210,51650 | 35775 | 31390,39530 | 0.55 | 47110 | 38980,48640 | 42605 | 33340,53390 | 0.92 |
| Carnosine | 26 | 25, 43 | 26 | 13, 43 | 0.78 | 56 | 38, 87 | 52 | 43, 61 | 0.49 |
| Glutathione | 14 | 10, 15 | 18 | 13, 31 | 0.21 | 38 | 14, 132 | 21 | 10, 59 | 0.56 |

^1^Significant effects are indicated in bold.

**Supplementary Table 5C.** Peptide and amino acid metabolite signal intensities affected by XN in the hippocampus of E3 mice in the extended exposure experiment^1,2^.

| Standard Exposure | Female |  |  |  |  | Male |  |  |  |  |
| --- | --- | --- | --- | --- | --- | --- | --- | --- | --- | --- |
| Genotype E3 | HFD |  | XN |  |  | HFD |  | XN |  |  |
| Amino Acid/Peptide | Median | P 25, 75 | Median | P 25, P75 | P-value | Median | P 25, 75 | Median | P 25, P75 | P-value |
| L-Alanine | 1732 | 1515, 1871 | 1742 | 1290, 1946 | 0.36 | **1436** | **1391, 1543** | **1983** | **1915, 1986** | **0.003** |
| Histidine | 521 | 486, 582 | 540 | 487, 592 | 0.65 | 502 | 455, 590 | 701 | 693, 720 | 0.94 |
| 3-Methylhistidine | 137 | 97, 158 | 190 | 116, 266 | 0.39 | 238 | 233, 403 | 315 | 305, 382 | 0.70 |
| Urocanic acid | 34 | 24, 43 | 37 | 23, 57 | 0.48 | 30 | 23, 31 | 38 | 28, 52 | 0.50 |
| Leucine | 3523 | 2921, 3652 | 3812 | 3188, 4601 | 0.45 | 2952 | 2877, 3373 | 4653 | 4458, 4877 | 0.60 |
| N-Acetyl leucine | 7 | 6, 9 | 7 | 7, 8 | 0.74 | 7 | 6, 8 | 8 | 8, 9 | 0.77 |
| Lysine | 831 | 799, 878 | 992 | 816, 1072 | 0.48 | 751 | 714, 889 | 998 | 981, 1017 | 0.51 |
| N6,N6,N6-Trimethyl-lysine | 1322 | 1153, 1501 | 1466 | 1096, 1500 | 0.58 | 1188 | 1061, 1208 | 1616 | 1585, 1663 | 0.77 |
| 5-Aminopentanoic acid | 554 | 478, 571 | 585 | 526, 645 | 0.45 | 500 | 444, 507 | 673 | 633, 731 | 0.52 |
| Aminoadipic acid/ methyl-L-glutarate | 661 | 433, 703 | 553 | 404, 631 | 0.67 | **461** | **458, 470** | **739** | **664, 760** | **0.03** |
| Pipecolic acid | 1 | 0, 1 | 0 | 0, 1 | 0.50 | 1 | 0, 1 | 1 | 0, 1 | 0.54 |
| Methionine | 1322 | 1230, 1404 | 1484 | 1126, 1615 | 0.48 | 1072 | 1066, 1262 | 1659 | 1510, 1724 | 0.49 |
| N-Acetyl-methionine | 167 | 151, 194 | 134 | 126, 235 | 0.74 | 198 | 190, 207 | 176 | 175, 208 | 0.67 |
| L-Aminocyclo-propane-carboxylate | 804 | 715, 918 | 668 | 633. 926 | 0.85 | 752 | 738, 801 | 840 | 739, 857 | 0.80 |
| Phenylalanine | 2828 | 1150, 3156 | 3071 | 2928, 4235 | 0.43 | 3100 | 2783, 3305 | 2958 | 2917, 3011 | 0.66 |
| N-Acetyl-phenylalanine | 10 | 9, 13 | 10 | 9, 12 | 0.76 | 11 | 10, 11 | 10 | 10, 11 | 0.74 |
| Proline | 1332 | 1169, 1415 | 1568 | 1145, 1868 | 0.96 | 1051 | 913, 1246 | 1817 | 1815, 1822 | 0.80 |
| 5-oxo-proline | 163 | 83, 264 | 235 | 90, 322 | 0.99 | 78 | 64, 172 | 220 | 173, 391 | 0.34 |
| 4-guanidino-butonoate | 60 | 51, 70 | 70 | 45, 74 | 0.99 | 51 | 51, 53 | 72 | 70, 74 | 0.98 |
| Serine | 49 | 47, 51 | 51 | 35, 59 | 0.95 | 37 | 35, 47 | 54 | 53, 54 | 0.61 |
| N-Acetylserine | 13 | 9, 15 | 10 | 6, 13 | 0.95 | 10 | 8, 15 | 16 | 15, 18 | 0.95 |
| Threonine | 300 | 282, 320 | 322 | 254, 341 | 0.99 | 256 | 236, 276 | 323 | 320, 345 | 0.92 |
| Tryptophan | 841 | 623, 935 | 987 | 899, 1186 | 0.99 | 508 | 380, 554 | 982 | 924, 1194 | 0.89 |
| Tyrosine | 2782 | 2331, 4024 | 2934 | 2227, 3222 | 0.95 | 2065 | 2020, 2768 | 3226 | 3066, 3591 | 0.90 |
| 3,4-dihydroxyphenyl-acetate | 0 | 0, 1 | 0 | 0, 1 | 0.99 | 1 | 0, 1 | 0 | 0, 0 | 0.99 |
| Valine | 30 | 16, 41 | 47 | 35, 53 | 0.96 | 21 | 15, 26 | 35 | 30, 46 | 0.95 |
| Creatine | 72430 | 68320,79160 | 72300 | 57080,80000 | 0.58 | **66190** | **61660,68130** | **79760** | **79360,84910** | **0.02** |
| Creatinine | 1353 | 1005, 1530 | 1187 | 729, 1561 | 0.85 | 929 | 769, 984 | 1571 | 1564, 1756 | 0.17 |
| Betaine | 1269 | 884, 1425 | 1342 | 1148, 1624 | 0.97 | 896 | 885, 1282 | 1319 | 1057, 1546 | 0.90 |
| Carnosine | 283 | 262, 336 | 274 | 232, 324 | 0.40 | 226 | 221, 283 | 319 | 305, 335 | 0.09 |
| Glutathione | 1073 | 649, 1300 | 681 | 603, 3947 | 0.93 | 1170 | 1164, 3843 | 1227 | 1145, 1535 | 0.35 |

^1^Significant effects are indicated in bold.

^2^Signal intensities were divided by 1000. C: carbon, Cer: ceramide, SM: sphingomyelin, Hex2Cer: dihexose-glucose, Hex1Cer: monohexose glucose, Glyc.: glycosylated ceramides.

**Supplementary Table 6A.** Lipid metabolite signal intensities affected by XN in the hippocampus of E3 mice in the standard exposure experiment^1,2^.

| Standard Exposure | Female |  |  |  |  | Male |  |  |  |  |
| --- | --- | --- | --- | --- | --- | --- | --- | --- | --- | --- |
| Genotype E3 | HFD |  | XN |  |  | HFD |  | XN |  |  |
| Lipids | Median | P 25, 75 | Median | P 25, P75 | P-value | Median | P 25, 75 | Median | P 25, P75 | P-value |
| Hydroxyisobutyrate | 1 | 1, 2 | 1 | 0, 6 | 0.69 | 1 | 0, 10 | 2 | 1, 4 | 0.58 |
| Pimelic acid | 85 | 70, 113 | 89 | 64, 94 | 0.62 | 77 | 72, 106 | 81 | 60, 102 | 0.61 |
| Suberic acid | 64 | 63, 73 | 60 | 53, 75 | 0.90 | 71 | 47, 81 | 76 | 61, 88 | 0.94 |
| Azelaic acid | 289 | 272, 330 | 276 | 228, 331 | 0.81 | 289 | 248, 311 | 336 | 294, 366 | 0.78 |
| Dodecanoic acid | 57 | 49, 84 | 46 | 13, 112 | 0.79 | **53** | **24, 70** | **12** | **11, 17** | **0.03** |
| Myristic acid | 168 | 141, 273 | 172 | 145, 313 | 0.66 | **214** | **165, 305** | **135** | **115, 184** | **0.05** |
| Palmitic acid | 5476 | 4102, 5868 | 6608 | 5236, 8470 | 0.13 | 7623 | 5626, 9420 | 5187 | 4564, 5928 | 0.16 |
| Palmitoleic acid | 5453 | 5222, 6915 | 4505 | 1849, 9257 | 0.69 | **4057** | **2242, 4737** | **1288** | **1210, 1747** | **0.04** |
| Heptadecanoic acid | 50 | 35, 60 | 50 | 38, 60 | 0.42 | 51 | 41, 61 | 32 | 28, 48 | 0.22 |
| Octadecenoic acid | 17830 | 13700,24300 | 29885 | 11410,36080 | 0.14 | **18610** | **14790,26650** | **10595** | **9574,11940** | **0.02** |
| Glyceric acid | 259 | 172, 359 | 283 | 201, 391 | 0.18 | **486** | **239, 693** | **1428** | **117, 15950** | **0.0001** |
| Glycerol 2-phosphate | 51 | 21, 74 | 37 | 5, 72 | 0.70 | 15 | 4, 53 | 4 | 3, 5 | 0.23 |
| 3-Phosphoglyceric acid | 17 | 11, 32 | 23 | 3 27 | 0.29 | 20 | 16, 29 | 4 | 2, 6 | 0.54 |
| L-Oleoyl-Rac-Glycerol | 783 | 541, 1721 | 585 | 129, 1624 | 0.94 | 931 | 161, 2046 | 109 | 80, 141 | 0.10 |
| Ethanolamine phosphate | 10 | 3, 11 | 10 | 5, 21 | 0.95 | 35 | 13, 64 | 7 | 5, 8 | 0.49 |
| Phosphocholine chloride | 230 | 212, 282 | 304 | 121, 732 | 0.74 | 2164 | 323, 3536 | 1976 | 741, 2759 | 0.55 |
| Sphinganine | 377 | 333, 410 | 347 | 234, 394 | 0.90 | 281 | 251, 343 | 369 | 285, 497 | 0.28 |
| Sphingomyelin | 37 | 23, 118 | 36 | 26, 41 | 0.84 | 44 | 22, 171 | 62 | 47, 179 | 0.70 |
| Desmosterol | 2717 | 1965, 3494 | 2616 | 206, 2999 | 0.57 | 3166 | 1960, 4534 | 2512 | 228, 28050 | 0.29 |

^1^Significant effects are indicated in bold.

^2^Signal intensities were divided by 1000. C: carbon, Cer: ceramide, SM: sphingomyelin, Hex2Cer: dihexose-glucose, Hex1Cer: monohexose glucose, Glyc.: glycosylated ceramides.

**Supplementary Table 6B.** Lipid metabolite signal intensities affected by XN in the hippocampus of E4 mice in the standard exposure experiment^1^.

| Standard Exposure | Female |  |  |  |  | Male |  |  |  |  |
| --- | --- | --- | --- | --- | --- | --- | --- | --- | --- | --- |
| Genotype E4 | HFD |  | XN |  |  | HFD |  | XN |  |  |
| Lipids | Median | P 25, 75 | Median | P 25, P75 | P-value | Median | P 25, 75 | Median | P 25, P75 | P-value |
| Hydroxyisobutyrate | 4 | 1, 12 | 0 | 0, 0 | 0.08 | 1 | 0, 8 | 1 | 1, 3 | 0.88 |
| Pimelic acid | 44 | 44, 78 | 40 | 26, 53 | 0.82 | 39 | 28, 67 | 54 | 46, 63 | 0.45 |
| Suberic acid | 51 | 36, 51 | 41 | 36, 45 | 0.84 | 41 | 34, 42 | 49 | 44, 61 | 0.25 |
| Azelaic acid | 224 | 161, 244 | 196 | 162, 227 | 0.78 | 178 | 152, 188 | 215 | 191, 257 | 0.28 |
| Dodecanoic acid | 47 | 42, 88 | 64 | 57, 72 | 0.79 | 41 | 24, 63 | 47 | 43, 55 | 0.81 |
| Myristic acid | 191 | 162, 207 | 204 | 144, 308 | 0.42 | 210 | 182, 214 | 218 | 165, 276 | 0.60 |
| Palmitic acid | 5638 | 5235, 5741 | 8864 | 6006, 13880 | 0.09 | 8980 | 6848, 9588 | 7574 | 6901,10810 | 0.92 |
| Palmitoleic acid | 5683 | 4556, 5761 | 5277 | 5167, 7313 | 0.71 | 5240 | 4817, 5711 | 5921 | 5461, 6596 | 0.67 |
| Heptadecanoic acid | 46 | 46, 47 | 65 | 44, 111 | 0.07 | 73 | 46, 73 | 66 | 56, 69 | 0.67 |
| Octadecenoic acid | 21960 | 21690,29830 | 26025 | 21800,33060 | 0.38 | 27790 | 27520,31880 | 30030 | 23790,31230 | 0.94 |
| Glyceric acid | 137 | 123, 177 | 132 | 95, 190 | 0.99 | 114 | 105, 207 | 139 | 130, 167 | 0.97 |
| Glycerol 2-phosphate | 55 | 15, 96 | 94 | 83, 116 | 0.06 | 93 | 65, 114 | 49 | 35, 99 | 0.19 |
| 3-Phosphoglyceric acid | 52 | 33, 56 | 33 | 30, 59 | 0.95 | 41 | 37, 46 | 37 | 27, 168 | 0.66 |
| L-Oleoyl-Rac-Glycerol | 1933 | 912, 2221 | 1711 | 1533, 2420 | 0.91 | 1806 | 1353, 1988 | 1777 | 1503, 2021 | 0.88 |
| Ethanolamine phosphate | 31 | 23, 32 | 20 | 16, 25 | 0.92 | **29** | **27, 57** | **17** | **8, 45** | **0.03** |
| Phosphocholine chloride | 402 | 358, 598 | 273 | 87, 399 | 0.70 | 145 | 142, 737 | 143 | 107, 388 | 0.39 |
| Sphinganine | 503 | 266, 521 | 324 | 293, 466 | 0.38 | **507** | **353, 614** | **318** | **244, 354** | **0.04** |
| Sphingomyelin | 132 | 111, 175 | 165 | 80, 269 | 0.85 | 112 | 94, 132 | 126 | 68, 169 | 0.18 |
| Desmosterol | 2111 | 1587, 2555 | 2576 | 2233, 2763 | 0.43 | 2420 | 2157, 3170 | 3028 | 2958, 3176 | 0.48 |

^1^Significant effects are indicated in bold.

**Supplementary Table 6C.** Lipid metabolite signal intensities affected by XN in the hippocampus of E3 mice in the extended exposure experiment^1,2^.

| Standard Exposure | Female |  |  |  |  | Male |  |  |  |  |
| --- | --- | --- | --- | --- | --- | --- | --- | --- | --- | --- |
| Genotype E3 | HFD |  | XN |  |  | HFD |  | XN |  |  |
| Lipids | Median | P 25, 75 | Median | P 25, P75 | P-value | Median | P 25, 75 | Median | P 25, P75 | P-value |
| Hydroxyisobutyrate | 20 | 1, 38 | 2 | 0, 20 | 0.23 | 23 | 21, 27 | 27 | 14, 34 | 0.70 |
| Pimelic acid | 17 | 14, 19 | 16 | 11, 18 | 0.99 | 13 | 11, 16 | 17 | 17, 19 | 0.95 |
| Suberic acid | 49 | 44, 51 | 46 | 36, 49 | 0.47 | 34 | 33, 38 | 48 | 46, 56 | 0.08 |
| Azelaic acid | 215 | 193, 230 | 196 | 171, 210 | 0.57 | 163 | 158, 178 | 216 | 209, 229 | 0.18 |
| Dodecanoic acid | 15 | 9, 25 | 13 | 10, 22 | 0.97 | 26 | 16, 29 | 13 | 12, 13 | 0.37 |
| Myristic acid | 107 | 70, 191 | 106 | 82, 185 | 0.94 | **229** | **156, 242** | **94** | **80, 102** | **0.02** |
| Palmitic acid | 2504 | 1404,5818 | 4454 | 2448,5885 | 0.55 | **6230** | **3848, 6389** | **2353** | **2207, 2697** | **0.04** |
| Palmitoleic acid | 782 | 388, 1481 | 946 | 616, 1490 | 0.61 | 1732 | 1260,, 2048 | 797 | 533, 902 | 0.22 |
| Heptadecanoic acid | 34 | 16, 56 | 38 | 25, 50 | 0.77 | 59 | 30, 60 | 24 | 21, 26 | 0.06 |
| Octadecenoic acid | 5624 | 3661,9935 | 8018 | 4859,10350 | 0.53 | 10660 | 7049,12710 | 6073 | 4956, 5258 | 0.13 |
| Glyceric acid | 316 | 266, 369 | 305 | 192, 360 | 0.80 | 218 | 209, 356 | 339 | 316, 366 | 0.75 |
| Glycerol 2-phosphate | 52 | 26, 84 | 37 | 30, 52 | 0.38 | 47 | 45, 62 | 48 | 24, 64 | 0.85 |
| 3-Phosphoglyceric acid | 29 | 27, 41 | 50 | 35, 60 | 0.37 | 29 | 28, 33 | 41 | 39, 47 | 0.58 |
| L-Oleoyl-Rac-Glycerol | 466 | 288, 1155 | 751 | 453, 1304 | 0.95 | 1103 | 863, 1363 | 382 | 323, 607 | 0.27 |
| Ethanolamine phosphate | 139 | 124, 165 | 145 | 126, 182 | 0.49 | 120 | 89, 154 | 138 | 135, 140 | 0.13 |
| Phosphocholine chloride | 7309 | 6993,9043 | 8181 | 5003,9492 | 0.35 | **6509** | **5966,7220** | **9573** | **8961,10070** | **0.0009** |
| Sphinganine | 415 | 335, 499 | 385 | 299, 428 | 0.73 | **522** | **462, 541** | **308** | **303, 373** | **0.02** |
| Sphingomyelin | 946 | 776, 1066 | 1030 | 96, 1283 | 0.68 | 811 | 623, 2356 | 741 | 700, 907 | 0.06 |
| Desmosterol | 2747 | 1644,6678 | 2535 | 2409,5213 | 0.66 | **5115** | **4576, 5584** | **2436** | **2212, 3098** | **0.04** |

^1^Significant effects are indicated in bold.

^2^Signal intensities were divided by 1000. C: carbon, Cer: ceramide, SM: sphingomyelin, Hex2Cer: dihexose-glucose, Hex1Cer: monohexose glucose, Glyc.: glycosylated ceramides.

**Supplementary Table 7A.** Carbohydrate metabolite signal intensities affected by XN in the hippocampus of E3 mice in the standard exposure experiment^1,2^.

| Standard Exposure | Female |  |  |  |  | Male |  |  |  |  |
| --- | --- | --- | --- | --- | --- | --- | --- | --- | --- | --- |
| Genotype E3 | HFD |  | XN |  |  | HFD |  | XN |  |  |
| Carbohydrate | Median | P 25, 75 | Median | P 25, P75 | P-value | Median | P 25, 75 | Median | P 25, P75 | P-value |
| Gluconic acid | 7268 | 6201, 7979 | 6394 | 4528, 8449 | 0.42 | **5200** | **1672,6822** | **450** | **340, 537** | **0.0008** |
| Hexose-P_1_ | 13 | 8, 15 | 18 | 15, 22 | 0.68 | 20 | 11, 21 | 22 | 16, 33 | 0.71 |
| Rhamnose | 4 | 3, 12 | 4 | 2, 25 | 0.79 | **18** | **12, 27** | **36** | **34, 43** | **0.0009** |
| Aldopentose | 396 | 297, 520 | 419 | 66, 547 | 0.63 | 221 | 76, 363 | 72 | 66, 96 | 0.10 |
| Aldopentose alcohol | 24 | 15, 27 | 22 | 19, 33 | 0.59 | 50 | 23, 74 | 56 | 44, 64 | 0.42 |
| N-Acetylneuraminate | 10400 | 7212,16590 | 10593 | 6921,13320 | 0.77 | **9825** | **4821,10970** | **15615** | **9328,18650** | **0.04** |
| Citric acid | **1324** | **700, 2014** | **2712** | **1068, 6571** | **0.03** | 2745 | 1634,3507 | 5680 | 3142, 8198 | 0.08 |
| Methylmalonic acid/succinic acid | 2582 | 416, 3220 | 2198 | 1107, 2861 | 0.79 | **2152** | **1119, 2503** | **306** | **94, 413** | **0.005** |
| Fumaric acid | 24 | 3, 40 | 27 | 22, 48 | 0.10 | **41** | **17, 60** | **172** | **170, 264** | **0.005** |
| Malic acid | 1610 | 245, 2834 | 1897 | 737, 2415 | 0.37 | **1775** | **969,1887** | **2593** | **2469, 3409** | **0.01** |
| Citrulline | 13870 | 9651,18010 | 10786 | 4847,15270 | 0.29 | **9363** | **3704,15170** | **2190** | **1884, 3261** | **0.01** |
| Ornithine | 1395 | 332, 1709 | 1129 | 738, 1359 | 0.87 | **1516** | **796, 3897** | **954** | **689, 1266** | **0.03** |
| Spermidine | 1549 | 739, 2247 | 1362 | 388, 1678 | 0.71 | 1553 | 1253, 2304 | 1454 | 1335, 2081 | 0.76 |
| Spermine | 247 | 23, 549 | 164 | 65, 671 | 0.50 | 671 | 170, 975 | 715 | 476, 968 | 0.84 |

^1^Significant effects are indicated in bold.

^2^Signal intensities were divided by 1000.

**Supplementary Table 7B.** Carbohydrate metabolite signal intensities affected by XN in the hippocampus of E4 mice in the standard exposure experiment.

| Standard Exposure | Female |  |  |  |  | Male |  |  |  |  |
| --- | --- | --- | --- | --- | --- | --- | --- | --- | --- | --- |
| Genotype E4 | HFD |  | XN |  |  | HFD |  | XN |  |  |
| Carbohydrate | Median | P 25, 75 | Median | P 25, P75 | P-value | Median | P 25, 75 | Median | P 25, P75 | P-value |
| Gluconic acid | 6465 | 5301, 6510 | 5225 | 4150, 6425 | 0.33 | 4742 | 4361, 5146 | 6664 | 5944, 7414 | 0.10 |
| Hexose-P_1_ | 33 | 27, 36 | 31 | 23, 50 | 0.63 | 30 | 29, 57 | 31 | 22, 55 | 0.18 |
| Rhamnose | 15 | 5, 21 | 12 | 8, 19 | 0.87 | 10 | 7, 10 | 16 | 8, 20 | 0.40 |
| Aldopentose | 304 | 267, 506 | 257 | 253, 268 | 0.48 | 208 | 207, 303 | 235 | 178, 332 | 0.59 |
| Aldopentose alcohol | 22 | 20, 23 | 20 | 18, 23 | 0.91 | 20 | 18, 21 | 22 | 17, 24 | 0.59 |
| N-Acetylneuraminate | 8832 | 8222, 9469 | 6529 | 5048, 9906 | 0.72 | 8184 | 6667, 9587 | 8173 | 6797, 8779 | 0.96 |
| Citric acid | 1691 | 422, 1714 | 331 | 309, 1421 | 0.82 | 486 | 350, 863 | 957 | 367, 2419 | 0.79 |
| Methylmalonic acid/succinic acid | 1679 | 997, 2126 | 1717 | 1368, 1972 | 0.68 | 1262 | 1239, 2441 | 1638 | 1438, 1701 | 0.83 |
| Fumaric acid | 29 | 28, 33 | 20 | 17, 39 | 0.99 | 18 | 11, 18 | 21 | 10, 23 | 0.92 |
| Malic acid | 1068 | 826, 1285 | 940 | 660, 1515 | 0.81 | 632 | 492, 1290 | 718 | 518, 908 | 0.55 |
| Citrulline | 11600 | 10440,13600 | 11980 | 11470,15440 | 0.87 | 12240 | 10130,14490 | 13270 | 11640,15490 | 0.59 |
| Ornithine | 989 | 919, 1069 | 939 | 860, 1236 | 0.78 | 840 | 545, 1238 | 940 | 617, 1333 | 0.84 |
| Spermidine | 879 | 780, 1019 | 664 | 363, 968 | 0.49 | 582 | 478, 587 | 856 | 763, 1083 | 0.30 |
| Spermine | 44 | 25, 57 | 31 | 9, 72 | 0.99 | 33 | 23, 81 | 48 | 19, 122 | 0.93 |

Signal intensities were divided by 1000.

**Supplementary Table 7C.** Carbohydrate metabolite signal intensities affected by XN in the hippocampus of E3 mice in the extended exposure experiment.

| Extended Exposure | Female |  |  |  |  | Male |  |  |  |  |
| --- | --- | --- | --- | --- | --- | --- | --- | --- | --- | --- |
| Genotype E3 | HFD |  | XN |  |  | HFD |  | XN |  |  |
| Carbohydrate | Median | P 25, 75 | Median | P 25, P75 | P-value | Median | P 25, 75 | Median | P 25, P75 | P-value |
| Gluconic acid | 205 | 180, 213 | 196 | 160, 206 | 0.45 | 191 | 184, 192 | 227 | 204, 265 | 0.39 |
| Hexose-P_1_ | 235 | 165, 381 | 240 | 131, 261 | 0.22 | 198 | 167, 230 | 238 | 179, 324 | 0.13 |
| Rhamnose | 43 | 42, 49 | 49 | 38, 51 | 0.75 | 41 | 39, 44 | 48 | 46, 51 | 0.07 |
| Aldopentose | 33 | 20, 43 | 38 | 21, 45 | 0.60 | 36 | 36, 40 | 37 | 34, 43 | 0.64 |
| Aldopentose alcohol | 12 | 11, 13 | 12 | 10, 13 | 0.91 | 10 | 9, 13 | 11 | 10, 12 | 0.79 |
| N-Acetylneuraminate | 2256 | 2015, 2349 | 2354 | 2286, 2668 | 0.73 | 2075 | 1971, 2271 | 2528 | 2490, 2591 | 0.77 |
| Citric acid | 9129 | 8970,10420 | 9825 | 7214,10790 | 0.59 | 7026 | 6907,9455 | 10470 | 10250,10780 | 0.08 |
| Methylmalonic acid/succinic acid | 537 | 235, 844 | 316 | 179, 438 | 0.97 | 505 | 406, 728 | 556 | 320, 742 | 0.72 |
| Fumaric acid | 314 | 294, 332 | 285 | 198, 329 | 0.32 | 253 | 245, 294 | 328 | 318, 355 | 0.11 |
| Malic acid | 3008 | 2876, 3168 | 3006 | 1986, 3365 | 0.49 | 2456 | 2418, 3146 | 3391 | 3229, 3506 | 0.18 |
| Citrulline | 129 | 115, 150 | 162 | 137, 215 | 0.43 | 113 | 109, 121 | 154 | 151, 193 | 0.46 |
| Ornithine | 13 | 11, 15 | 16 | 13, 19 | 0.65 | 13 | 12, 14 | 16 | 16, 16 | 0.52 |
| Spermidine | 542 | 466, 755 | 632 | 570, 843 | 0.85 | 703 | 555, 868 | 894 | 788, 1013 | 0.88 |
| Spermine | 57 | 33, 115 | 86 | 66, 87 | 0.98 | 90 | 46, 221 | 131 | 99, 180 | 0.98 |

Signal intensities were divided by 1000.

**Supplementary Table 8A.** Vitamin metabolite signal intensities affected by XN in the hippocampus of E3 mice in the standard exposure experiment^1,2^.

| Standard Exposure | Female |  |  |  |  | Male |  |  |  |  |
| --- | --- | --- | --- | --- | --- | --- | --- | --- | --- | --- |
| Genotype E3 | HFD |  | XN |  |  | HFD |  | XN |  |  |
| Vitamins | Median | P 25, 75 | Median | P 25, P75 | P-value | Median | P 25, 75 | Median | P 25, P75 | P-value |
| p-Aminobenzoic acid | 79 | 35, 125 | 111 | 0, 134 | 0.10 | 91 | 2, 114 | 1 | 1, 1 | 0.36 |
| 4-Pyridoxic acid | 587 | 363, 843 | 505 | 113, 601 | 0.12 | **285** | **63, 465** | **17** | **13, 20** | **0.03** |
| Pantothenic acid | 1968 | 1250, 2586 | 1892 | 458, 2168 | 0.23 | 1199 | 847, 1339 | 785 | 602, 1015 | 0.51 |
| FAD | 218 | 158, 299 | 141 | 126, 166 | 0.08 | 192 | 125, 197 | 89 | 61, 138 | 0.15 |
| NAD | 62 | 32, 90 | 57 | 54, 76 | 0.59 | 93 | 67, 186 | 18 | 12, 54 | 0.06 |
| Nicotinamide | 3 | 2, 10 | 9 | 5, 207 | 0.18 | **4** | **2, 16** | **1666** | **649, 1816** | **<0.0001** |
| Nicotinic acid | 13 | 4, 17 | 6 | 6, 7 | 0.43 | 5 | 4, 8 | 0 | 0, 8 | 0.50 |
| Riboflavin | 731 | 232, 753 | 520 | 421, 610 | 0.69 | 498 | 400, 584 | 322 | 235, 470 | 0.35 |
| Lumichrome | 25 | 24, 26 | 25 | 21, 33 | 0.79 | 23 | 20, 25 | 16 | 15, 20 | 0.28 |
| 4-hydroxybenzoic acid | 26 | 20, 35 | 29 | 20, 51 | 0.28 | 18 | 13, 22 | 17 | 16, 20 | 0.94 |
| Protoporphyrin | 414 | 256, 639 | 326 | 5, 430 | 0.14 | 148 | 8, 644 | 2 | 1, 4 | 0.50 |

^1^Significant effects are indicated in bold.

^2^Signal intensities were divided by 1000.

**Supplementary Table 8B**. Vitamin metabolite signal intensities affected by XN in the hippocampus of E4 mice in the standard exposure experiment.

| Standard Exposure | Female |  |  |  |  | Male |  |  |  |  |
| --- | --- | --- | --- | --- | --- | --- | --- | --- | --- | --- |
| Genotype E4 | HFD |  | XN |  |  | HFD |  | XN |  |  |
| Vitamins | Median | P 25, 75 | Median | P 25, P75 | P-value | Median | P 25, 75 | Median | P 25, P75 | P-value |
| p-Aminobenzoic acid | 66 | 61, 82 | 56 | 50, 58 | 0.85 | 56 | 41, 62 | 80 | 54, 103 | 0.67 |
| 4-Pyridoxic acid | 356 | 333, 539 | 226 | 138, 470 | 0.35 | 299 | 239, 323 | 263 | 195, 315 | 0.58 |
| Pantothenic acid | 1336 | 1331, 1933 | 961 | 731, 1873 | 0.48 | 943 | 805, 1000 | 1201 | 748, 2026 | 0.32 |
| FAD | 131 | 121, 243 | 88 | 82, 189 | 0.25 | 126 | 114, 143 | 134 | 101, 192 | 0.37 |
| NAD | 58 | 51, 103 | 42 | 31, 57 | 0.41 | 59 | 56, 63 | 80 | 55, 116 | 0.23 |
| Nicotinamide | 2 | 1, 2 | 2 | 1, 2 | 0.99 | 3 | 3, 3 | 6 | 3, 8 | 0.99 |
| Nicotinic acid | 8 | 5, 8 | 3 | 3, 6 | 0.18 | 3 | 2, 5 | 6 | 4, 12 | 0.76 |
| Riboflavin | 386 | 344, 635 | 283 | 216, 415 | 0.22 | 330 | 327, 355 | 343 | 243, 526 | 0.35 |
| Lumichrome | 20 | 16, 20 | 15 | 14, 16 | 0.34 | 14 | 12, 15 | 16 | 11, 21 | 0.36 |
| 4-hydroxybenzoic acid | 32 | 20, 36 | 23 | 18, 27 | 0.72 | 21 | 12, 34 | 24 | 23, 26 | 0.93 |
| Protoporphyrin | 381 | 324, 1054 | 635 | 410, 733 | 0.40 | 556 | 136, 721 | 471 | 327, 933 | 0.50 |

Signal intensities were divided by 1000.

**Supplementary Table 8C.** Vitamin metabolite signal intensities affected by XN in the hippocampus of E3 mice in the extended exposure experiment^1,2^.

| Standard Exposure | Female |  |  |  |  | Male |  |  |  |  |
| --- | --- | --- | --- | --- | --- | --- | --- | --- | --- | --- |
| Genotype E3 | HFD |  | XN |  |  | HFD |  | XN |  |  |
| Vitamins | Median | P 25, 75 | Median | P 25, P75 | P-value | Median | P 25, 75 | Median | P 25, P75 | P-value |
| p-Aminobenzoic acid | 1 | 0, 0 | 1 | 0, 0 | 0.87 | 0 | 0, 0 | 1 | 1, 1 | 0.81 |
| 4-Pyridoxic acid | 12 | 8, 16 | 12 | 2, 17 | 0.51 | 11 | 10, 13 | 11 | 11, 12 | 0.60 |
| Pantothenic acid | **2716** | **2489, 2818** | **2582** | **1299, 2714** | **0.04** | 1884 | 1321, 2107 | 2188 | 2095, 2364 | 0.20 |
| FAD | **659** | **614, 694** | **582** | **174, 695** | **0.01** | **574** | **498, 742** | **792** | **759, 929** | **0.004** |
| NAD | 219 | 105, 261 | 181 | 62, 210 | 0.29 | **150** | **142, 159** | **246** | **230, 273** | **0.02** |
| Nicotinamide | 3736 | 3122, 5154 | 3098 | 1684, 4098 | 0.31 | **2918** | **2125, 3785** | **4671** | **4602, 5570** | **0.003** |
| Nicotinic acid | 0 | 0, 0 | 0 | 0, 0 | 0.84 | 0 | 0, 0 | 0 | 0, 0 | 0.91 |
| Riboflavin | 46 | 35, 54 | 48 | 39, 64 | 0.70 | 50 | 32, 52 | 55 | 53, 56 | 0.69 |
| Lumichrome | 12 | 9, 16 | 10 | 9, 14 | 0.67 | 16 | 15, 16 | 12 | 12, 13 | 0.56 |
| 4-hydroxybenzoic acid | 16 | 14, 20 | 15 | 12, 16 | 0.75 | 11 | 10, 14 | 14 | 14, 17 | 0.88 |
| Protoporphyrin | 5 | 2, 6 | 4 | 2, 26 | 0.99 | 5 | 4, 6 | 7 | 4, 10 | 0.99 |

^1^Significant effects are indicated in bold.

^2^Signal intensities were divided by 1000.

**Supplementary Table 9A.** Association (Spearman correlations) between open field test and neuro-metabolite levels in the hippocampus in the standard and extended exposure experiment^1,2^.

| Neuro-metabolite | Distance moved 1 | Distance moved 2 | Center duration 1 | Center duration 2 |
| --- | --- | --- | --- | --- |
| L-Arginine | -0.386*** | -0.365** | +0.150 | +0.102 |
| L-Aspartic acid | -0.243* | -0.236* | +0.249* | +0.251* |
| L-Asparagine | +0.332** | +0.302* | -0.363** | -0.257* |
| N-Acetyl-L-aspartic acid | -0.332** | -0.304* | +0.285* | +0.390*** |
| N-Acetyl-L-asparagine | +0.162 | +0.213 | -0.049 | +0.076 |
| L-Glutamic acid | -0.050 | +0.028 | +0.043 | +0.095 |
| L-Glutamine | -0.531*** | -0.526*** | +0.281* | +0.252* |
| N-Acetylglutamic acid | -0.285* | -0.306** | +0.182 | +0.319** |
| Glycine | +0.253* | +0.221 | -0.018 | -0.015 |
| GABA | -0.416*** | -0.360** | +0.226 | +0.199 |
| Taurine | -0.490*** | -0.459*** | +0.352** | +0.336** |
| 5-Hydroxyindoleacetic acid | -0.312** | -0.337** | +0.387*** | +0.452*** |
| Kynurenic acid | +0.470*** | +0.427*** | -0.346** | -0.306** |
| Mandelic acid | +0.370** | +0.394*** | -0.433*** | -0.362** |
| 3,(4-hydroxyphenyl)lactate | -0.068 | -0.047 | +0.087 | +0.220 |

^1^Significant effects are indicated in bold.

^2^Center duration is in seconds, and distance moved is in cm. 0.05 > P > 0.01: *; 0.01 > P > 0.001: **; P < 0.001: ***

**Supplementary Table 9B.** Association (Spearman correlations) between visible platform water maze activity level (1 – 4 refers to trial number) and neuro-metabolite levels in the hippocampus in the standard and extended exposure experiment^1,2^.

| Neuro-metabolite | Latency 1 (sec) | Latency 4 (sec) | Distance moved 1 | Distance moved 4 |
| --- | --- | --- | --- | --- |
| L-Arginine | +0.265* | +0.157 | +0.203 | +0.177 |
| L-Aspartic acid | +0.098 | +0.092 | +0.122 | +0.029 |
| L-Asparagine | -0.316** | -0.341** | -0.250* | -0.349** |
| N-Acetyl-L-aspartic acid | +0.110 | +0.223 | +0.085 | +0.096 |
| N-Acetyl-L-asparagine | -0.150 | -0.121 | -0.123 | -0.186 |
| L-Glutamic acid | +0.083 | -0.027 | +0.128 | -0.131 |
| L-Glutamine | +0.342** | +0.329** | +0.285* | +0.257* |
| N-Acetylglutamic acid | +0.123 | +0.310** | +0.108 | +0.229 |
| Glycine | -0.252* | -0.286* | -0.178 | -0.300* |
| GABA | +0.283* | +0.186 | +0.265* | +0.142 |
| Taurine | +0.294* | +0.387** | +0.255* | +0.343** |
| 5-Hydroxyindoleacetic acid | +0.225 | +0.259* | +0.193 | +0.203 |
| Kynurenic acid | -0.335** | -0.365** | -0.280* | -0.298* |
| Mandelic acid | -0.397*** | -0.347** | -0.345** | -0.287* |
| 3,(4-hydroxyphenyl)lactate | +0.056 | +0.092 | +0.047 | +0.077 |

^1^Significant effects are indicated in bold.

^2^Latency is in seconds, and distance moved is in cm. 0.05 > P > 0.01: *; 0.01 > P > 0.001: **; P < 0.001: ***

**Supplementary Table 9C.** Association (Spearman correlations) between visible platform water maze activity level (1 – 6 refers to trial number) and neuro-metabolite levels in the hippocampus in the standard and extended exposure experiment^1^.

| Neuro-metabolite | Latency 1 (sec) | Latency 2 (sec) | Latency 3 (sec) | Latency 4 (sec) | Latency 5 (sec) | Latency 6 (sec) |
| --- | --- | --- | --- | --- | --- | --- |
| L-Arginine | +0.163 | +0.074 | +0.030 | +0.182 | +0.270* | +0.265* |
| L-Aspartic acid | +0.084 | +0.154 | +0.110 | +0.145 | -0.039 | -0.053 |
| L-Asparagine | -0.252* | -0.155 | -0.001 | -0.186 | -0.275* | -0.186 |
| N-Acetyl-L-aspartic acid | +0.016 | +0.154 | +0.130 | +0.122 | +0.022 | +0.004 |
| N-Acetyl-L-asparagine | -0.103 | +0.000 | -0.006 | -0.048 | -0.149 | -0.132 |
| L-Glutamic acid | +0.014 | +0.047 | -0.008 | +0.113 | -0.079 | -0.066 |
| L-Glutamine | +0.192 | +0.211 | +0.165 | +0.244* | +0.299* | +0.210 |
| N-Acetylglutamic acid | +0.112 | -0.046 | +0.003 | +0.039 | -0.082 | -0.057 |
| Glycine | -0.124 | -0.173 | -0.180 | -0.265* | -0.329** | -0.233 |
| GABA | +0.130 | +0.100 | +0.029 | +0.259* | +0.236* | +0.217 |
| Taurine | +0.197 | +0.215 | +0.119 | +0.186 | +0.261* | +0.290* |
| 5-Hydroxyindoleacetic acid | +0.052 | +0.158 | +0.038 | +0.065 | +0.106 | +0.091 |
| Kynurenic acid | -0.174 | -0.287* | -0.197 | -0.398*** | -0.376** | -0.314** |
| Mandelic acid | -0.183 | -0.249* | -0.137 | -0.319** | -0.305** | -0.164 |
| 3,(4-hydroxyphenyl)lactate | +0.025 | -0.059 | -0.145 | -0.124 | -0.032 | 0.137 |

^1^Significant effects are indicated in bold.

^2^Latency is in seconds. 0.05 > P > 0.01: *; 0.01 > P > 0.001: **; P < 0.001: ***

**Supplementary Table 9D.** Association (Spearman correlations) between fear conditioning (1 – 4 refers to trial number) and neuro-metabolite levels in the hippocampus in the standard exposure experiment^1,2^.

| Neuro-metabolite | Tone freeze 1 | Tone freeze 2 | Tone freeze 3 | Tone freeze 4 | Shock motion 1 | Shock motion 2 | Shock motion 3 | Shock motion 4 | Cued Fear Tone Freeze |
| --- | --- | --- | --- | --- | --- | --- | --- | --- | --- |
| L-Arginine | +0.336* | -0.048 | -0.173 | -0.009 | -0.188 | -0.209 | -0.242 | -0.221 | -0.306* |
| L-Aspartic acid | +0.185 | -0.033 | +0.010 | +0.232 | -0.166 | -0.133 | -0.038 | -0.059 | -0.056 |
| L-Asparagine | -0.318* | +0.074 | +0.096 | +0.155 | +0.121 | +0.273 | +0.242 | +0.239 | +0.204 |
| N-Acetyl-L-aspartic acid | -0.164 | +0.049 | +0.138 | +0.233 | -0.084 | +0.166 | +0.114 | +0.137 | +0.105 |
| N-Acetyl-L-asparagine | -0.270 | +0.133 | +0.179 | +0.220 | -0.014 | +0.284 | +0.148 | +0.252 | +0.149 |
| L-Glutamic acid | +0.058 | +0.013 | -0.009 | +0.174 | -0.252 | -0.094 | -0.138 | -0.038 | -0.025 |
| L-Glutamine | +0.325* | -0.006 | -0.023 | +0.006 | -0.114 | -0.113 | -0.220 | -0.238 | -0.147 |
| N-Acetylglutamic acid | -0.127 | -0.058 | +0.094 | +0.036 | -0.265 | +0.080 | -0.097 | -0.086 | -0.028 |
| Glycine | -0.043 | +0.065 | +0.285 | +0.337* | +0.054 | +0.187 | +0.260 | +0.179 | +0.236 |
| GABA | +0.297* | -0.093 | -0.193 | -0.080 | -0.448** | -0.262 | -0.299* | -0.226 | -0.318* |
| Taurine | +0.244 | +0.013 | -0.172 | -0.080 | -0.190 | -0.259 | -0.297* | -0.255 | -0.238 |
| 5-Hydroxyindoleacetic acid | +0.168 | +0.072 | +0.136 | +0.178 | -0.033 | -0.136 | -0.022 | -0.012 | -0.102 |
| Kynurenic acid | -0.219 | +0.185 | +0.342* | +0.233 | +0.291* | +0.353* | +0.220 | +0.235 | +0.278 |
| Mandelic acid | -0.214 | +0.240 | +0.105 | +0.027 | +0.275 | +0.312* | +0.047 | +0.174 | +0.199 |
| 3,(4-hydroxyphenyl)lactate | +0.014 | +0.198 | +0.033 | -0.075 | -0.037 | +0.069 | -0.203 | -0.061 | -0.125 |

^1^Significant effects are indicated in bold.

^2^Freeze is in seconds, freeze (%) refers to % of time frozen, motion is in cm. 0.05 > P > 0.01: *; 0.01 > P > 0.001: **; P < 0.001: ***

**Supplementary Table 10A.** Association (Spearman correlations) between open field test and nucleotide and their metabolites levels in the hippocampus in the standard and extended exposure experiment^1,2^.

| Nucleotide | Distance moved 1 | Distance moved 2 | Center duration 1 | Center duration 2 |
| --- | --- | --- | --- | --- |
| ADP | -0.454*** | -0.426*** | +0.265 | +0.322** |
| Adenosine 3',5'-DP | -0.450*** | -0.421*** | +0.281 | +0.339** |
| AMP | -0.466*** | -0.488*** | +0.330** | +0.365** |
| 2',3'-cyclic AMP | +0.411*** | +0.405*** | -0.189 | -0.063 |
| 5'-Methylthioadenosine | -0.465*** | -0.517*** | +0.371** | +0.344** |
| N6-(delta2-Isopentenyl)-adenine | +0.458*** | +0.504*** | -0.356** | -0.312** |
| GMP | -0.342** | -0.373** | +0.305** | +0.384** |
| 2'-Deoxyguanosine 5'-MP | +0.349** | +0.318** | -0.353** | -0.320** |
| Guanine | -0.047 | -0.022 | +0.010 | +0.025 |
| IMP | -0.490*** | -0.454*** | +0.352** | +0.351** |
| Inosine | -0.476*** | -0.449*** | +0.260* | +0.334** |
| Hypoxanthine | -0.415*** | -0.352** | +0.316** | +0.322** |
| Xanthine | -0.059 | -0.051 | +0.078 | +0.129 |
| Uric acid | +0.435*** | +0.424*** | -0.420*** | -0.404*** |
| Allantoin | -0.206 | -0.255* | +0.326** | +0.299* |
| CMP | -0.392*** | -0.411*** | +0.315** | +0.385** |
| Deoxycytidine | +0.378** | +0.419*** | -0.256*** | -0.132 |
| Cytosine | +0.385** | +0.501*** | -0.486*** | -0.374** |
| Thymine | +0.551*** | +0.541*** | -0.327** | -0.271* |
| Uridine 5'-MP | -0.421*** | -0.464*** | +0.311** | +0.336** |
| Uridine | -0.534*** | -0.506*** | +0.294* | +0.307** |
| UDP-N-acetylglucosamine | -0.388*** | -0.458*** | +0.414*** | +0.372** |
| UDP-galactose | -0.406*** | -0.360** | +0.285* | +0.335** |
| Uracil | +0.416*** | +0.453*** | -0.264* | -0.247* |
| Orotic acid | +0.452*** | +0.366** | -0.223 | -0.223* |

^1^Significant effects are indicated in bold.

^2^Center duration is in seconds, and distance moved is in cm. 0.05 > P > 0.01: *; 0.01 > P > 0.001: **; P < 0.001: ***

**Supplementary Table 10B.** Association (Spearman correlations) between visible platform water maze activity level (1 – 4 refers to trial number) and nucleotide and their metabolites levels in the hippocampus in the standard and extended exposure experiment^1^.

| Nucleotide | Latency 1 (sec) | Latency 4 (sec) | Distance moved 1 | Distance moved 4 |
| --- | --- | --- | --- | --- |
| ADP | +0.166 | +0.349** | +0.147 | +0.129 |
| Adenosine 3',5'-DP | +0.273* | +0.301* | +0.260* | +0.115 |
| AMP | +0.149 | +0.339** | +0.098 | +0.237* |
| 2',3'-cyclic AMP | -0.293* | -0.307** | -0.223 | -0.025 |
| 5'-Methylthioadenosine | +0.298* | +0.382** | +0.223 | +0.102 |
| N6-(delta2-Isopentenyl)-adenine | -0.425*** | -0.450*** | -0.326** | -0.187 |
| GMP | +0.125 | +0.324** | +0.109 | +0.249* |
| 2'-Deoxyguanosine 5'-MP | -0.235* | -0.247* | -0.185 | -0.029 |
| Guanine | +0.026 | +0.021 | +0.035 | +0.020 |
| IMP | +0.316** | +0.384** | +0.263* | +0.175 |
| Inosine | +0.357** | +0.281* | +0.332** | +0.098 |
| Hypoxanthine | +0.315** | +0.193 | +0.296* | +0.120 |
| Xanthine | +0.072 | -0.122 | +0.082 | -0.178 |
| Uric acid | -0.351** | -0.413*** | -0.274* | -0.171 |
| Allantoin | +0.220 | +0.162 | +0.165 | +0.107 |
| CMP | +0.152 | +0.266* | +0.105 | +0.232 |
| Deoxycytidine | -0.396*** | -0.303* | -0.302* | +0.081 |
| Cytosine | -0.241* | -0.338** | -0.165 | -0.268* |
| Thymine | -0.269* | -0.440*** | -0.176 | -0.285* |
| Uridine 5'-MP | +0.191 | +0.360** | +0.140 | +0.258* |
| Uridine | +0.369** | +0.348** | +0.295* | +0.107 |
| UDP-N-acetylglucosamine | +0.179 | +0.335** | +0.115 | +0.239* |
| UDP-galactose | +0.159 | +0.266* | 0.123 | +0.188 |
| Uracil | -0.218 | -0.408*** | -0.134 | -0.230 |
| Orotic acid | -0.312** | -0.229 | -0.262* | -0.053 |

^1^Significant effects are indicated in bold.

^2^Latency is in seconds, and distance moved is in cm. 0.05 > P > 0.01: *; 0.01 > P > 0.001: **; P < 0.001: ***

**Supplementary Table 10C.** Association (Spearman correlations) between visible platform water maze activity level (1 – 6 refers to trial number) and nucleotide and their metabolites levels in the hippocampus in the standard and extended exposure experiment^1,2^.

| Nucleotide | Latency 1 (sec) | Latency 2 (sec) | Latency 3 (sec) | Latency 4 (sec) | Latency 5 (sec) | Latency 6 (sec) |
| --- | --- | --- | --- | --- | --- | --- |
| ADP | +0.151 | +0.164 | +0.089 | +0.111 | +0.198 | +0.280* |
| Adenosine 3',5'-DP | +0.162 | +0.236* | +0.124 | +0.131 | +0.263* | +0.257* |
| AMP | +0.112 | +0.255* | +0.211 | +0.017 | +0.139 | +0.167 |
| 2',3'-cyclic AMP | -0.299* | -0.088 | -0.113 | -0.240* | -0.348** | -0.311** |
| 5'-Methylthioadenosine | +0.245* | +0.204 | +0.135 | +0.213 | +0.288* | +0.234* |
| N6-(delta2-Isopentenyl)-adenine | -0.227 | -0.243* | -0.239* | -0.380** | -0.235* | -0.111 |
| GMP | +0.010 | +0.241* | +0.173 | -0.002 | +0.081 | +0.116 |
| 2'-Deoxyguanosine 5'-MP | -0.244* | -0.213 | -0.145 | -0.305* | -0.268* | -0.281* |
| Guanine | +0.186 | +0.035 | +0.034 | +0.093 | +0.216 | +0.259* |
| IMP | +0.199 | +0.229 | +0.123 | +0.239* | +0.238* | +0.283* |
| Inosine | +0.243* | +0.254* | +0.197 | +0.268* | +0.304** | +0.234* |
| Hypoxanthine | +0.097 | +0.167 | +0.064 | +0.239* | +0.242* | +0.214 |
| Xanthine | -0.109 | -0.033 | -0.163 | -0.010 | +0.025 | +0.038 |
| Uric acid | -0.172 | -0.309** | -0.227 | -0.323** | -0.188 | -0.131 |
| Allantoin | -0.002 | +0.082 | -0.064 | +0.029 | +0.222 | +0.203 |
| CMP | +0.071 | +0.286* | +0.191 | +0.031 | +0.093 | +0.095 |
| Deoxycytidine | -0.203 | -0.085 | -0.046 | -0.213 | -0.316** | -0.199 |
| Cytosine | -0.127 | -0.274* | -0.105 | -0.138 | -0.201 | -0.187 |
| Thymine | -0.313** | -0.306** | -0.302* | -0.243* | -0.275* | -0.244* |
| Uridine 5'-MP | +0.067 | +0.241* | +0.192 | +0.028 | +0.131 | +0.136 |
| Uridine | +0.242* | +0.253* | +0.192 | +0.342** | +0.348** | +0.277 |
| UDP-N-acetylglucosamine | +0.255* | +0.209 | +0.218 | +0.124 | +0.112 | +0.122 |
| UDP-galactose | +0.234* | +0.167 | +0.164 | +0.154 | +0.182 | +0.207 |
| Uracil | -0.237* | -0.226 | -0.226 | -0.111 | -0.165 | -0.145 |
| Orotic acid | -0.151 | -0.256* | -0.187 | -0.371** | -0.354** | -0.287* |

^1^Significant effects are indicated in bold.

^2^Latency is in seconds. 0.05 > P > 0.01: *; 0.01 > P > 0.001: **; P < 0.001: ***

**Supplementary Table 10D.** Association (Spearman correlations) between fear conditioning (1 – 4 refers to trial number) and nucleotide and their metabolites levels in the hippocampus in the standard exposure experiment^1,2^.

| Nucleotide | Tone freeze 1 | Tone freeze 2 | Tone freeze 3 | Tone freeze 4 | Shock motion 1 | Shock motion 2 | Shock motion 3 | Shock motion 4 | Cued Fear Tone Freeze |
| --- | --- | --- | --- | --- | --- | --- | --- | --- | --- |
| ADP | +0.061 | -0.048 | -0.066 | -0.047 | -0.231 | -0.236 | -0.354 | -0.324 | -0.202 |
| Adenosine 3',5'-DP | +0.141 | +0.041 | -0.092 | -0.052 | -0.285 | -0.284 | -0.459*** | -0.345* | -0.209 |
| AMP | -0.299* | +0.160 | +0.116 | +0.041 | +0.263 | +0.295* | +0.077 | +0.102 | +0.096 |
| 2',3'-cyclic AMP | -0.089 | +0.053 | +0.118 | +0.225 | +0.119 | +0.132 | +0.112 | +0.122 | +0.160 |
| 5'-Methylthioadenosine | +0.258 | -0.037 | +0.006 | -0.218 | -0.285 | -0.215 | -0.344* | -0.308 | -0.106 |
| N6-(delta2-Isopentenyl)-adenine | -0.250 | +0.195 | +0.285 | +0.336* | +0.138 | +0.228 | +0.171 | +0.183 | +0.244 |
| GMP | -0.332* | +0.155 | +0.118 | +0.147 | +0.307* | +0.382** | +0.159 | +0.241 | +0.231 |
| 2'-Deoxyguanosine 5'-MP | -0.148 | +0.103 | +0.272 | +0.274 | -0.043 | +0.238 | +0.052 | +0.042 | +0.241 |
| Guanine | +0.091 | +0.076 | -0.081 | +0.054 | -0.217 | -0.354* | -0.404** | -0.288 | -0.074 |
| IMP | +0.166 | -0.119 | -0.234 | -0.215 | -0.307* | -0.340* | -0.327* | -0.242 | -0.172 |
| Inosine | +0.375* | -0.085 | -0.248 | -0.099 | -0.246 | -0.373* | -0.427** | -0.283 | -0.195 |
| Hypoxanthine | +0.354* | +0.005 | -0.257 | -0.047 | -0.434** | -0.412** | -0.443** | -0.314* | -0.285 |
| Xanthine | +0.206 | -0.053 | -0.143 | +0.075 | -0.267 | -0.209 | -0.295* | -0.120 | -0.211 |
| Uric acid | -0.106 | +0.137 | +0.196 | +0.297* | -0.030 | +0.169 | -0.054 | +0.067 | +0.189 |
| Allantoin | +0.133 | +0.009 | -0.288 | -0.049 | -0.198 | -0.083 | -0.306* | -0.044 | -0.273 |
| CMP | -0.213 | +0.209 | +0.129 | +0.173 | +0.238 | +0.348* | +0.104 | +0.152 | +0.178 |
| Deoxycytidine | -0.104 | +0.215 | +0.188 | +0.074 | +0.285 | +0.065 | +0.027 | +0.253 | +0.281 |
| Cytosine | -0.220 | +0.008 | -0.042 | +0.009 | -0.054 | +0.130 | +0.084 | +0.022 | -0.042 |
| Thymine | -0.103 | +0.015 | -0.054 | +0.158 | -0.001 | +0.180 | +0.094 | +0.105 | +0.030 |
| Uridine 5'-MP | -0.212 | +0.179 | +0.216 | +0.067 | +0.204 | +0.314 | +0.001 | +0.146 | +0.225 |
| Uridine | +0.277 | -0.239 | -0.303 | -0.093 | -0.130 | -0.298 | -0.117 | -0.315* | -0.235 |
| UDP-N-acetylglucosamine | +0.109 | +0.176 | +0.540*** | +0.299 | +0.135 | +0.128 | +0.124 | +0.086 | +0.430** |
| UDP-galactose | +0.108 | +0.094 | +0.083 | -0.004 | -0.342* | -0.266 | -0.382** | -0.442** | -0.108 |
| Uracil | +0.229 | +0.090 | -0.078 | +0.063 | -0.108 | -0.164 | -0.097 | -0.137 | -0.184 |
| Orotic acid | -0.175 | +0.258 | +0.435** | +0.362* | +0.218 | +0.380 | +0.229 | +0.122 | +0.485*** |

^1^Significant effects are indicated in bold.

^2^Freeze is in seconds, freeze (%) refers to % of time frozen, motion is in cm.

0.05 > *P* > 0.01: *; 0.01 > *P* > 0.001: **; *P* < 0.001: ***

**Supplementary Table 11A.** Association (Spearman correlations) between open field test and amino acid/peptide levels in the hippocampus in the standard and extended exposure experiment^1,2^.

| Amino acid/peptide | Distance moved 1 | Distance moved 2 | Center duration 1 | Center duration 2 |
| --- | --- | --- | --- | --- |
| L-Alanine | -0.452*** | -0.398*** | +0.240* | +0.241* |
| Histidine | +0.421*** | +0.409*** | -0.238* | -0.271* |
| 3-Methylhistidine | +0.516*** | +0.533*** | -0.338* | -0.305* |
| Urocanic acid | +0.469*** | +0.456*** | -0.293* | -0.197 |
| Leucine | +0.469*** | +0.473*** | -0.344** | -0.343** |
| N-Acetyl leucine | +0.506*** | +0.461*** | -0.332** | -0.357** |
| Lysine | +0.470*** | +0.506*** | -0.386*** | -0.370** |
| N6,N6,N6-Trimethyl-lysine | +0.389*** | +0.384** | -0.328** | -0.264* |
| 5-Aminopentanoic acid | +0.452*** | +0.451*** | -0.362** | -0.359** |
| Aminoadipic acid/ methyl-L-glutarate | -0.223 | -0.206 | +0.082 | +0.060 |
| Pipecolic acid | +0.526*** | +0.465*** | -0.295* | -0.244* |
| Methionine | +0.465*** | +0.471*** | -0.390*** | -0.377 |
| N-Acetyl-methionine | +0.372** | +0.378** | -0.260* | -0.212 |
| L-Aminocyclo-propane-carboxylate | +0.350** | +0.317** | -0.302* | -0.313** |
| Phenylalanine | +0.480*** | +0.478*** | -0.356** | -0.377** |
| N-Acetyl-phenylalanine | +0.492*** | +0.450*** | -0.393*** | -0.354 |
| Proline | +0.418*** | +0.461*** | -0.245* | -0.236* |
| 5-oxo-proline | +0.126 | +0.199 | -0.264* | -0.132 |
| 4-guanidino-butonoate | -0.050 | -0.052 | -0.121 | -0.236* |
| Serine | -0.283 | -0.232 | +0.164 | +0.155 |
| N-Acetylserine | +0.543*** | +0.545*** | -0.353** | -0.297* |
| Threonine | +0.422*** | +0.415*** | -0.395*** | -0.389*** |
| Tryptophan | +0.457*** | +0.435*** | -0.427*** | -0.413*** |
| Tyrosine | +0.462*** | +0.437*** | -0.357** | -0.346** |
| 3,4-dihydroxyphenyl-acetate | +0.473*** | +0.417*** | -0.275* | -0.292* |
| Valine | +0.502*** | +0.519*** | -0.374** | -0.340** |
| Creatine | -0.447*** | -0.398*** | +0.254* | +0.256* |
| Creatinine | -0.352** | -0.288* | +0.147 | +0.090 |
| Betaine | +0.400*** | +0.426*** | -0.373** | -0.315** |
| Carnosine | -0.517*** | -0.512*** | +0.250* | +0.288* |
| Glutathione | -0.489*** | -0.415*** | +0.296* | +0.324** |

^1^Significant effects are indicated in bold.

^2^Center duration is in seconds, and distance moved is in cm.

0.05 > *P* > 0.01: *; 0.01 > *P* > 0.001: **; *P* < 0.001: ***

**Supplementary Table 11B.** Association (Spearman correlations) between visible platform water maze activity level (1 – 4 refers to trial number) and amino acid/peptide levels in the hippocampus in the standard and extended exposure experiment^1,2^.

| Amino acid/peptide | Latency 1 (sec) | Latency 4 (sec) | Distance moved 1 | Distance moved 4 |
| --- | --- | --- | --- | --- |
| L-Alanine | +0.217 | +0.243* | +0.198 | +0.217 |
| Histidine | -0.201 | -0.360** | -0.102 | -0.362** |
| 3-Methylhistidine | -0.407*** | -0.369** | -0.297* | -0.312** |
| Urocanic acid | -0.369** | -0.383** | -0.260* | -0.338** |
| Leucine | -0.305** | -0.448*** | -0.207 | -0.410*** |
| N-Acetyl leucine | -0.258* | -0.426*** | -0.166 | -0.371** |
| Lysine | -0.353** | -0.446*** | -0.261* | -0.412*** |
| N6,N6,N6-Trimethyl-lysine | -0.366** | -0.344** | -0.255* | -0.360** |
| 5-Aminopentanoic acid | -0.326** | -0.437*** | -0.226 | -0.403*** |
| Aminoadipic acid/ methyl-L-glutarate | +0.029 | +0.097 | +0.073 | +0.077 |
| Pipecolic acid | -0.329** | -0.261* | -0.263* | -0.186 |
| Methionine | -0.356** | -0.427*** | -0.260* | -0.385** |
| N-Acetyl-methionine | -0.336** | -0.350** | -0.256* | -0.374** |
| L-Aminocyclo-propane-carboxylate | -0.297* | -0.305** | -0.221 | -0.326** |
| Phenylalanine | -0.278* | -0.369** | -0.192 | -0.321** |
| N-Acetyl-phenylalanine | -0.277* | -0.399*** | -0.194 | -0.350** |
| Proline | -0.214 | -0.389*** | -0.121 | -0.378** |
| 5-oxo-proline | -0.114 | -0.267* | -0.068 | -0.220 |
| 4-guanidino-butonoate | -0.169 | +0.029 | -0.141 | +0.085 |
| Serine | +0.212 | +0.126 | +0.180 | +0.071 |
| N-Acetylserine | -0.356** | -0.398*** | -0.248* | -0.340** |
| Threonine | -0.387*** | -0.392*** | -0.315** | -0.342** |
| Tryptophan | -0.272* | -0.412*** | -0.175 | -0.369** |
| Tyrosine | -0.296* | -0.424*** | -0.199 | -0.398*** |
| 3,4-dihydroxyphenyl-acetate | -0.272* | -0.247* | -0.213 | -0.215 |
| Valine | -0.309** | -0.440*** | -0.229 | -0.412*** |
| Creatine | +0.209 | +0.269* | +0.201 | +0.236* |
| Creatinine | +0.270** | +0.195 | +0.254* | +0.190 |
| Betaine | -0.393*** | -0.360** | -0.315** | -0.301* |
| Carnosine | +0.252** | +0.348** | +0.169 | +0.245* |
| Glutathione | +0.264** | +0.312** | +0.207 | +0.216 |

^1^Significant effects are indicated in bold.

^2^Latency is in seconds, and distance moved is in cm. 0.05 > P > 0.01: *; 0.01 > P > 0.001: **; P < 0.001: ***

**Supplementary Table 11C.** Association (Spearman correlations) between visible platform water maze activity level (1 – 6 refers to trial number) and amino acid/peptide levels in the hippocampus in the standard and extended exposure experiment^1,2^.

| Amino acid/peptide | Latency 1 (sec) | Latency 2 (sec) | Latency 3 (sec) | Latency 4 (sec) | Latency 5 (sec) | Latency 6 (sec) |
| --- | --- | --- | --- | --- | --- | --- |
| L-Alanine | +0.135 | +0.128 | +0.026 | +0.127 | +0.215 | +0.266* |
| Histidine | -0.189 | -0.260* | -0.184 | -0.122 | -0.221 | -0.170 |
| 3-Methylhistidine | -0.238* | -0.252* | -0.223 | -0.335** | -0.244* | -0.160 |
| Urocanic acid | -0.260* | -0.210 | -0.096 | -0.304** | -0.326** | -0.285* |
| Leucine | -0.255* | -0.301* | -0.227 | -0.256* | -0.275* | -0.204 |
| N-Acetyl leucine | -0.168 | -0.354** | -0.328** | -0.334** | -0.236* | -0.191 |
| Lysine | -0.211 | -0.305** | -0.209 | -0.220 | -0.270* | -0.224 |
| N6,N6,N6-Trimethyl-lysine | -0.275* | -0.230 | -0.177 | -0.350** | -0.308** | -0.242* |
| 5-Aminopentanoic acid | -0.234* | -0.275* | -0.209 | -0.256* | -0.262* | -0.204 |
| Aminoadipic acid/ methyl-L-glutarate | -0.076 | -0.009 | -0.077 | -0.174 | +0.073 | +0.162 |
| Pipecolic acid | -0.258* | -0.250* | -0.175 | -0.347** | -0.270* | -0.300* |
| Methionine | -0.234* | -0.294* | -0.208 | -0.271* | -0.280* | -0.209 |
| N-Acetyl-methionine | -0.261* | -0.143 | -0.165 | -0.283* | -0.286* | -0.150 |
| L-Aminocyclo-propane-carboxylate | -0.174 | -0.208 | -0.125 | -0.191 | -0.302* | -0.234* |
| Phenylalanine | -0.203 | -0.218 | -0.158 | -0.171 | -0.215 | -0.159 |
| N-Acetyl-phenylalanine | -0.212 | -0.382** | -0.264* | -0.336** | -0.242* | -0.201 |
| Proline | -0.178 | -0.223 | -0.177 | -0.112 | -0.194 | -0.166 |
| 5-oxo-proline | -0.008 | -0.140 | -0.028 | +0.027 | -0.037 | -0.185 |
| 4-guanidino-butonoate | +0.032 | -0.109 | -0.172 | -0.186 | -0.132 | +0.051 |
| Serine | +0.134 | +0.120 | +0.019 | +0.217 | +0.088 | +0.073 |
| N-Acetylserine | -0.254* | -0.307** | -0.258* | -0.337** | -0.284 | -0.233 |
| Threonine | -0.225 | -0.291* | -0.200 | -0.339** | -0.312** | -0.260 |
| Tryptophan | -0.206 | -0.283* | -0.163 | -0.238* | -0.256* | -0.296* |
| Tyrosine | -0.242* | -0.274* | -0.196 | -0.245* | -0.264* | -0.220 |
| 3,4-dihydroxyphenyl-acetate | -0.127 | -0.233 | -0.176 | -0.295* | -0.250* | -0.216 |
| Valine | -0.234* | -0.274* | -0.215 | -0.238* | -0.242* | -0.190 |
| Creatine | +0.114 | +0.140 | +0.038 | +0.128 | +0.181 | +0.253* |
| Creatinine | +0.138 | -0.005 | -0.053 | +0.195 | +0.239* | +0.249* |
| Betaine | -0.211 | -0.322** | -0.191 | -0.434*** | -0.291* | -0.223 |
| Carnosine | +0.296* | +0.169 | +0.192 | +0.237* | +0.198 | +0.195 |
| Glutathione | +0.202 | +0.327** | +0.185 | +0.204 | +0.201 | +0.235* |

^1^Significant effects are indicated in bold.

^2^Latency is in seconds.

0.05 > *P* > 0.01: *; 0.01 > *P* > 0.001: **; *P* < 0.001: ***

**Supplementary Table 11D.** Association (Spearman correlations) between fear conditioning (1 – 4 refers to trial number) and amino acid/peptide levels in the hippocampus in the standard exposure experiment^1,2^.

| Amino acid/peptide | Tone freeze 1 | Tone freeze 2 | Tone freeze 3 | Tone freeze 4 | Shock motion 1 | Shock motion 2 | Shock motion 3 | Shock motion 4 | Cued Fear Tone Freeze |
| --- | --- | --- | --- | --- | --- | --- | --- | --- | --- |
| L-Alanine | +0.229 | +0.089 | -0.177 | -0.134 | -0.192 | -0.293* | -0.413** | -0.308* | -0.282 |
| Histidine | -0.049 | -0.028 | +0.133 | +0.239 | -0.191 | +0.007 | +0.012 | +0.062 | +0.032 |
| 3-Methylhistidine | -0.220 | +0.241 | +0.115 | +0.157 | +0.337* | +0.309* | +0.145 | +0.292* | +0.172 |
| Urocanic acid | -0.150 | -0.027 | +0.169 | +0.156 | +0.239 | +0.258 | +0.304* | +0.253 | +0.149 |
| Leucine | -0.144 | +0.055 | +0.164 | +0.263 | -0.068 | +0.120 | +0.095 | +0.190 | +0.054 |
| N-Acetyl leucine | -0.045 | +0.010 | +0.205 | +0.311* | -0.238 | +0.017 | -0.098 | -0.097 | +0.117 |
| Lysine | -0.110 | +0.054 | +0.120 | +0.266 | -0.120 | +0.080 | +0.057 | +0.090 | +0.119 |
| N6,N6,N6-Trimethyl-lysine | -0.166 | +0.230 | +0.186 | +0.221 | +0.161 | +0.194 | +0.047 | +0.174 | +0.207 |
| 5-Aminopentanoic acid | -0.157 | +0.133 | +0.167 | +0.241 | +0.048 | +0.173 | +0.162 | +0.191 | +0.102 |
| Aminoadipic acid/ methyl-L-glutarate | +0.018 | +0.183 | +0.081 | +0.003 | -0.014 | -0.018 | -0.205 | -0.099 | -0.060 |
| Pipecolic acid | -0.075 | +0.136 | +0.252 | +0.239 | +0.372* | +0.265 | +0.248 | +0.181 | +0.344* |
| Methionine | -0.149 | +0.135 | +0.206 | +0.252 | +0.055 | +0.160 | +0.104 | +0.176 | +0.183 |
| N-Acetyl-methionine | -0.333* | +0.008 | +0.190 | +0.314* | -0.049 | +0.230 | +0.169 | +0.113 | +0.204 |
| L-Aminocyclo-propane-carboxylate | -0.069 | +0.139 | +0.190 | +0.314* | +0.056 | +0.157 | +0.120 | +0.141 | +0.155 |
| Phenylalanine | -0.141 | +0.095 | +0.119 | +0.222 | +0.000 | +0.052 | +0.127 | +0.069 | +0.149 |
| N-Acetyl-phenylalanine | -0.151 | -0.024 | +0.306* | +0.297* | -0.094 | +0.208 | +0.006 | +0.038 | +0.181 |
| Proline | -0.003 | -0.036 | +0.004 | +0.248 | -0.196 | -0.002 | -0.035 | +0.018 | -0.057 |
| 5-oxo-proline | +0.031 | -0.085 | -0.188 | +0.052 | -0.199 | -0.090 | -0.083 | -0.237 | -0.326* |
| 4-guanidino-butonoate | -0.014 | +0.209 | +0.056 | +0.011 | +0.220 | +0.086 | -0.029 | -0.134 | -0.004 |
| Serine | +0.169 | +0.008 | -0.105 | +0.179 | -0.217 | -0.122 | -0.135 | -0.119 | -0.114 |
| N-Acetylserine | -0.176 | +0.169 | +0.154 | +0.262 | +0.219 | +0.309* | +0.129 | +0.257 | +0.175 |
| Threonine | -0.088 | +0.185 | +0.251 | +0.342* | +0.230 | +0.256 | +0.217 | +0.154 | +0.227 |
| Tryptophan | -0.131 | +0.026 | +0.232 | +0.295* | +0.025 | +0.239 | +0.203 | +0.100 | +0.127 |
| Tyrosine | -0.108 | +0.138 | +0.211 | +0.245 | +0.026 | +0.165 | +0.102 | +0.174 | +0.187 |
| 3,4-dihydroxyphenyl-acetate | -0.075 | +0.235 | +0.340* | +0.261 | +0.314* | +0.247 | +0.189 | +0.189 | +0.477*** |
| Valine | -0.151 | +0.051 | +0.124 | +0.267 | -0.013 | +0.171 | +0.160 | +0.216 | +0.116 |
| Creatine | +0.239 | +0.155 | -0.106 | -0.117 | -0.136 | -0.323( | -0.424** | -0.254 | -0.237 |
| Creatinine | +0.255 | -0.016 | -0.197 | -0.146 | -0.348* | -0.260 | -0.311* | -0.300 | -0.294* |
| Betaine | -0.073 | +0.183 | +0.227 | +0.096 | +0.148 | +0.208 | -0.070 | +0.103 | +0.135 |
| Carnosine | -0.027 | -0.139 | -0.054 | +0.060 | -0.229 | -0.108 | -0.120 | -0.258 | +0.008 |
| Glutathione | -0.180 | +0.016 | +0.110 | +0.145 | -0.346* | -0.049 | -0.083 | -0.395 | +0.075 |

^1^Significant effects are indicated in bold.

^2^Freeze is in seconds, freeze (%) refers to % of time frozen, motion is in cm. 0.05 > P > 0.01: *; 0.01 > P > 0.001: **; P < 0.001: ***

**Supplementary Table 12A.** Association (Spearman correlations) between open field test and lipid/carbohydrate/vitamin levels in the hippocampus in the standard and extended exposure experiment^1,2^.

| Metabolite | Distance moved 1 | Distance moved 2 | Center duration 1 | Center duration 2 |
| --- | --- | --- | --- | --- |
| Dodecanoic acid | +0.478*** | +0.444*** | -0.214 | -0.192 |
| Myristic acid | +0.319** | +0.259* | -0.085 | -0.181 |
| Palmitic acid | +0.290* | +0.241* | -0.161 | -0.241* |
| Palmitoleic acid | +0.495*** | +0.468*** | -0.268* | -0.286* |
| Heptadecanoic acid | +0.256* | +0.208 | -0.084 | -0.087 |
| Octadecenoic acid | +0.465*** | +0.394*** | -0.233 | -0.293* |
| Ethanolamine phosphate | -0.409*** | -0.484*** | +0.393*** | +0.368** |
| Phosphocholine chloride | -0.454*** | -0.457*** | +0.399*** | +0.388*** |
| Sphinganine | -0.030 | -0.078 | +0.065 | +0.054 |
| Sphingomyelin | -0.420*** | -0.504*** | +0.325** | +0.374** |
| Desmosterol | -0.150 | -0.246 | +0.283* | +0.177 |
| Citric acid | -0.412*** | -0.476*** | +0.313** | +0.271* |
| Methylmalonic acid/succinic acid | +0.413*** | +0.437*** | -0.258* | -0.190 |
| Fumaric acid | -0.392*** | -0.356** | +0.327** | +0.351** |
| Malic acid | -0.303* | -0.268* | +0.339** | +0.351** |
| Citrulline | +0.494*** | +0.515*** | -0.423*** | -0.397*** |
| Ornithine | +0.489*** | +0.497*** | -0.291* | -0.305** |
| p-Aminobenzoic acid | +0.450*** | +0.391*** | -0.284* | -0.210 |
| 4-Pyridoxic acid | +0.534*** | +0.510*** | -0.384** | -0.281* |
| Pantothenic acid | -0.103 | -0.079 | +0.119 | +0.306** |
| FAD | -0.343** | -0.362** | +0.269* | +0.355** |
| NAD | -0.457*** | -0.450*** | +0.254* | +0.262* |
| Nicotinamide | -0.513*** | -0.515*** | +0.348** | +0.299* |
| Nicotinic acid | +0.540*** | +0.499*** | -0.241* | -0.241* |
| Riboflavin | +0.453*** | +0.447*** | -0.354** | -0.347** |
| Lumichrome | +0.490*** | +0.473*** | -0.249* | -0.190 |

^1^Significant effects are indicated in bold.

^2^Center duration is in seconds, and distance moved is in cm.

0.05 > *P* > 0.01: *; 0.01 > *P* > 0.001: **; *P* < 0.001: ***

**Supplementary Table 12B.** Association (Spearman correlations) between visible platform water maze activity level (1 – 4 refers to trial number) and lipid/carbohydrate/vitamin levels in the hippocampus in the standard and extended exposure experiment^1,2^.

| Metabolite | Latency 1 (sec) | Latency 4 (sec) | Distance moved 1 | Distance moved 4 |
| --- | --- | --- | --- | --- |
| Dodecanoic acid | -0.243* | -0.316** | -0.183 | -0.307** |
| Myristic acid | -0.078 | -0.256* | -0.032 | -0.225 |
| Palmitic acid | -0.169 | -0.206 | -0.150 | -0.148 |
| Palmitoleic acid | -0.314** | -0.373** | -0.233 | -0.332** |
| Heptadecanoic acid | -0.140 | -0.146 | -0.117 | -0.106 |
| Octadecenoic acid | -0.249* | -0.327** | -0.208 | -0.274* |
| Ethanolamine phosphate | +0.262* | +0.278* | +0.211 | +0.158 |
| Phosphocholine chloride | +0.356** | +0.369** | +0.317** | +0.302* |
| Sphinganine | +0.220 | +0.289* | +0.129 | +0.248* |
| Sphingomyelin | +0.177 | +0.316** | +0.144 | +0.254* |
| Desmosterol | +0.176 | +0.163 | +0.136 | +0.166 |
| Citric acid | +0.298* | +0.282* | +0.240* | +0.231 |
| Methylmalonic acid/succinic acid | -0.298* | -0.379** | -0.207 | -0.347** |
| Fumaric acid | +0.324** | +0.226 | +0.290* | +0.194 |
| Malic acid | +0.263* | +0.208 | +0.229 | +0.141 |
| Citrulline | -0.418*** | -0.453*** | -0.301* | -0.411*** |
| Ornithine | -0.250* | -0.426*** | -0.168 | -0.368** |
| p-Aminobenzoic acid | -0.312** | -0.229 | -0.262* | -0.247* |
| 4-Pyridoxic acid | -0.291* | -0.309** | -0.226 | -0.276* |
| Pantothenic acid | -0.419*** | -0.389*** | -0.354** | -0.330** |
| FAD | -0.013 | +0.168 | -0.002 | +0.102 |
| NAD | +0.115 | +0.271* | +0.107 | +0.177 |
| Nicotinamide | +0.131 | +0.272* | +0.091 | +0.189 |
| Nicotinic acid | +0.316** | +0.407*** | +0.259* | +0.367** |
| Riboflavin | -0.313** | -0.344** | -0.213 | -0.277* |
| Lumichrome | -0.269* | -0.396*** | -0.190 | -0.309** |

^1^Significant effects are indicated in bold.

^2^Latency is in seconds, and distance moved is in cm.

0.05 > *P* > 0.01: *; 0.01 > *P* > 0.001: **; *P* < 0.001: ***

**Supplementary Table 12C.** Association (Spearman correlations) between visible platform water maze activity level (1 – 6 refers to trial number) and lipid/carbohydrate/vitamin levels in the hippocampus in the standard and extended exposure experiment^1,2^.

| Metabolite | Latency 1 (sec) | Latency 2 (sec) | Latency 3 (sec) | Latency 4 (sec) | Latency 5 (sec) | Latency 6 (sec) |
| --- | --- | --- | --- | --- | --- | --- |
| Dodecanoic acid | -0.194 | -0.263* | -0.209 | -0.249* | -0.222 | -0.181 |
| Myristic acid | -0.007 | -0.064 | -0.135 | -0.177 | -0.062 | -0.057 |
| Palmitic acid | -0.024 | -0.123 | -0.068 | -0.084 | -0.130 | -0.046 |
| Palmitoleic acid | -0.228 | -0.188 | -0.208 | -0.302* | -0.213 | -0.167 |
| Heptadecanoic acid | +0.044 | -0.085 | -0.022 | -0.062 | -0.093 | -0.090 |
| Octadecenoic acid | -0.146 | -0.217 | -0.187 | -0.264* | -0.211 | -0.179 |
| Ethanolamine phosphate | +0.194 | +0.264* | +0.218 | +0.060 | +0.118 | +0.039 |
| Phosphocholine chloride | +0.273* | +0.258* | +0.163 | +0.202 | +0.297* | +0.219 |
| Sphinganine | +0.131 | +0.176 | +0.259* | +0.336** | +0.189 | +0.020 |
| Sphingomyelin | +0.233* | +0.331** | +0.345** | +0.294* | +0.329** | +0.171 |
| Desmosterol | +0.150 | +0.058 | +0.037 | +0.106 | +0.167 | +0.175 |
| Citric acid | +0.224 | +0.105 | +0.032 | +0.129 | +0.205 | +0.202 |
| Methylmalonic acid/succinic acid | -0.373** | -0.302 | -0.254* | -0.284* | -0.279* | -0.245* |
| Fumaric acid | +0.096 | +0.187 | +0.113 | +0.212 | +0.220 | +0.198 |
| Malic acid | +0.093 | +0.132 | +0.044 | +0.173 | +0.179 | +0.122 |
| Citrulline | -0.247* | -0.315** | -0.217 | -0.317** | -0.287* | -0.260* |
| Ornithine | -0.216 | -0.320** | -0.246* | -0.219 | -0.212 | -0.119 |
| p-Aminobenzoic acid | -0.187 | -0.289* | -0.204 | -0.305** | -0.262* | -0.156 |
| 4-Pyridoxic acid | -0.274* | -0.304** | -0.267* | -0.422*** | -0.355** | -0.308** |
| Pantothenic acid | -0.046 | +0.004 | -0.085 | -0.099 | -0.062 | -0.043 |
| FAD | +0.023 | +0.142 | +0.050 | -0.039 | +0.070 | +0.109 |
| NAD | +0.053 | +0.153 | +0.127 | -0.052 | +0.150 | +0.167 |
| Nicotinamide | +0.170 | +0.176 | +0.113 | +0.200 | +0.262* | +0.204 |
| Nicotinic acid | -0.255* | -0.257* | -0.324** | -0.350** | -0.215 | -0.074 |
| Riboflavin | -0.222 | -0.310** | -0.292* | -0.349** | -0.185 | -0.132 |
| Lumichrome | -0.113 | -0.345** | -0.334** | -0.349** | -0.112 | -0.072 |

^1^Significant effects are indicated in bold.

^2^Latency is in seconds.

0.05 > *P* > 0.01: *; 0.01 > *P* > 0.001: **; *P* < 0.001: ***

**Supplementary Table 12D.** Association (Spearman correlations) between fear conditioning (1 – 4 refers to trial number) and lipid/carbohydrate/vitamin in the hippocampus in the standard exposure experiment^1,2^.

| Metabolite | Tone freeze 1 | Tone freeze 2 | Tone freeze 3 | Tone freeze 4 | Shock motion 1 | Shock motion 2 | Shock motion 3 | Shock motion 4 | Cued Fear Tone Freeze |
| --- | --- | --- | --- | --- | --- | --- | --- | --- | --- |
| Dodecanoic acid | -0.193 | +0.065 | +0.350* | +0.311* | -0.100 | +0.152 | +0.082 | +0.145 | +0.354* |
| Myristic acid | -0.102 | +0.208 | +0.405** | +0.291* | -0.036 | +0.040 | +0.099 | +0.040 | +0.350* |
| Palmitic acid | -0.090 | +0.187 | +0.163 | -0.012 | +0.127 | +0.044 | +0.144 | +0.218 | +0.170 |
| Palmitoleic acid | -0.158 | +0.259 | +0.326* | +0.314* | +0.200 | +0.219 | +0.154 | +0.223 | +0.372* |
| Heptadecanoic acid | -0.084 | +0.161 | +0.206 | +0.009 | +0.167 | +0.057 | +0.172 | +0.214 | +0.211 |
| Octadecenoic acid | -0.108 | +0.299* | +0.374* | +0.216 | +0.175 | +0.253 | +0.150 | +0.272 | +0.394** |
| Ethanolamine phosphate | -0.193 | +0.065 | +0.350* | +0.311* | -0.100 | +0.152 | +0.082 | +0.145 | +0.354* |
| Phosphocholine chloride | -0.003 | +0.272 | +0.336* | +0.097 | -0.095 | +0.001 | -0.193 | +0.113 | +0.241 |
| Sphinganine | +0.439** | +0.067 | -0.068 | -0.183 | -0.224 | -0.369* | -0.439** | -0.227 | -0.211 |
| Sphingomyelin | -0.291* | -0.328* | -0.057 | -0.037 | -0.035 | +0.235 | +0.328* | -0.070 | +0.130 |
| Desmosterol | +0.118 | -0.064 | +0.112 | -0.070 | +0.047 | -0.141 | +0.027 | -0.052 | +0.117 |
| Citric acid | +0.266 | +0.053 | +0.003 | +0.034 | -0.284 | -0.158 | -0.287 | -0.223 | -0.182 |
| Methylmalonic acid/succinic acid | -0.090 | -0.025 | +0.097 | +0.167 | -0.075 | +0.183 | +0.106 | +0.180 | +0.101 |
| Fumaric acid | +0.438** | -0.186 | -0.245 | -0.081 | -0.229 | -0.257 | -0.184 | -0.148 | -0.288 |
| Malic acid | +0.276 | -0.138 | -0.194 | +0.055 | -0.269 | -0.124 | -0.145 | -0.115 | -0.240 |
| Citrulline | -0.100 | +0.193 | +0.269 | +0.293* | +0.129 | +0.165 | +0.108 | +0.174 | +0.280 |
| Ornithine | -0.110 | -0.131 | -0.014 | +0.054 | -0.284 | -0.061 | -0.060 | +0.108 | -0.138 |
| p-Aminobenzoic acid | -0.319* | +0.039 | +0.134 | +0.175 | +0.055 | +0.238 | +0.191 | +0.232 | +0.148 |
| 4-Pyridoxic acid | -0.223 | +0.068 | +0.151 | +0.275 | +0.197 | +0.286 | +0.090 | +0.125 | +0.144 |
| Pantothenic acid | -0.079 | +0.032 | +0.164 | +0.216 | +0.015 | +0.008 | -0.076 | -0.052 | +0.152 |
| FAD | -0.075 | +0.164 | +0.190 | +0.214 | +0.071 | +0.139 | -0.087 | +0.017 | +0.117 |
| NAD | -0.091 | +0.128 | +0.198 | -0.049 | +0.037 | +0.131 | -0.095 | +0.070 | +0.071 |
| Nicotinamide | +0.283 | -0.034 | -0.196 | -0.188 | +0.088 | -0.023 | -0.021 | -0.138 | -0.236 |
| Nicotinic acid | -0.081 | +0.037 | +0.055 | -0.009 | +0.012 | +0.061 | -0.165 | +0.230 | +0.040 |
| Riboflavin | -0.042 | +0.245 | +0.061 | +0.127 | +0.061 | +0.116 | -0.099 | -0.009 | -0.055 |
| Lumichrome | -0.119 | -0.015 | +0.056 | +0.111 | -0.055 | +0.126 | -0.096 | -0.079 | -0.053 |

^1^Significant effects are indicated in bold.

^2^Freeze is in seconds, freeze (%) refers to % of time frozen, motion is in cm. 0.05 > P > 0.01: *; 0.01 > P > 0.001: **; P < 0.001: ***
