# Supplementary material for: ApoE isoform-dependent effects of xanthohumol on high fat diet-induced cognitive impairments and hippocampal metabolic pathways
Source: Front Pharmacol. 2022 Oct 3;13:954980. doi: 10.3389/fphar.2022.954980 (PMC9583926; doi:10.3389/fphar.2022.954980)
Supplement: Supplementary file 8 [file DataSheet1.docx]

**Supplementary Figure Legends**

**Suppl. Figure 1.** Body weights of WT (**A**), E3 (**B**), E4 (**C**) in the standard exposure experiment. **D.** Body weights of E3 mice in the extended exposure experiment.

**Suppl. Figure 2.** Behavioral performance in the open field and object recognition. **A.** Activity levels of WT, E3, and E4 mice in the open field in the standard exposure experiment. B. Measures of anxiety of WT, E3, and E4 mice in the open field in the standard exposure experiment. **C.** Object recognition of WT, E3, and E4 mice in the standard exposure experiment. **D.** Activity levels of E3 mice in the open field in the extended exposure experiment. **E.** Measures of anxiety of E3 mice in the open field in the extended exposure experiment. **F.** Object recognition of E3 mice in the extended exposure experiment.

**Suppl. Figure 3.** Swim speeds of the mice in the standard (**A**) and extended (**B**) exposure experiment.

**Suppl. Figure 4.** Performance of E3 mice in the probe trial in the extended exposure experiment. **A.** Time to reach the platform location in the first probe trial. **B.** Time to reach the platform location in the second probe trial. **C.** Cumulative distance to the platform location in the first probe trial. **D.** Cumulative distance to the platform location in the second probe trial. **E.** Percent time spent in the target quadrant in the first and second probe trial.

**Suppl. Figure 5.** Behavioral performance in the fear conditioning test. **A.** Activity levels during the baseline period, prior to the first tone. **p* < 0.05 versus WT mice. **B.** Average response to the shock during fear learning. **C.** Percent freezing during the tones in the fear conditioning test. **D.** Freezing during the ISIs in the fear conditioning test. **E.** Freezing during the contextual fear memory test. **F.** Freezing during the tone in the cued fear memory test.
